# DNA repair in cardiomyocytes is critical for maintaining cardiac function in mice

**DOI:** 10.1111/acel.13768

**Published:** 2023-02-08

**Authors:** Martine de Boer, Maaike te Lintel Hekkert, Jiang Chang, Bibi S. van Thiel, Leonie Martens, Maxime M. Bos, Marion G. J. de Kleijnen, Yanto Ridwan, Yanti Octavia, Elza D. van Deel, Lau A. Blonden, Renata M. C. Brandt, Sander Barnhoorn, Paula K. Bautista‐Niño, Ilona Krabbendam‐Peters, Rianne Wolswinkel, Banafsheh Arshi, Mohsen Ghanbari, Christian Kupatt, Leon J. de Windt, A. H. Jan Danser, Ingrid van der Pluijm, Carol Ann Remme, Monika Stoll, Joris Pothof, Anton J. M. Roks, Maryam Kavousi, Jeroen Essers, Jolanda van der Velden, Jan H. J. Hoeijmakers, Dirk J. Duncker

**Affiliations:** ^1^ Division of Experimental Cardiology, Department of Cardiology Thoraxcenter, Erasmus MC Rotterdam The Netherlands; ^2^ Department of Molecular Genetics, Erasmus MC Rotterdam The Netherlands; ^3^ Department of Vascular Surgery, Erasmus MC Rotterdam The Netherlands; ^4^ Division of Vascular Medicine and Pharmacology, Department of Internal Medicine, Erasmus MC Rotterdam The Netherlands; ^5^ Department of Genetic Epidemiology, Institute of Human Genetics University Hospital Münster Münster Germany; ^6^ Department of Epidemiology, Erasmus MC Rotterdam The Netherlands; ^7^ Department of Radiotherapy, Erasmus MC Rotterdam The Netherlands; ^8^ Department of Physiology, Amsterdam Cardiovascular Sciences Amsterdam UMC, Vrije Universiteit Amsterdam Amsterdam The Netherlands; ^9^ Centro de Investigaciones Fundación Cardiovascular de Colombia‐ FCV Bucaramanga Colombia; ^10^ Department of Clinical and Experimental Cardiology, Heart Center Academic Medical Center, University of Amsterdam Amsterdam The Netherlands; ^11^ I. Medizinische Klinik und Poliklinik University Clinic Rechts der Isar, Technical University of Munich Munich Germany; ^12^ DZHK (German Center for Cardiovascular Research), partner site Munich Heart Alliance Munich Germany; ^13^ Walter‐Brendel‐Centre for Experimental Medicine Ludwig Maximilian University of Munich Munich Germany; ^14^ Department of Molecular Genetics, Faculty of Health, Medicine and Life Sciences Maastricht University Maastricht The Netherlands; ^15^ Faculty of Science and Engineering Maastricht University Maastricht The Netherlands; ^16^ Department of Biochemistry, Cardiovascular Research Institute Maastricht Maastricht University Maastricht The Netherlands; ^17^ Netherlands Heart Institute Utrecht The Netherlands; ^18^ CECAD Forschungszentrum Universität zu Köln Köln Germany; ^19^ Princess Máxima Center for Pediatric Oncology, Genome Instability and Nutrition ONCODE Institute Utrecht The Netherlands

**Keywords:** apoptosis, cardiac function, congestive heart failure, DNA damage, DNA repair

## Abstract

Heart failure has reached epidemic proportions in a progressively ageing population. The molecular mechanisms underlying heart failure remain elusive, but evidence indicates that DNA damage is enhanced in failing hearts. Here, we tested the hypothesis that endogenous DNA repair in cardiomyocytes is critical for maintaining normal cardiac function, so that perturbed repair of spontaneous DNA damage drives early onset of heart failure. To increase the burden of spontaneous DNA damage, we knocked out the DNA repair endonucleases xeroderma pigmentosum complementation group G (XPG) and excision repair cross‐complementation group 1 (ERCC1), either systemically or cardiomyocyte‐restricted, and studied the effects on cardiac function and structure. Loss of DNA repair permitted normal heart development but subsequently caused progressive deterioration of cardiac function, resulting in overt congestive heart failure and premature death within 6 months. Cardiac biopsies revealed increased oxidative stress associated with increased fibrosis and apoptosis. Moreover, gene set enrichment analysis showed enrichment of pathways associated with impaired DNA repair and apoptosis, and identified TP53 as one of the top active upstream transcription regulators. In support of the observed cardiac phenotype in mutant mice, several genetic variants in the *ERCC1* and *XPG* gene in human GWAS data were found to be associated with cardiac remodelling and dysfunction. In conclusion, unrepaired spontaneous DNA damage in differentiated cardiomyocytes drives early onset of cardiac failure. These observations implicate DNA damage as a potential novel therapeutic target and highlight systemic and cardiomyocyte‐restricted DNA repair‐deficient mouse mutants as *bona fide* models of heart failure.

## Graphical abstract

Loss of DNA repair permitted normal heart development but subsequently caused progressive deterioration of cardiac function and dilation, resulting in overt congestive heart failure and premature death within 6 months.

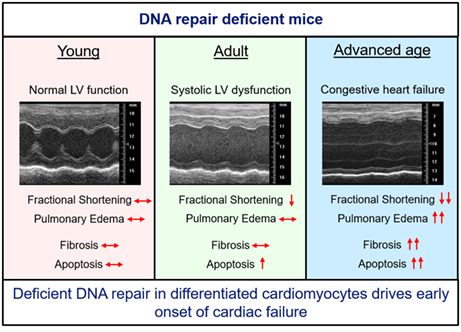

## 1 INTRODUCTION

Heart failure has become a global disease epidemic, particularly in the elderly (Bui et al., 2011; Lloyd‐Jones et al., 2002). Notwithstanding major advances in treatment, the exact molecular mechanisms underlying the pathogenesis of heart failure remain incompletely understood hampering a more mechanism‐based approach to effective treatment and prevention. Experimental and clinical evidence indicates that DNA damage, for example, due to oxidative stress or following chemotherapy (Octavia et al., 2012), is associated with heart failure (Bartunek et al., 2002; Higo et al., 2017; Shukla et al., 2010). In addition, accumulation of unrepaired DNA damage is linked with segmental premature ageing phenotypes in several organ systems (Hoeijmakers, 2009; Lopez‐Otin et al., 2013; Vermeij, Hoeijmakers, & Pothof, 2016). However, the precise effects of unrepaired endogenous DNA damage, and its role in the pathogenesis of heart failure, remain elusive. In the present study, we tested the hypothesis that DNA repair in cardiomyocytes is critical for maintaining normal cardiac function. For this purpose, we generated mice with genetically depleted DNA repair mechanisms in either all cell types or restricted to cardiomyocytes. XPG and ERCC1‐XPF, two major players in DNA repair, are structure‐specific endonucleases responsible for excision of DNA lesions in nucleotide excision repair (NER) and transcription‐coupled repair (TCR). Moreover, XPG is implicated in promoting base excision repair of oxidative DNA damage and double‐strand break (DSB) repair (Marteijn et al., 2014; Trego et al., 2016). ERCC1‐XPF is additionally involved in interstrand cross‐link repair, and sub‐pathways of DSB repair (Gregg et al., 2011). *Xpg* ^*−/−*^, *Ercc1* ^*−/−*^ and *Ercc1* ^*Δ/−*^ mice (and corresponding human syndromes) show many progressive progeroid characteristics (e.g., cachexia, neurodegeneration, osteoporosis and impaired growth) and a severely shortened lifespan (Barnhoorn et al., 2014; Weeda et al., 1997). In addition to these systemic mouse mutants, we generated mice with genetically depleted *Xpg* and *Ercc1* in differentiated cardiomyocytes using an alpha‐myosin heavy chain (*αMHC*)*‐*promoter‐driven *Cre* transgene (mice referred as *αMHC‐Xpg* ^*c/−*^ and *αMHC‐Ercc1* ^*c/−*^ respectively). The cardiomyocyte‐restricted mutants were employed to circumvent the potentially confounding influence on the heart of premature ageing in other organs associated with systemic loss of *Xpg* and *Ercc1*. Both systemic and cardiomyocyte‐restricted mutants were investigated to elucidate the cell‐autonomous effect of endogenous DNA damage on cardiac function.

## 2 RESULTS

### 2.1 Deficient DNA repair results in time‐dependent deterioration of global LV function and severely shortened lifespan in all mutants

*Ercc1* ^*−/−*^ mice showed severe growth retardation and a dramatically reduced lifespan. Consequently, these mice could only be studied at 8 weeks at which time point survival rate was 39% (Figure S1). We therefore also included *Ercc1* ^*Δ/−*^ mice, in which the Δ allele only partially inactivates the function of *Ercc1* due to a seven amino‐acid carboxy‐terminal truncation; consequently, lifespan, compared with *Ercc1* ^*−/−*^ mice, is extended to 4–6 months and *Ercc1* ^*Δ/−*^ mice exhibit a milder degenerative phenotype, consistent with a milder mutation (Weeda et al., 1997). To achieve cardiomyocyte‐restricted *Xpg* and *Ercc1* gene inactivation, mice with conditional *Xpg* and *Ercc1* alleles were crossed with a transgenic line with Cre recombinase under control of the αMHC promotor (Figure S2a,b). These mice were heterozygous for *Xpg* and *Ercc1*, respectively, in all cell types, except for cardiomyocytes, which were homozygous for loss of *Xpg* and *Ercc1*, and are referred to as *αMHC‐Xpg* ^*c/−*^ and *αMHC‐Ercc1* ^*c/−*^, respectively. Littermates, heterozygous for the floxed allele and the *Cre* transgene, were used as controls and are indicated as *αMHC‐Xpg* Ctrl and *αMHC‐Ercc1* Ctrl. Left ventricular (LV) dimension and function were indistinguishable between these mice and the control littermates heterozygous for the floxed allele only (Figure S3a), indicating that the presence of *Cre* recombinase itself did not lead to a heart failure phenotype. Control littermates heterozygous for *Xpg* or *Ercc1* in all cell types (referred to as *Xpg* ^*fl/−*^ *αMHC‐Cre* ^*−*^ and *Ercc1* ^*fl/−*^ *αMHC‐Cre* ^*−*^, Figure S2a,b) did not show cardiac alterations compared with control littermates wildtype in all cell types (referred to as *Xpg* ^*fl/+*^ *αMHC‐Cre* ^*−*^ and *Ercc1* ^*fl/+*^ *αMHC‐Cre* ^*−*^, Figures S2a,b and S3b), indicating that *Xpg* or *Ercc1* heterozygosity in all cell types did not affect cardiac function. This is compatible with the autosomal recessive nature of these genetic defects and the general absence of overt phenotypes in obligate carriers of defects in most human nucleotide excision repair disorders, such as Xeroderma Pigmentosum and Cockayne syndrome (Hoeijmakers, 2009; Vermeij, Hoeijmakers, & Pothof, 2016).

Myocardial *Xpg* and *Ercc1* mRNA expression levels in, respectively, *αMHC‐Xpg* ^*c/−*^ and *αMHC‐Ercc1* ^*c/−*^ mice, were significantly attenuated compared with corresponding control mice (Figure S4a).

**FIGURE 1:**
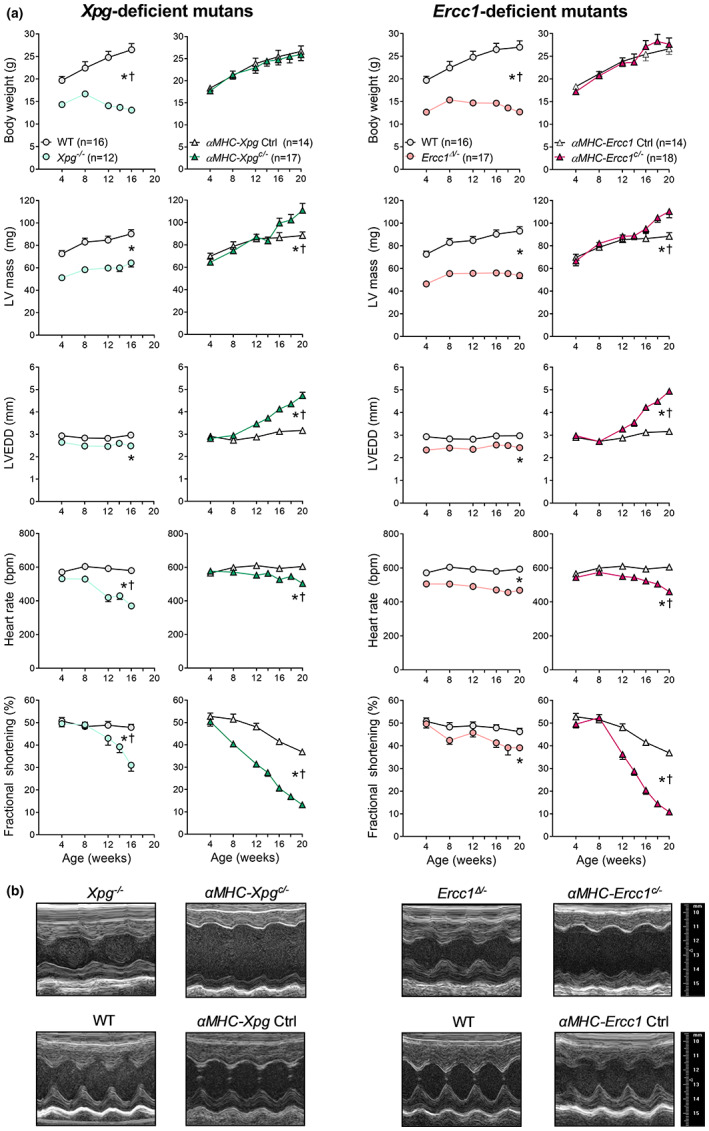
Impaired DNA repair resulted in time‐dependent deterioration of global LV function in all mutants (*Study I*, lifespan studies). (a) Effect of *Xpg* and *Ercc1* deficiency on body weight, LV mass and geometry and hemodynamic parameters during age. LV mass, left ventricular mass, calculated using the VisualSonics Cardiac Measurements Package; LVEDD, LV end‐diastolic lumen diameter. The number of animals is indicated in the body weight graph. All mutants have their own corresponding control littermates. Data are presented as mean ± SEM. (b) Representative LV short axis M‐mode images of the different DNA repair‐deficient mutants and corresponding control at age 16 weeks. *p < 0.05 vs. corresponding control; †*p* < 0.05 genotype × age using mixed linear model–repeated measures.

*Xpg* ^*−/−*^ and *Ercc1* ^*Δ/−*^ mice exhibited reduced growth, followed by body weight loss after 8 weeks of age, and premature death, in striking contrast to apparent normal body growth of *αMHC‐Xpg* ^*c/−*^ and *αMHC‐Ercc1* ^*c/−*^ mice (Figure 1a and Figure S4b,c).

Cardiomyocyte‐restricted inactivation of *Xpg* and *Ercc1* also reduced lifespan in both male and female mice, albeit less than in *Xpg* ^*−/−*^ and *Ercc1* ^*Δ/−*^ mice, respectively (Figure S4c,d), suggesting that defective DNA repair in the heart is not lifespan limiting in the systemic mutants. As reported previously (Vermeij, Dollé, et al., 2016; Vermeij, Hoeijmakers, & Pothof, 2016), the more severe repair defect in the absence of *Ercc1* relative to *Xpg* correlates with a shorter lifespan compared with *Xpg* inactivation, consistent with an inverse dose–response relationship between unrepaired DNA damage and lifespan (Figure S4c). Importantly, echocardiography at 4 weeks of age showed normal cardiac function in all mutants, indicating unaffected heart development (Figure 1a). Over time, all DNA repair‐deficient mutants gradually developed bradycardia and contractile dysfunction compared with corresponding control (Figure 1a,b), demonstrating that loss of DNA repair induces a degenerative process. Time‐dependent contractile deterioration was more severe in *αMHC‐Xpg* ^*c/−*^ and *αMHC‐Ercc1* ^*c/−*^ mice than in systemic mutants, demonstrated by progressive increases in LV end‐diastolic lumen diameter and decreases in fractional shortening starting after 8 weeks of age (Figure 1a,b). Systemic DNA repair‐deficient mutants display macro‐ and microvascular vasodilator dysfunction, which could potentially have contributed to the observed LV‐remodeling. Thus, similar to what we previously observed in *Ercc1* ^*Δ/*^ (Durik et al., 2012), 16‐week‐old *Xpg* ^*−/−*^ mice showed reductions in acetylcholine‐ and sodium nitroprusside‐induced aortic relaxation (Figure S5a). Importantly, however, no vascular dysfunction was observed in *αMHC‐Xpg* ^*c/−*^ mice (Figure S5b), despite severe cardiac dysfunction. This indicates that conditional disruption of *Xpg* in cardiomyocytes left vascular function unaffected and conversely that the perturbed vascular function observed in *Xpg* ^*−/−*^ mice did not contribute to the heart failure phenotype.

### 2.2 *Xpg‐* and *Ercc1‐*deficiency produces LV‐remodeling and LV dysfunction

**FIGURE 2:**
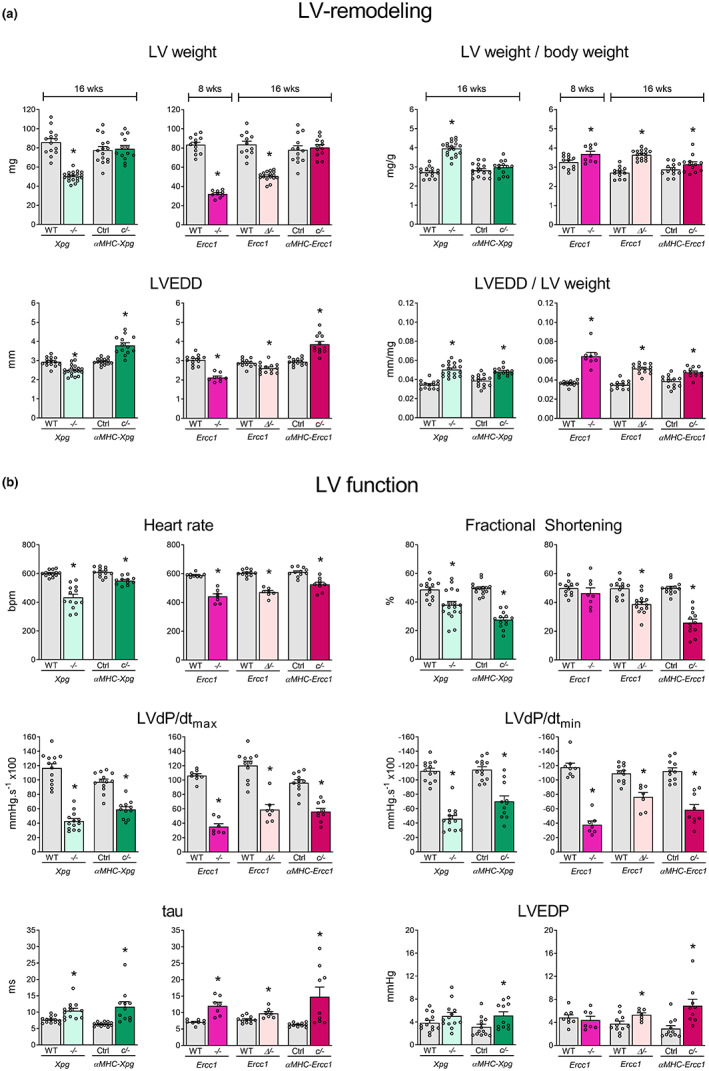
LV‐remodeling and LV dysfunction in DNA repair‐deficient mutants at a specific time point (*study II*). (a) Effect of *Xpg* and *Ercc1* deficiency on LV mass and geometry and (b), hemodynamic parameters in 16‐week‐old *Xpg* ^*−/−*^ and *αMHC‐Xpg* ^*c/−*^, 8‐week‐old *Ercc1* ^*−/−*^, 16‐week‐old *Ercc1* ^*Δ/−*^ and *αMHC‐Ercc1* ^*c/−*^ mice and corresponding control (*n* = 7–16 animals/group). LV weight, left ventricular weight; BW, body weight; LVEDD, LV end‐diastolic lumen diameter; LVdP/dt_max_, maximum rate of rise of LV pressure; LVdP/dt_min_, maximum rate of fall of LV pressure; tau, relaxation time constant; LVEDP, LV end‐diastolic pressure. Data are presented as mean ± SEM. *p < 0.05 vs. corresponding control by two‐way ANOVA followed by SNK post hoc testing.

Detailed analysis of LV function at age 16 weeks (except for *Ercc1* ^*−/−*^ mice that were studied at 8 weeks) revealed increased LV end‐diastolic lumen diameters (LVEDD, normalized to LV weight) in all DNA repair‐deficient mutants (Figure 2a). In addition, systolic LV function (fractional shortening, LVdP/dt_max_) and early (LVdP/dt_min_, tau) and late (LVEDP) diastolic LV function were markedly impaired (Figure 2b).

### 2.3 Increased cardiac expression of foetal genes in *Xpg* mutants

Based on the survival curves (Figure S4c) and the similar degree of cardiac dysfunction in *Xpg* ^*−/−*^ and *αMHC‐Xpg* ^*c/−*^ mice at 16 weeks (Figure 1), we elected to further study the *Xpg* mutants in more detail. LV dilation in 16‐week‐old *αMHC‐Xpg* ^*c/−*^ mice was accompanied by increased re‐expression of the foetal genes atrial natriuretic peptide (*Anp*), brain natriuretic peptide (*Bnp*) and α‐skeletal actin (*Acta1*), whereas in 16‐week‐old *Xpg* ^*−/−*^ mice, only *Anp* levels were increased (Figure S6a). At 8 weeks, expression of these marker genes was not yet elevated (Figure S6b), further supporting the degenerative (rather than developmental) nature of the disease process.

### 2.4 Altered cardiomyocyte contractile properties in *Xpg* ^*−/−*^ mice

**FIGURE 3:**
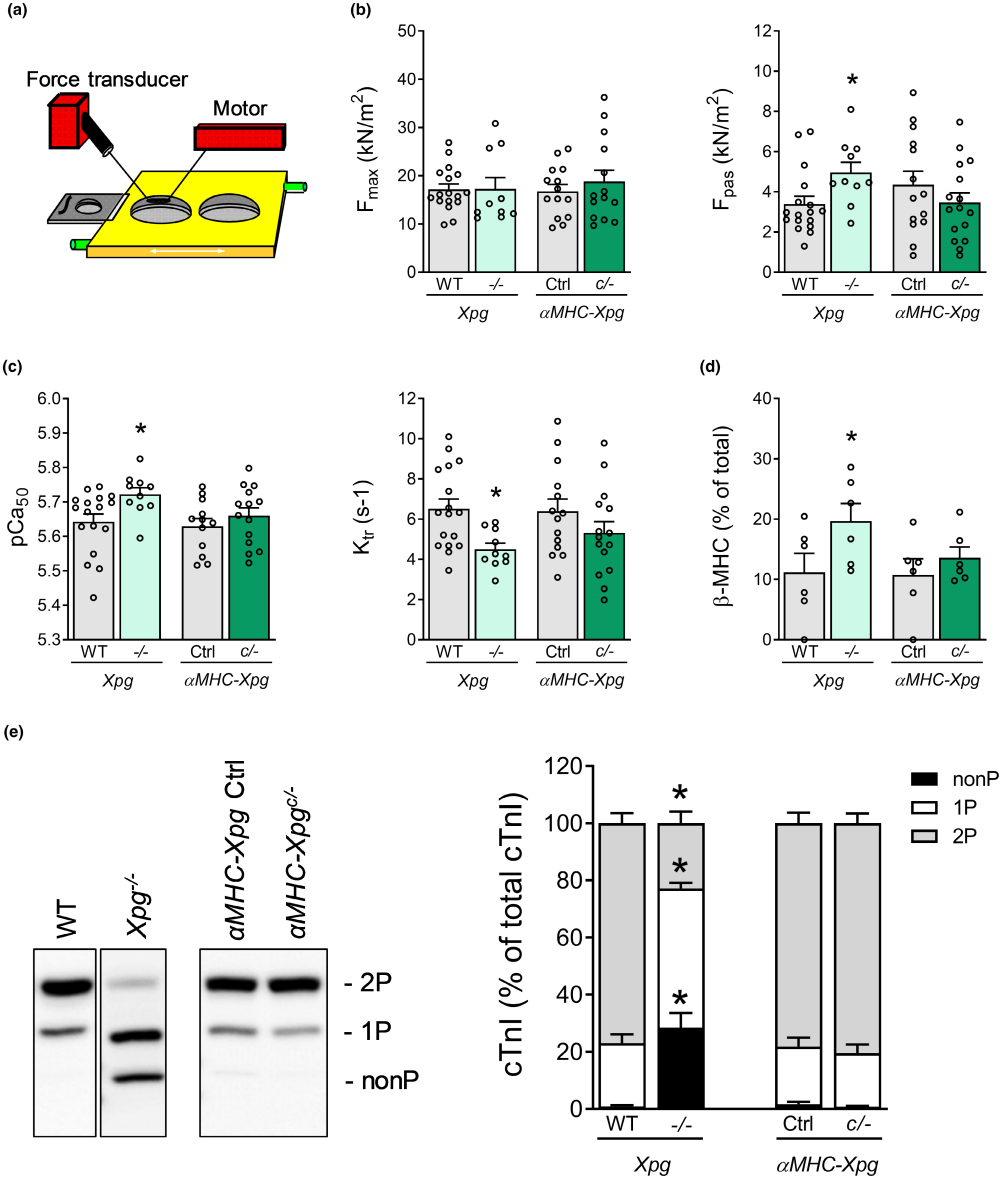
Altered cardiomyocyte contractile properties in 16‐week‐old *Xpg* ^*−/−*^ mice, but not in 16‐week‐old *αMHC‐Xpg* ^*c/−*^ mice (*Study II*). (a) Schematic experimental myocyte set‐up. (b) Isometric force measurements in single permeabilized cardiomyocytes (*n* = 6 animals/group; 1–4 cardiomyocytes/animal). *F*_max_, maximal force; *F*_pas_, passive force. (c) Determination of Ca^2+^ sensitivity of force (pCa_50_) and maximal rate of force redevelopment (K_tr_) (*n* = 6 animals/group; 1–4 cardiomyocytes/animal). (d) Myosin heavy chain isoform composition was determined by protein levels of myosin heavy chain isoforms (*n* = 6 animals/group). Beta‐myosin heavy chain (β‐MHC) content is expressed as % of total MHC. (e) Phos‐tag analysis of cardiac troponin I (cTnI) species, expressed as % of total phosphorylated cTnI (*n* = 6–7 animals/group). Phosphorylated cTnI species, of similar molecular weight, were separated into three forms: Non‐phosphorylated (nonP), mono‐phosphorylated (1P) and bis‐phosphorylated (2P). These forms were visualized with a specific antibody against troponin I, which recognizes non‐phosphorylated and phosphorylated forms. Data are presented as mean ± SEM. *p < 0.05 vs. corresponding control by two‐way ANOVA followed by SNK post hoc testing.

To explore whether functional properties of cardiomyocytes contributed to perturbed LV function, force measurements were performed in membrane‐permeabilized single LV cardiomyocytes isolated from *Xpg* mutant mice. Contractile properties of single cardiomyocytes obtained by mechanical isolation and attached to a force transducer and a piezoelectric motor (Figure 3a) were studied at a sarcomere length of 2.2 μm. Unexpectedly, maximum force generating capacity (F_max_) was maintained in cardiomyocytes from both *Xpg* mutants, indicating that the observed global systolic LV dysfunction was not due to a loss of force development of individual cardiomyocytes. A small elevation in passive tension (*F*_pas_) was observed in *Xpg* ^*−/−*^ mice only (Figure 3b). Similarly, in *Xpg* ^*−/−*^ mice, increases in myofilament Ca^2+^ sensitivity were noted (Figure 3c), which were accompanied by a lower protein kinase A (PKA)‐mediated phosphorylation of cardiac troponin I (cTnI), at the PKA sites Ser23/24 (Figure 3e; Figure S7). In addition, increased protein levels of β‐MHC and decreased maximal rate of force redevelopment (max K_tr_; changes typically associated with LV hypertrophy and failure (Hamdani et al., 2008)) were observed in *Xpg* mutants, reaching statistical significance in *Xpg* ^*−/−*^ mice (Figure 3c,d). These changes explain, at least partly, the diastolic dysfunction in the *Xpg* mutants.

### 2.5 Enhanced extracellular matrix turnover and increased levels of cell loss in *Xpg* mutants

**FIGURE 4:**
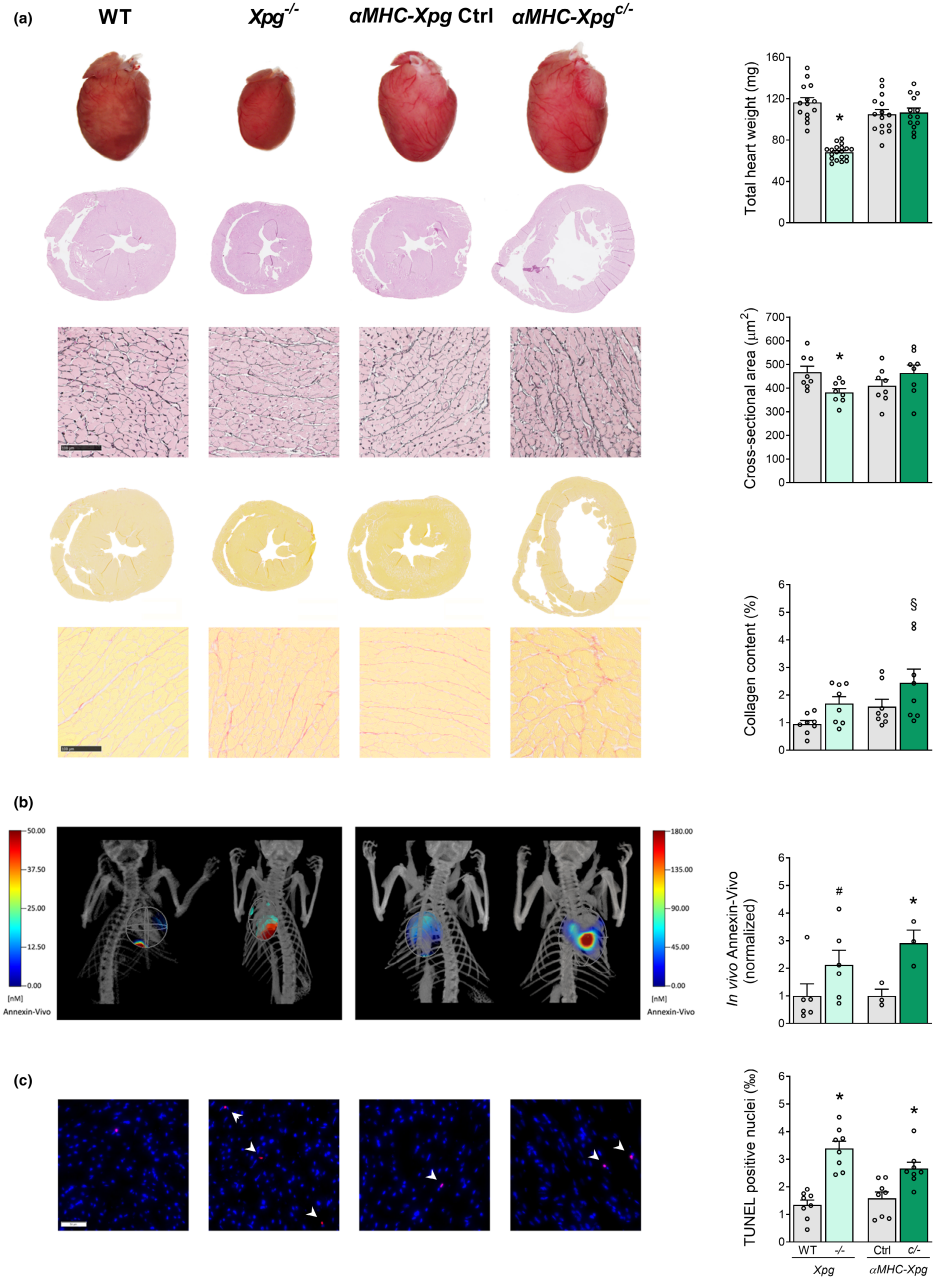
Characterization of LV phenotype in 16‐week‐old *Xpg* mutants (*Study II*). (a) Representative images of whole hearts and haematoxylin and eosin‐stained two‐chamber view sections and examination of total heart weight (*n* = 13–19 animals/group). Representative gomori‐ and picro‐sirius red‐stained LV sections and quantification of, respectively, cardiomyocyte cross‐sectional area and myocardial collagen content (*n* = 8 animals/group). (b) Representative images and quantification of the in vivo visualization of the membrane‐bound phospholipid phosphatidylserine to detect early apoptosis using FMT combined with μCT (*n* = 3–6 animals/group). (c) Representative TUNEL‐stained LV sections and quantification of TUNEL‐positive nuclei (indicated by arrows) to detect late apoptotic cells (*n* = 8 animals/group). Data are presented as mean ± SEM. *p < 0.05, §*p* = 0.062, #*p* = 0.087 vs. corresponding control by two‐way ANOVA followed by SNK post hoc testing.

Histological analysis revealed a small decrease in cardiomyocyte cross‐sectional area in *Xpg* ^*−/−*^, but not in *αMHC‐Xpg* ^*c/−*^ mice, paralleling changes in heart weight (Figure 4a). Collagen fraction tended to increase (Figure 4a) and extracellular matrix turnover was enhanced (Figure S8a,b), which may have contributed not only to the cardiac remodelling but also to the fractionated QRS pattern observed on surface ECG analysis in vivo (Figure S8c), suggestive of cardiac conduction delay (Rizzo et al., 2012). Moreover, *αMHC‐Xpg* ^*c/−*^ mice displayed bradycardia and significantly increased PR interval, *P* duration and QRS interval (Figure S8d). These ECG parameters were already affected at the age of 8 weeks, while these parameters were unaffected at the age of 4 weeks in *αMHC‐Xpg* ^*c/−*^ mice (Figure S8e). Collagen content was still unchanged in hearts of 8‐week‐old *αMHC‐Xpg* ^*c/−*^ mice (*αMHC‐Xpg* ^*c/−*^ 1.54 ± 0.13%, *n* = 5 vs. corresponding control 1.39 ± 0.24%, *n* = 5; *p* = 0.60), indicating that cardiac conduction abnormalities preceded the development of structural alterations and were not the consequence of the latter. While (atrio‐)ventricular conduction was not affected in *Xpg* ^*−/−*^ mice, significant QT prolongation was observed. This was likely the consequence of the low heart rate in these mice since no differences were observed in QT interval corrected for heart rate (QTc; Figure S8d).

To assess the purported role of oxidative stress in heart failure, basal superoxide generation was measured in homogenates using lucigenin‐enhanced chemiluminescence. Both NOX activity (using NADPH as substrate) and NOX‐dependent superoxide production (using NOX inhibitor VAS2870) were increased, particularly in *Xpg* ^*−/−*^ mice, resulting in elevations in total superoxide production (Figure S9). Since oxidative stress is a major cause of apoptosis (Kannan & Jain, 2000), molecular imaging was performed to determine the level of apoptosis in the in vivo heart using the near‐infrared fluorescent Annexin‐Vivo™ 750 probe, combined with contrast‐enhanced μCT for anatomical reference. This probe recognizes and attaches to phosphatidylserine residues displayed on the cell surface during early stages of apoptosis. Both *Xpg* mutants showed increased in vivo levels of apoptosis in the heart (Figure 4b). Finally, *Xpg* ^*−/−*^ and *αMHC‐Xpg* ^*c/−*^ mice showed elevated levels of TUNEL‐positive nuclei in LV myocardium (Figure 4c), indicating increased apoptotic cell death. Loss of cells likely contributed to the observed deterioration in cardiac function. Surprisingly, this loss was not accompanied by increased myocardial expression of the cyclin‐dependent kinase inhibitors *p21* and *p16* in *Xpg* mutants compared with control. These observations contrast with our present and previous (Barnhoorn et al., 2014; Durik et al., 2012) findings in the liver of *Xpg* ^*−/−*^ mice, which exhibited increased expression of *p21* and *p16* (Figure S10a). Moreover, evaluation of phosphorylation of the H2A.X histone (γH2A.X), an indicator of DNA damage (Greenberg, 2008), revealed increased γH2A.X‐positive nuclei in *Xpg* ^*−/−*^ liver compared with control, but not in *Xpg* ^*−/−*^ and *αMHC‐Xpg* ^*c/−*^ hearts (Figure S10b). These observations could be interpreted to suggest that myocardial cells appear highly sensitive to DNA damage, responding with apoptotic cell death.

### 2.6 Congestive heart failure, enhanced fibrosis and dramatically elevated levels of cell loss in advanced age *αMHC‐Xpg* ^*c*^ ^*/−*^ mice

**FIGURE 5:**
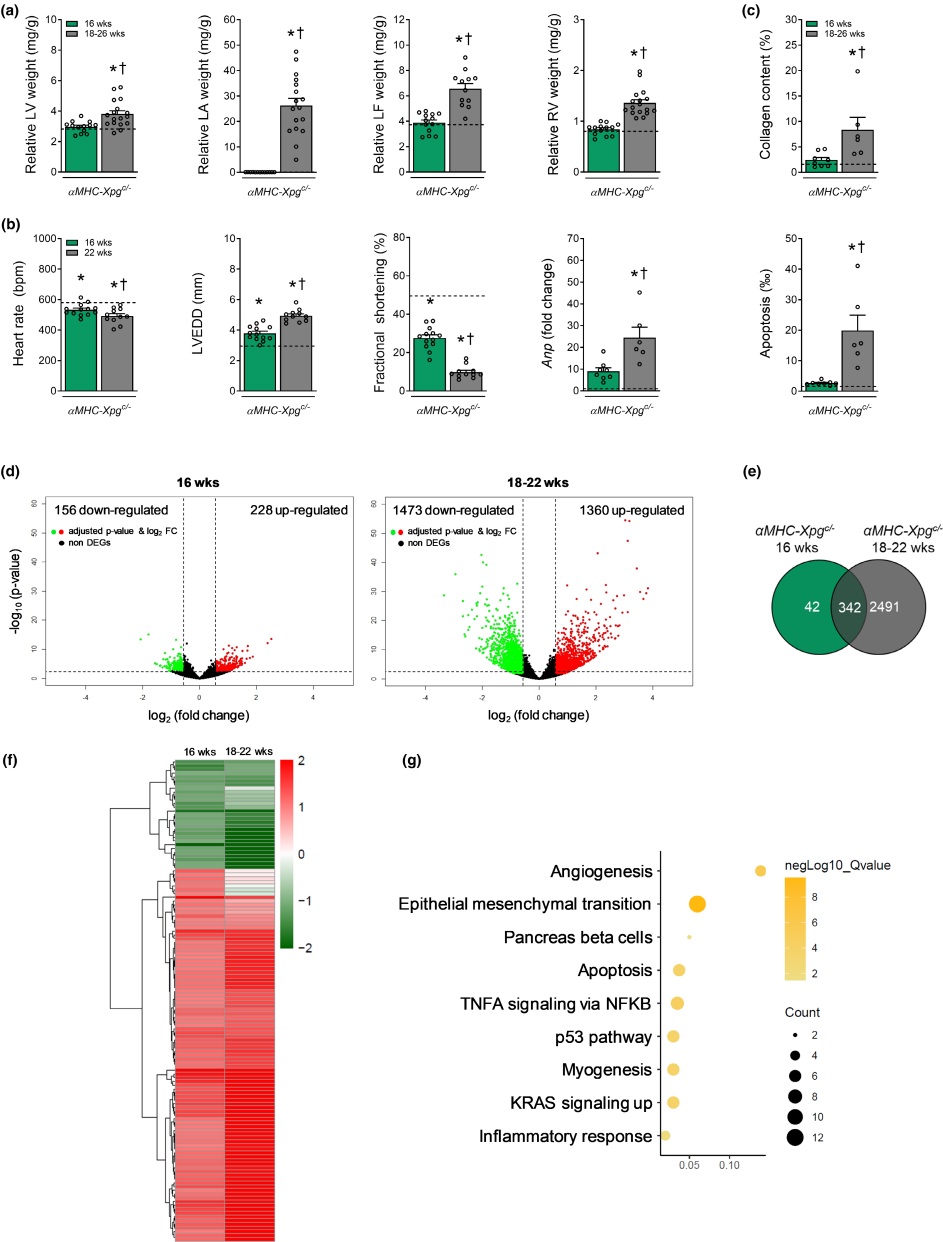
Progressive congestive heart failure and activation of TP53 in advanced age *αMHC‐Xpg* ^*c/−*^ mice. (a) Cardiomyocyte‐restricted loss of *Xpg* resulted in increased LV, LA, LF and RV weight during aging (*n* = 12–17 animals/group). LV weight, left ventricular weight; LA weight, left atrial weight; LF weight, lung fluid weight; RV weight, right ventricular weight. Weights are normalized to body weight. (b) Echocardiographic analysis at age 22 weeks (*Study I*, lifespan study) revealed progressive enlarged LV lumen diameter and aggravated loss of factional shortening compared with 16‐week‐old *αMHC‐Xpg* ^*c/−*^ mice and corresponding control (*Study II*) (*n* = 11–15 animals/group). In addition, quantitative real‐time PCR analysis revealed an elevated expression level of *Anp* (*n* = 6–8 animals/group). Expression is corrected for *Hprt* expression and normalized to control. LVEDD, LV end‐diastolic lumen diameter, *Anp*, atrial natriuretic peptide. (c) Quantification of respectively myocardial fibrosis and TUNEL‐positive nuclei to detect apoptotic cells (*n* = 6–8 animals/group). (d) Volcano plots present the overall gene expression of 16‐week and 18‐ to 22‐week‐old *αMHC‐Xpg* ^*c/−*^ compared with 16‐week‐old control (*n* = 3 animals/group). The dashed lines denote the cut‐off values for a differentially expressed gene (DEG; FDR adjusted *P*‐value <0.05 and absolute fold change >1.5). Significantly up‐regulated genes are highlighted in red and significantly down‐regulated genes in green. The non‐DEGs are represented in black. (e) Venn diagram display the total amount of overlapping DEGs in both *αMHC‐Xpg* ^*c/−*^ groups compared with 16‐week‐old control. (f) Heat map of hierarchically clustered overlapping DEGs in 16‐week and 18‐ to 22‐week‐old *αMHC‐Xpg* ^*c/−*^ mice compared with 16‐week‐old control. (g) Hallmark pathways enriched in *αMHC‐Xpg* ^*c/−*^ mice. Dot size represents the number of genes in each involved pathway. Dot colour show −log_10_ (*Q*‐value) in each term enrichment. X‐axis shows the ratio of the genes to all DEGs. (a–c) For the sake of comparison we have repeated 16‐week‐old *αMHC‐Xpg* ^*c/−*^ mice (green bars) and corresponding control (black dotted line) in this figure, both from *Study II*. (d–g) 16‐week‐old *αMHC‐Xpg* ^*c/−*^ mice: *Study II*; 18‐ to 22‐week‐old *αMHC‐Xpg* ^*c/−*^ mice: *Study I*, lifespan study. Data are presented as mean ± SEM. *p < 0.05 vs. corresponding control; †*p* < 0.05 vs. *αMHC‐Xpg* ^*c/−*^ 16 weeks by one‐way ANOVA followed by SNK post hoc testing.

The observed LV dysfunction at 16 weeks of age was not accompanied by LV hypertrophy and pulmonary congestion. However, *αMHC‐Xpg* ^*c/−*^ mice at an advanced age (18–26 weeks) displayed overt congestive LV failure, demonstrated by increases in relative LV weight, left atrial (LA) weight, lung fluid (LF) weight and right ventricular (RV) weight (Figure 5a). Echocardiographic analysis at 22 weeks of age (*Study I*: Lifespan study) revealed a progressively enlarged LV lumen and impairment of fractional shortening compared with 16‐week‐old *αMHC‐Xpg* ^*c/−*^ mice, showing further disease progression, and in addition, the expression level of *Anp* was further elevated (Figure 5b). The LV hypertrophy displayed in advanced age *αMHC‐Xpg* ^*c/−*^ mice was accompanied by significantly increased cardiomyocyte cross‐sectional area compared with 16‐week‐old control mice (data not shown). In addition, collagen fraction was markedly elevated and TUNEL staining revealed a dramatic loss of cells (Figure 5c; Figure S11a). Importantly, at 8 weeks of age, no differences in the level of TUNEL‐positive cells (*αMHC‐Xpg* ^*c/−*^ 1.82 ± 0.32‰, *n* = 6 vs. corresponding control 1.73 ± 0.29‰, *n* = 5; *p* = 0.85) and collagen content (as mentioned previously) were observed between *αMHC‐Xpg* ^*c/−*^ and corresponding control, indicating that apoptosis and fibrosis start at a later age, further underscoring the degenerative nature of the disease process. Cellular stress, such as DNA damage, activates the intrinsic apoptotic pathway (Giam et al., 2008). Indeed, apoptosis in advanced age *αMHC‐Xpg* ^*c/−*^ mice was accompanied by increased expression of phorbol‐12‐myristate‐13‐acetate‐induced protein 1 (*Pmaip1*), Bcl‐ associated X protein (*Bax*), Bcl2‐related ovarian killer protein (*Bok*) and Bcl2‐modifying factor (*Bmf*) (Figure S11b).

### 2.7 TP53 activation and enrichment of apoptosis, DNA damage and impaired DNA repair‐associated pathways in *αMHC‐Xpg* ^*c*^ ^*/−*^ mice

To explore the molecular mechanisms related to DNA damage, messenger RNA sequencing was performed in LV tissues of *αMHC‐Xpg* ^*c/−*^ mice, revealing 384 differentially expressed genes (DEGs; FDR adjusted *p*‐value <0.05 and absolute fold change >1.5) in 16‐week‐old *αMHC‐Xpg* ^*c/−*^ compared with corresponding control. Strikingly, in the advanced age (18–22 weeks) *αMHC‐Xpg* ^*c/−*^, 2833 DEGs were found, of which 342 overlapped with 16‐week‐old *αMHC‐Xpg* ^*c/−*^ DEGs (Figure 5d,e). Analysis of DEGs using ingenuity pathway analysis (IPA) identified the tumour suppressor gene TP53 as one of the top active upstream transcription regulators in 16‐week‐old (overlap *p*‐value 2.34 E‐14; activation z‐score 2.563) and in 18‐ to 22‐week‐old (overlap p‐value 2.06 E‐52; activation z‐score 6.449) *αMHC‐Xpg* ^*c/−*^. TP53 is known to trigger cell‐cycle arrest, apoptosis or DNA repair in response to DNA damage and thereby activates specific genes (Lakin & Jackson, 1999). In advanced age *αMHC‐Xpg* ^*c/−*^, 438 TP53‐target genes were activated (Table S2). This observation in our cardiomyocyte‐restricted DNA repair‐deficient *Xpg* mouse mutant is in excellent agreement with the observation of TP53 activation in another tissue‐restricted DNA repair‐deficient mouse mutant (accompanying manuscript by Henpita et al. (2023)). The overlapping DEGs shown in the heat map were enriched for pathways involved in angiogenesis, apoptosis and inflammation (Figure 5f,g).

Gene ontology (GO) terms enrichment analysis considering all DEGs (*p*‐value <0.05) in *αMHC‐Xpg* ^*c/−*^ at 16 and at 18–22 weeks of age, resulted in seven networks including mitochondrial pathway, transport, signaling, metabolic process, response to stimulus, development and homeostasis (Figure S12). Detailed analysis revealed apoptosis and DNA damage as significant subnetworks (Figure S13). In addition, gene set enrichment analysis based on the biological pathways supplied by KEGG showed enrichment of pathways which are associated with impaired DNA repair, such as Huntington's (adjusted *p*‐value 2.75 E‐17), Parkinson's (adjusted *p*‐value 2.07 E‐24) and Alzheimer's (adjusted *p*‐value 5.82 E‐25) disease in advanced age *αMHC‐Xpg* ^*c/−*^, which are hallmarks of DNA repair disorders (Sepe et al., 2013).

### 2.8 Association of genetic variants in *ERCC1* and *XPG* with cardiac remodelling and dysfunction

To study the effect of genetic variation in the *ERCC1* and *XPG* gene on cardiac function in humans, a genome‐wide association study (GWAS) of LVEDD and fractional shortening was performed in up to 7653 individuals from the population‐based Rotterdam Study (Ikram et al., 2020). The effect estimates for the common genetic variants located in the genes and those 50kB up‐ and downstream were extracted. A total of 26 genetic variants in *ERCC1* were associated with LVEDD (*p*‐value <0.05), of which two single‐nucleotide polymorphisms (SNPs; 19:45967369 and 19:45976718) showed an opposite effect for LVEDD and fractional shortening (Table S3). Conversely, a total of 13 SNPs in *ERCC1* were associated with lower fractional shortening (*p*‐value <0.05), of which six SNPs (19:45877788, 19:45879195, 19:45884546, 19:45888808, 19:45896834 and 19:45914174) showed an opposite effect for fractional shortening and LVEDD (Table S4). For *XPG*, a total of 14 SNPs were associated with LVEDD, of which four SNPs (13:103467184, 13:103457497, 13:103527113 and 13:103548761) showed an opposite effect for LVEDD and fractional shortening (Table S3). Conversely, five SNPs were associated with fractional shortening (*p*‐value <0.05), none of which showed an effect with LVEDD (Table S4). Taken together, these findings reveal genetic variants that may be implicated in the development of heart failure in humans. Of notice, the observation that some of these genetic variants have opposite effects on LVEDD and fractional shortening makes these variants interesting candidates for further study.

## 3 DISCUSSION

Despite major advances in treatment, the exact molecular mechanisms underlying the pathogenesis of heart failure remain incompletely understood hampering a more mechanism‐based approach to effective treatment and prevention. Experimental and clinical evidence indicates that DNA damage—due to oxidative stress (e.g., ischemia–reperfusion injury) or after chemotherapy (Octavia et al., 2012)—is associated with heart failure (Bartunek et al., 2002; Higo et al., 2017; Shukla et al., 2010). However, the precise effects of unrepaired DNA damage, and its role in the pathogenesis of heart failure, are still poorly understood. In the present study, we tested the hypothesis that DNA repair in cardiomyocytes is critical for maintaining normal cardiac function. For this purpose, we employed mice with systemic and cardiomyocyte‐restricted depletion of *Xpg* and *Ercc1*.

The main findings of the present study were that (i) impaired DNA repair resulted in progressive cardiac remodelling and dysfunction, ultimately leading to severe congestive heart failure and premature death. (ii) In both *Xpg* mutants (i.e., *Xpg* ^*−/−*^ mice and *αMHC‐Xpg* ^*c/−*^ mice), systolic LV dysfunction could not be explained by a loss of cardiac myofilament force development. In addition, the lack of loss of peripheral vasodilator function in *αMHC‐Xpg* ^*c/−*^ mice excludes chronic increase in afterload as a mechanism for cardiac remodelling. (iii) Only in *Xpg* ^*−/−*^ mice, diastolic LV dysfunction could be explained by increases in *F*_pas_ and myofilament Ca^2+^ sensitivity, and a reduction in max K_tr_, which could be attributed to decreased protein phosphorylation and a shift in myosin heavy chain isoform composition, respectively. (iv) Myocardial collagen content was progressively increased. (v) Apoptosis was markedly and progressively elevated in both *Xpg* mutants, which was accompanied by increases in reactive oxygen species, expression of pro‐apoptotic genes, activation of TP53 and enrichment of DNA damage‐related pathways and diseases. (vi) In support of the observed cardiac phenotype in mutant mice, several genetic variants in the *ERCC1* and *XPG* gene in human GWAS data were found to be associated with cardiac remodelling and dysfunction. The implications of these findings will be discussed.

### 3.1 Importance of sufficient DNA repair for the maintenance of cardiac function

Unrepaired DNA damage can lead to pronounced cellular dysfunction and promote disease development (Lopez‐Otin et al., 2013). The structure‐specific endonucleases XPG and ERCC1‐XPF, two major players in DNA repair, are responsible for the excision of DNA lesions and are involved in several DNA repair mechanisms (Gregg et al., 2011; Marteijn et al., 2014; Trego et al., 2016). Previously, using cardiomyocyte‐restricted *Ercc1* knockout mice, genome instability in the heart, measured by variant calling in RNA and genomic DNA, was found to be strongly elevated compared with wildtype mice, and several other prematurely ageing mouse models (De Majo et al., 2021). The finding of enhanced mutations is consistent with the repair deficiency. However, small‐scale mutations are now being recognized not to be a major driver of ageing (Robinson et al., 2021), in line with the growing notion that DNA damage rather than mutations is relevant for ageing‐associated functional decline in post‐mitotic organs and tissues (Schumacher et al., 2021) and consistent with our finding of DNA damage‐driven transcription stress, which we also found to be enhanced as apparent from gene expression data sets of ageing in the heart (Gyenis, Chang, et al., Nat. Genetics in press). In the present study, both systemic and cardiomyocyte‐restricted loss of *Xpg* and *Ercc1* (i.e., *Xpg* ^*−/−*^, *αMHC‐Xpg* ^*c/−*^, *Ercc1* ^*Δ/−*^ and *αMHC‐Ercc1* ^*c/−*^ mice) resulted in significant cardiac dysfunction. In conjunction with recent observations no or only minimal cardiac dysfunction in endothelium‐restricted (Bautista‐Nino et al., 2020) and smooth‐muscle‐restricted (Ataei Ataabadi et al., 2021) *Ercc1* mutant mice, respectively, the present findings imply a critical role for cardiomyocyte DNA repair in maintaining cardiac function.

Importantly, we observed normal cardiac function in all DNA repair‐deficient mutants at 4 weeks of age, after which all mutants gradually developed contractile dysfunction. These observations indicate that loss of DNA repair induces a degenerative rather than a developmental process in the heart. These results are consistent with observations in mice in which deficient DNA repair is induced by depletion of *Xrcc1*, an essential protein for single‐strand break repair. In pressure overload‐induced heart failure, accumulation of single‐strand breaks was associated with more activated DNA damage response and deteriorated cardiac dysfunction in *Xrcc1* deficient mutants (Higo et al., 2017).

In the present study, cardiac dysfunction in both systemic and cardiomyocyte‐restricted mutants was associated with a markedly reduced lifespan. Interestingly, cardiomyocyte‐restricted loss of *Xpg* and *Ercc1* resulted in a slightly longer lifespan compared with mice with systemic loss of *Xpg* and *Ercc1*, suggesting that the heart is not lifespan limiting in the systemic mutants. This is also supported by the relatively milder degree of cardiac dysfunction in the systemic (fractional shortening ~30%) versus the cardiomyocyte‐restricted (~10%) mutants just prior to death. Conversely, the cardiomyocyte‐restricted mutants displayed severe signs of congestive heart failure in the final week, suggesting that mortality in these mice was related to the severe loss of cardiac function. It has been shown that survival rates of DNA repair‐deficient mouse models depend on the extent to which DNA repair mechanisms are affected, with *Ercc1* mutants showing an even more severe phenotype than *Xpg* mutants (Vermeij, Dollé, et al., 2016). Indeed, we observed a slightly worse survival in the cardiomyocyte‐restricted *Ercc1* mutants compared with the *Xpg* mutants. From the present study, we cannot determine the exact cause of death, that is progressive pump failure or fatal ventricular arrhythmias, in the cardiomyocyte‐restricted mutants. However, the observation that the surface ECG analysis did not show any sign of ventricular arrhythmias, together with the severe signs of congestive heart failure, including dyspnoea, pulmonary oedema and ascites, suggests that the cause of death most likely was progressive pump failure.

### 3.2 Mechanism of cardiac dysfunction

The mechanisms underlying the observed cardiac dysfunction in the *Xpg* and *Ercc1* mutants could be several‐fold, including altered cardiomyocyte contractile properties, enhanced extracellular matrix deposition and increased levels of senescence and myocardial cell loss.

DNA repair‐deficient mutants demonstrated severe systolic LV dysfunction, which could not be explained by a loss of maximum force development of individual cardiomyocytes, as F_max_ was maintained in both *Xpg* mutants. We also observed diastolic LV dysfunction in *Xpg* mutants, which could be explained, at least in the systemic *Xpg* mutants, by significant elevations in F_pas_, myofilament Ca^2+^ sensitivity, and a decrease in max K_tr_. The increased Ca^2+^ sensitivity was likely due to the lower PKA‐mediated cTnI phosphorylation, while reduced max K_tr_ is explained by the shift towards the β‐MHC isoform. The maintained force development, the increased Ca^2+^ sensitivity and lower cTnI phosphorylation are in excellent agreement with the observed alterations in cardiomyocyte function in patients with end‐stage dilated cardiomyopathy (DCM; Hamdani et al., 2008, 2010).

We also found evidence of extracellular matrix remodelling, as we observed a 50%–80% increase in LV myocardial collagen content at 16 weeks of age, with an additional 240% increase by the end of the cardiomyocyte‐restricted *Xpg* mutants' lifespan. These observations are similar to the elevated collagen volume fraction in ventricular biopsies of end‐stage DCM patients (Hamdani et al., 2010) and may have contributed to the progressive deterioration of cardiac function.

DNA damage can interfere with the vital process of transcription or induce replication arrest, which can trigger cellular senescence or cell death (Hoeijmakers, 2009). Several studies with different DNA repair‐deficient mouse mutants concerning other organ systems showed elevated levels of senescence markers, including increased expression of *p21* and *p16*, and increased senescence‐associated β galactosidase activity (Barnhoorn et al., 2014; Vermeij, Dollé, et al., 2016). In line with those previous studies, we observed increased expression of *p21* and *p16* in the liver of 16‐week‐old systemic *Xpg* mutants. Interestingly, expression of *p21* and *p16* was not elevated in the *Xpg* mutant heart at 16 weeks of age, which could be interpreted to suggest that differentiated cardiomyocytes with depleted *Xpg* appear may be highly sensitive to DNA damage, responding with early apoptotic cell death. Indeed, examination of apoptosis showed significantly elevated levels of apoptotic cell death in both *Xpg* mutants, already at 16 weeks, which was markedly (fivefold) further increased in the cardiomyocyte‐restricted *Xpg* mutants at the end of their lifespan. These observations support the concept that the transition from cardiac dysfunction to overt heart failure is due to increased apoptosis (Li et al., 1997; Marin‐Garcia, 2016).

There is evidence that DNA damage can drive apoptosis in cardiomyocytes (Higo et al., 2017; Shukla et al., 2011). The mechanisms underlying DNA damage‐induced apoptosis may involve activation of TP53, a key regulator of apoptosis. Thus, in the accompanying manuscript by Henpita et al. (2023), unrepaired DNA damage in differentiated cardiomyocytes resulted in activation of p53, while genetic depletion of p53 attenuated the level of apoptosis. In line with their observations, the dramatic loss of cardiomyocytes at the end of the cardiomyocyte‐restricted *Xpg* mutants' lifespan was associated with activation of TP53 and elevated expression of the pro‐apoptotic genes *Bax*, *Pmaip1*, *Bok* and *Bmf*. Oxidative stress has been implicated in the pathogenesis of heart failure. In the failing human heart, due to dilated cardiomyopathy and acute myocardial infarction, elevated levels of the oxidative DNA damage marker 8‐oxo‐7,8‐dihydro‐2′‐deoxyguanosine and DNA repair enzymes were detected in serum and in cardiomyocytes (Bartunek et al., 2002; Kono et al., 2006). Interestingly, both *Xpg* mutants showed NOX‐dependent increases in superoxide production, which may have contributed to apoptosis, possibly via activation of TP53, and the progressive deterioration of cardiac pump function in the DNA repair‐deficient mutants.

Gene set enrichment analysis showed that the subnetworks of apoptosis and DNA damage were enriched. The NER pathway plays a critical role in neurodegenerative diseases, such as Alzheimer's, Parkinson's and Huntington's disease. Impaired DNA repair, due to defects in the NER pathway, causes severe neurodevelopmental abnormalities (Sepe et al., 2013). Identification of diseases related to the differentially expressed genes in our dataset revealed that cardiomyocyte‐restricted loss of *Xpg*, affected genes that are involved in these DNA damage‐related neurodegenerative diseases, consistent with the concept that DNA damage is present in our *Xpg*‐mutant mice.

### 3.3 Association of genetic variants in ERCC1 and XPG with cardiac remodelling and dysfunction

To study the effect of genetic variation in the *ERCC1* and *XPG* gene on cardiac remodelling and dysfunction in humans, a genome‐wide association study of LVEDD and fractional shortening was performed in the Rotterdam Study. These results revealed that some of the genetic variants in *ERCC1* and *XPG* genes were associated with an increase in LVEDD and a decrease in fractional shortening (corresponding with the cardiac phenotype in our mutant mice), suggesting that these variants might be implicated in the development of heart failure in humans.

### 3.4 Conclusions

The present study, in conjunction with the accompanying study by Henpita et al. (2023), demonstrates that unrepaired endogenously generated DNA damage in differentiated cardiomyocytes drives early onset of cardiac failure. Since *Xpg* and *Ercc1* are both involved in NER, TCR and pathways of DSB repair, these genome stability mechanisms protect against cardiac failure. Importantly, even in the presence of these potent repair mechanisms, DNA damage inevitably accumulates over time even in normal cells, as some types of DNA damage are not recognized by global genome repair systems, while others are irreparable (Hoeijmakers, 2009). Moreover, age‐related decline in repair also occurs (Moriwaki & Takahashi, 2008; Vermeij, Dollé, et al., 2016). Hence, time‐dependent accumulation of DNA lesions may well contribute to the aetiology of heart failure, implicating DNA damage as a novel therapeutic target. Moreover, our work highlights systemic and cardiomyocyte‐restricted DNA repair‐deficient mutants as bona fide models of heart failure well suited for identifying novel therapeutic interventions.

## 4 EXPERIMENTAL PROCEDURES

For detailed experimental procedures, see Extended Methods in Appendix S2.

### 4.1 Animals

Generation and characterization of *Xpg* ^*−/−*^, *Ercc1* ^*Δ/−*^ and *Ercc1* ^*−/−*^ mice have been described previously (Barnhoorn et al., 2014; Weeda et al., 1997). Briefly, *Xpg* ^*−/−*^ mice were obtained by crossing C57BL/6J *Xpg* ^*+/−*^ with FVB/N *Xpg* ^*+/−*^ animals, *Ercc1* ^*Δ/−*^ mice by crossing FVB/N *Ercc1* ^*Δ/+*^ with C57BL/6J *Ercc1* ^*+/−*^ and *Ercc1* ^*−/−*^ mice by crossing C57BL/6J *Ercc1* ^*+/−*^ with FVB/N *Ercc1* ^*+/−*^ animals, resulting in experimental animals in C57BL6/FVB F1 hybrid background. In order to study cardiomyocyte‐restricted effects of DNA repair deficiency on cardiac geometry, function and structure, mice with cardiomyocyte‐restricted inactivation of *Xpg* and *Ercc1* genes were generated (Figure S2a,b). The generation of Cre‐inducible *Xpg* and *Ercc1* knockout alleles has been described previously (Barnhoorn et al., 2014; Doig et al., 2006). To achieve cardiomyocyte‐restricted *Xpg* and *Ercc1* gene inactivation, a transgenic line with Cre recombinase under control of the alpha‐myosin heavy chain (αMHC) promotor was used (Agah et al., 1997). *αMHC‐Cre* ^*+/−*^ female mice were crossed with *Xpg* ^*+/−*^ male mice or with *Ercc1* ^*+/−*^ male mice to generate, respectively, *Xpg* ^*+/−*^ *αMHC‐Cre* ^*+/−*^ and *Ercc1* ^*+/−*^ *αMHC‐Cre* ^*+/−*^ mice in a C57BL/6J background. The obtained females were subsequently crossed with *Xpg* ^*fl/fl*^ and *Ercc1* ^*fl/fl*^ male mice in a FVB/N background to yield *Xpg* ^*fl/−*^ *αMHC‐Cre* ^*+*^ and *Ercc1* ^*fl/−*^ *αMHC‐Cre* ^*+*^ mice in a C57BL6/FVB F1 hybrid background. *Xpg* ^*fl/−*^ *αMHC‐Cre* ^*+*^ and *Ercc1* ^*fl/−*^ *αMHC‐Cre* ^*+*^ mice, respectively, were heterozygous for *Xpg* and *Ercc1* in all cell types, except for cardiomyocytes, which were homozygous for *Xpg* and *Ercc1*, after Cre‐mediated excision of the floxed allele. These mice are referred to as *αMHC‐Xpg* ^*c/−*^ and *αMHC‐Ercc1* ^*c/−*^, respectively, throughout this manuscript. *Xpg* ^*fl/+*^ *αMHC‐Cre* ^*+*^ and *Ercc1* ^*fl/+*^ *αMHC‐Cre* ^*+*^ (referred to as *αMHC‐Xpg* Ctrl and *αMHC‐Ercc1* Ctrl) were used as controls. These controls were wildtype in all cell types, except for cardiomyocytes, which were heterozygous. All animals described in this study were born at a predicted Mendelian frequency and have the same genetic C57BL6/FVB F1 hybrid background to minimize background‐specific effects. All animals were bred and maintained with ad libitum access to water and AIN93G synthetic pellets (Research Diet Services B.V., Wijk bij Duurstede, The Netherlands; gross energy content 4.9 kcal/g dry mass, digestible energy 3.97 kcal/g). A total of 342 mice of either sex entered the study (randomized). Each experiment included males and females, except the mRNA sequencing of LV tissue, which was performed only in males. No animals were excluded. Experiments were performed in accordance with the Council of Europe Convention (ETS123) and the Directive (2010/63/EU) for the protection of vertebrate animals used for experimental and other scientific purposes, and with approval of the Animal Care Committee of Erasmus MC, University Medical Center Rotterdam.

### 4.2 Experimental design

Two study protocols were performed. Study I consisted of a lifespan study, based on which we designed Study II, in which animals were studied in great detail and sacrificed after a pre‐determined follow‐up period.

#### 4.2.1 Study I

Lifespan studies were performed to characterize body growth, as well as global cardiac geometry and function in the various DNA repair‐deficient mutants described above. For this purpose, animals were weighed and subsequently underwent transthoracic echocardiography, every 2‐4 weeks, until death (DNA repair‐deficient mutants) or 30 weeks of age (control mice).

#### 4.2.2 Study II

To study cardiac geometry, function and structure in more detail, *Xpg* ^*−/−*^, *αMHC‐Xpg* ^*c/−*^, *Ercc1* ^*Δ/−*^, *αMHC‐Ercc1* ^*c/−*^ mice and corresponding control littermates were sacrificed at the age of 16 weeks. Since *αMHC‐Ercc1* ^*c/−*^ mice are deficient for *Ercc1* in their cardiomyocytes, we added an additional group of *Ercc1* ^*−/−*^ animals to Study II. However, as these animals have a much shorter lifespan (~8–10 weeks), we studied this additional group at 8 weeks of age.

#### 4.2.3 Statistical analysis animal studies

Data from study I (lifespan study) were compared using mixed linear model–repeated measures (SPSS Statistics 21.0). Data from study II were compared using one‐way or two‐way ANOVA, followed by Student–Newman–Keuls (SNK) post hoc testing when appropriate (SigmaPlot 11.0). Comparison of variables between two groups at a single time point was performed by unpaired Student's *t*‐test (SigmaPlot 11.0). Vascular function curves (Figure S5) were compared with general linear model for repeated measures (GLM‐RM), assuming sphericity (SPSS Statistics 21.0). Since male and female mice demonstrated very similar LV responses to DNA repair deficiency (Figure S14), males and females were pooled. Statistical significance was accepted when *p* < 0.05 (two‐tailed). All data are presented as mean ± SEM.

### 4.3 Human genetic studies

#### 4.3.1 Study cohort

In accordance with the observed phenotype in *Xpg* and in *Ercc1* mutants, the association of common single‐nucleotide polymorphisms (SNPs) in both genes with enlarged LV lumen diameter and deteriorated fractional shortening were investigated. To this end, a genome‐wide association study (GWAS) of these two measures was performed in the population‐based Rotterdam Study (RS). The RS is a prospective cohort study, initially comprising 7983 individuals (78% of 10,215 invitees), 55 years of age or over, living in the well‐defined Ommoord district in the city of Rotterdam, The Netherlands (RS‐I, recruitment 1990–1993). The RS aims to examine determinants of disease and health in older subjects focussing on neurogeriatric, cardiovascular, bone and eye diseases (Ikram et al., 2020). The RS has been extended several times. In 2000, RS was extended to include individuals who had become 55 years of age or moved into the study district since the start of the study (RS‐II, including 3011 participants out of 4472 invitees). In 2006, the cohort was further extended to include subjects aged 45 years or over (RS‐III, 3932 subjects out of 6057 invitees). By the end of 2008, RS comprised 14,926 subjects, aged 45 years or over, with an overall response figure of 72.0% (14,926 out of 20,744). Participants of the RS are being revisited at the research centre every 3–4 years. Current analyses included participants from the fourth examination of the original cohort (RS‐I‐4), the second examination of the second cohort (RS‐II‐2) and the baseline examination of the third cohort (RS‐III‐1) for whom both genetic data and echocardiographic assessment were available. The RS has been approved by the Medical Ethics Committee of the Erasmus Medical Center and by the Ministry of Health, Welfare and Sport of the Netherlands, implementing the Wet Bevolkingsonderzoek: ERGO (Population Studies Act: Rotterdam Study). All participants provided written informed consent to participate in the study and to obtain information from their treating physicians.

#### 4.3.2 Statistical analysis

Genome‐wide association was done among 7653 individuals for LVEDD (RS1: *N* = 2926, RS2: *N* = 2206, RS3: *N* = 2521) and a total of 7499 for fractional shortening (RS1: *N* = 2807, RS2: *N* = 2181, RS3: *N* = 2511). Genetic variants were tested for association with markers of cardiac function by linear regression adjusted for age, sex and four principal components (Rvtests). The study was imputed using the HRC reference v1.1. panel. Quality control of the data excluded genetic variants with a minor allele frequency (MAF) of <0.05 and imputation quality of <0.3. Summary‐level results for each of the three RS cohorts were meta‐analysed jointly with METAL (released on March 25, 2011) using a fixed‐effects model with an inverse‐variance weighted approach, correcting for genomic control. We extracted information for the genetic variants that were located in the *ERCC1* and *XPG* (plus 50kB up and downstream of the gene) gene region from the genome‐wide association results to assess their association with LVEDD and fractional shortening. In view of the hypothesis‐driven nature of these analyses, we considered a *p*‐value <0.05 as statistically significant.

## AUTHOR CONTRIBUTIONS

M.B. provided experimental design, generated cardiomyocyte‐specific mutants, performed the majority of the experiments, analysed data and wrote the manuscript. M.L.H. provided technical assistance in animal experiments and assistance in figure design. J.C., J.P., L.M., L.J.W. and M.S. provided experimental design and performed gene expression analysis. B.S.T. and Y.R. performed and analysed in vivo μCT‐FMT and ex vivo FMT imaging. M.G.J.K. provided technical assistance. Y.O. performed and analysed lucigenin experiments. E.D.D. and J.V. designed myofilament function experiments. L.A.B. performed and analysed quantitative RT‐PCR experiments. R.M.C.B. and S.B. generated systemic mutant mice. P.K.B. and A.J.M.R. quantified vascular function. I.K.P. performed histology and immunohistochemistry and analysed images. R.W. and C.A.R. analysed ECG data. B.S.T. and J.E. designed the in vivo μCT‐FMT and ex vivo FMT imaging experiments. M.M.B. and B.A. performed genome‐wide association studies in the Rotterdam Study. M.L.H., J.C., M.M.B., E.D.D., B.A., M.G., C.K., A.H.J.D., I.P., C.A.R., A.J.M.R., M.K., J.E. and J.V. critically revised manuscript. J.H.J.H. and D.J.D. conceived the study, designed the study and wrote the manuscript. D.J.D. coordinated the study.

## FUNDING INFORMATION

This work was supported by the Dutch CardioVascular Alliance: An initiative with financial support of the Dutch Heart Foundation [Grants 2017B018‐ARENA‐PRIME (to L.J.W., J.V. and D.J.D.), 2018B030‐PREDICT2 (to C.A.R.) and 2021B008‐RECONNEXT (to D.J.D.)], Lijf en leven [Grant DIVERS (to J.E.)], National Institute of Health (NIH)/National Institute of Ageing (NIA) [Grant PO1 AG017242 (to J.H.J.H. and J.P.)], European Research Council Advanced Grants [Grants DamAge (to J.H.J.H.) and Dam2Age (to J.H.J.H.)], Dutch Cancer Society [Grant ONCODE (to J.H.J.H.)], Memorabel and Chembridge (ZonMW; to J.H.J.H. and J.P), BBoL (NWO‐ENW; to J.H.J.H.) and the Deutsche Forschungsgemeinschaft [Grant SFB 829 (to J.H.J.H.).

## CONFLICT OF INTEREST

The authors declare no conflict of interest.

## Supporting information


Appendix S1.

Appendix S2.

## Data Availability

The data that support the findings of this study are available on reasonable request to the corresponding author.
