# Supplementary material for: DNA repair in cardiomyocytes is critical for maintaining cardiac function in mice
Source: Aging Cell. 2023 Feb 8;22(3):e13768. doi: 10.1111/acel.13768 (PMC10014058; doi:10.1111/acel.13768)
Supplement: Supplementary file 1 — Appendix S1. [file ACEL-22-e13768-s002.docx]

***Aging Cell***

**Supporting information**

**DNA repair in cardiomyocytes is critical for maintaining cardiac function in mice**

**Running title:** DNA repair and cardiac function

Martine de Boer^1^, Maaike te Lintel Hekkert^1^, Jiang Chang^2^, Bibi S. van Thiel^2,3,4^, Leonie Martens^5^, Maxime M. Bos^6^, Marion G.J. de Kleijnen^1^, Yanto Ridwan^2,7^, Yanti Octavia^1^, Elza D. van Deel^8^, Lau A. Blonden^1^, Renata M.C. Brandt^2^, Sander Barnhoorn^2^, Paula K. Bautista-Niño^4,9^, Ilona Krabbendam-Peters^1^, Rianne Wolswinkel^10^, Banafsheh Arshi^6^, Mohsen Ghanbari^6^, Christian Kupatt^11,12,13^, Leon J. de Windt^14^, A.H. Jan Danser^4^, Ingrid van der Pluijm^2,3^, Carol Ann Remme^10^, Monika Stoll^5,15^, Joris Pothof^2^, Anton J.M. Roks^4^, Maryam Kavousi^6^, Jeroen Essers^2,3,7^, Jolanda van der Velden^8,16^, Jan H.J. Hoeijmakers^2,17,18^, Dirk J. Duncker^1*^

*****Corresponding author: Dirk J. Duncker MD, PhD; Div. Experimental Cardiology, Dept. Cardiology, Ee-2351; Erasmus University Medical Center; PO Box 2040, 3000 CA Rotterdam, The Netherlands

Tel: +31 10 7038066; Fax: +31 10 7044769; E-mail: d.duncker@erasmusmc.nl

**
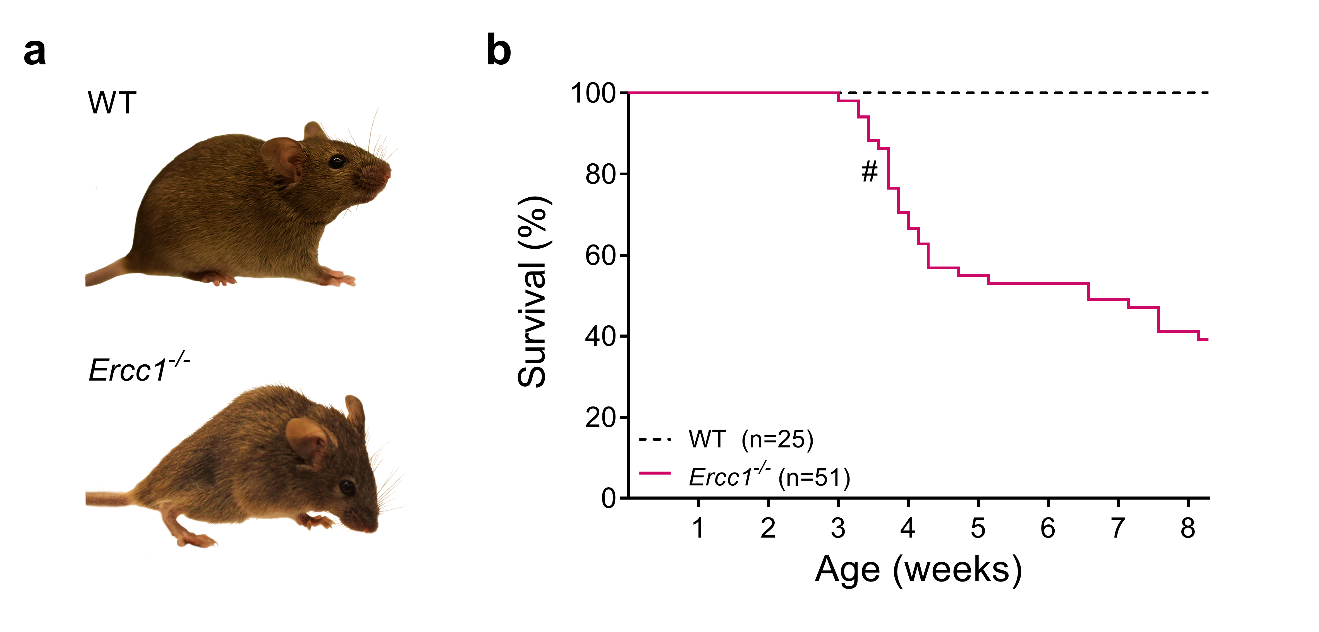
**

**Figure S1** Severe growth retardation and strongly reduced lifespan in *Ercc1^-/-^* mice. (*Study I*, lifespan study) (**a**) Representative pictures of 8-week-old *Ercc1^-/-^* mouse and control littermate. (**b**) Lifespan of *Ercc1^-/-^* mice and corresponding control. The number of animals is indicated in the graph. # weaning at age 3-4 weeks. Mice were sacrificed at an age of 8 weeks.

**
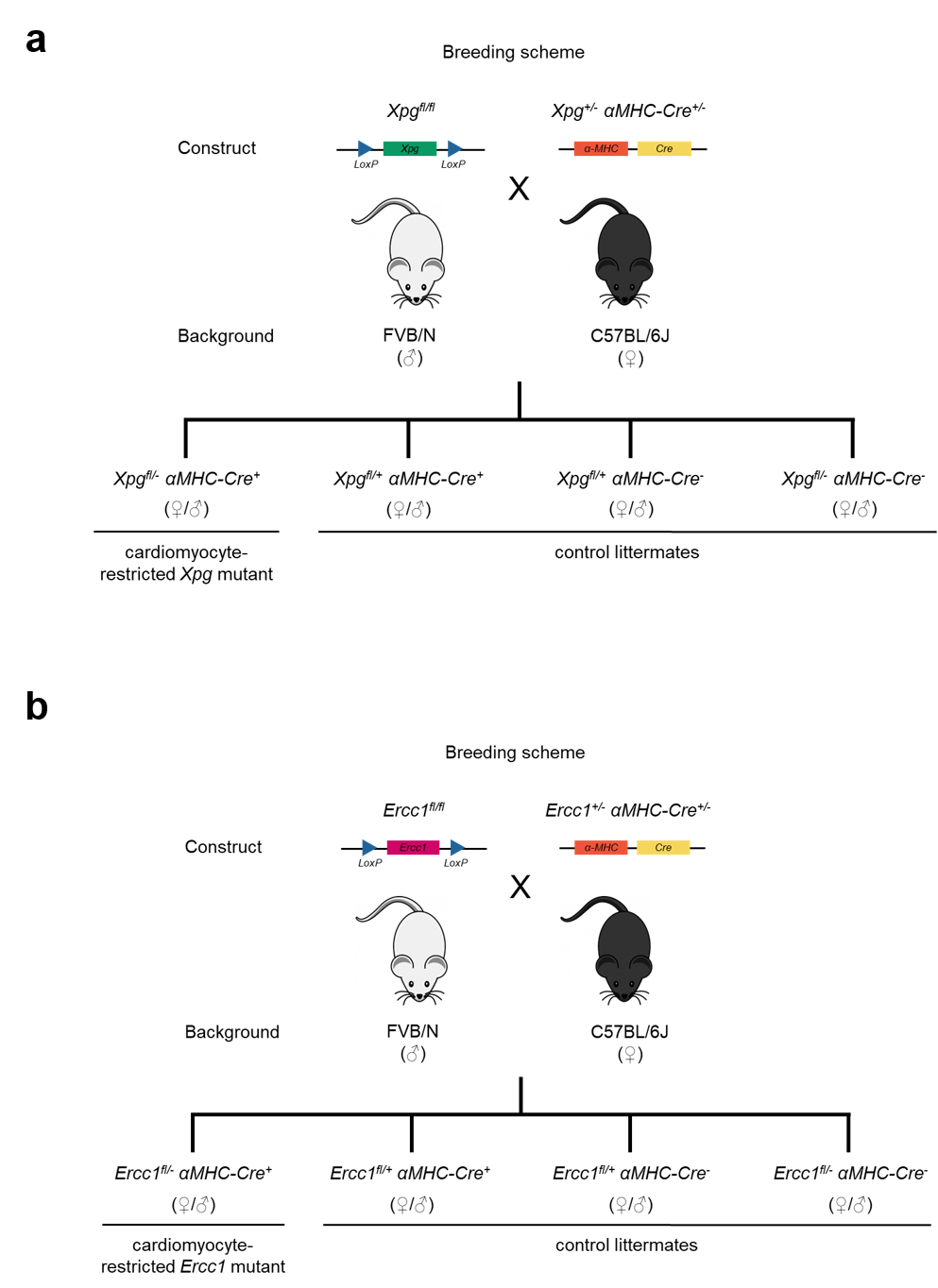
**

**Figure S2** Breeding scheme for the generation of cardiomyocyte-restricted *Xpg* and *Ercc1* mutants and corresponding control littermates. (**a**) *Xpg^fl/fl^* male mice in a FVB/N background were crossed with *Xpg^+/-^* *αMHC-Cre^+/-^* female mice in a C57BL/6J background to obtain *Xpg^fl/-^ αMHC-Cre^+^* mice in a C57BL6/FVB F1 hybrid background. (**b**) *Ercc1^fl/fl^* male mice in a FVB/N background were crossed with *Ercc1^+/-^* *αMHC-Cre^+/-^* female mice in a C57BL/6J background to obtain *Ercc1^fl/-^ αMHC-Cre^+^* mice in a C57BL6/FVB F1 hybrid background. *Xpg^fl/-^ αMHC-Cre^+^* and *Ercc1^fl/-^ αMHC-Cre^+^* mice are heterozygous for *Xpg* and *Ercc1* (respectively) in all cell types, except for cardiomyocytes, which were homozygous for *Xpg* and *Ercc1*, after Cre-mediated excision of the floxed allele. These cardiomyocyte-restricted *Xpg* and *Ercc1* mutants are referred to as *αMHC-Xpg^c/-^* and *αMHC-Ercc1^c/-^* respectively throughout this manuscript. *Xpg^fl/+^ αMHC-Cre^+^* and *Ercc1^fl/+^ αMHC-Cre^+^* (referred to as *αMHC-Xpg* Ctrl and *αMHC-Ercc1* Ctrl throughout this manuscript) were used as controls.

**
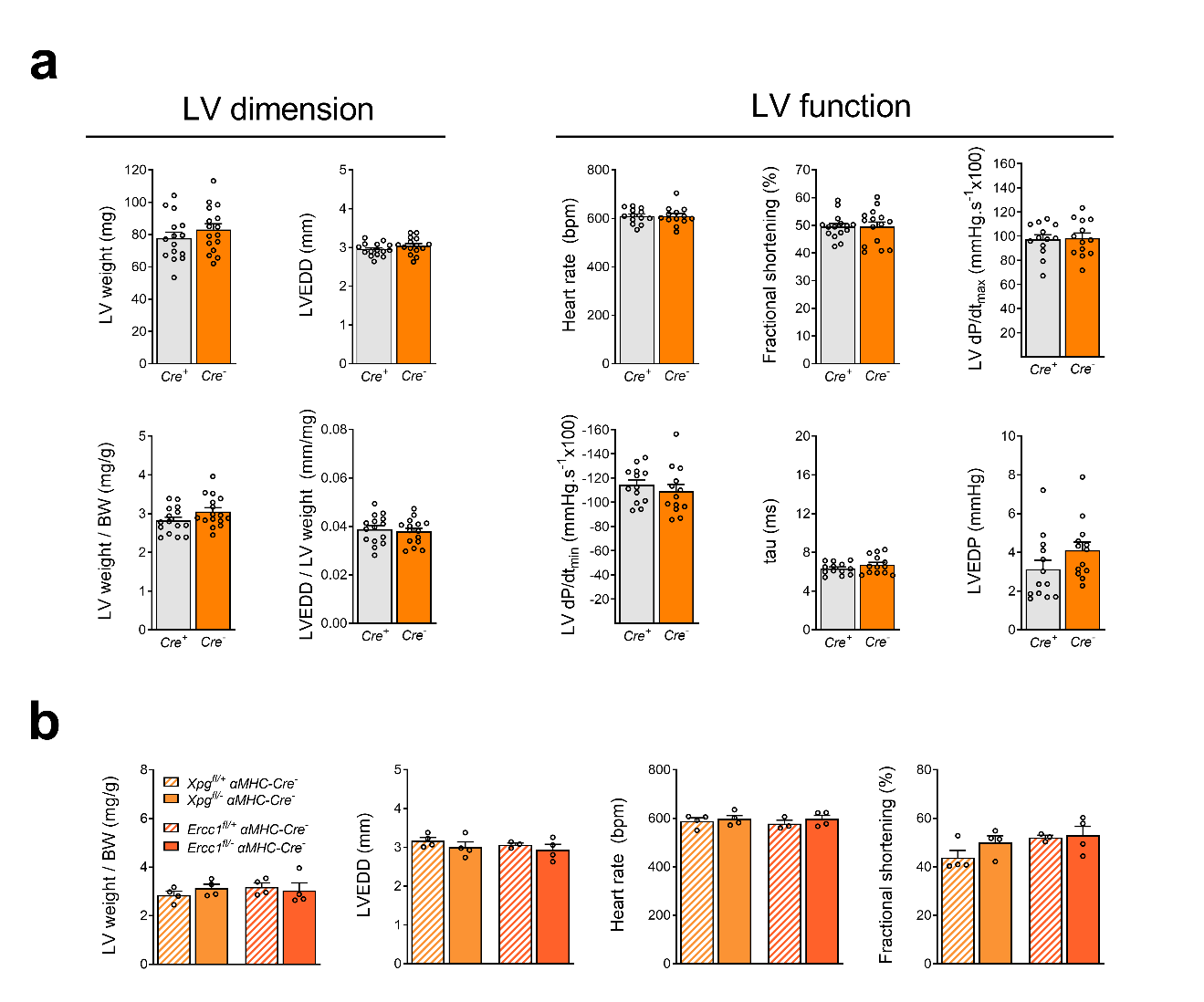
**

**Figure S3** Functional data of the different control littermates. (*Study II*) (**a**) Lack of significant differences in LV remodeling and LV function in 16 weeks old control mice heterozygous for the floxed allele and the *Cre* transgene (*Cre^+^*) compared with control mice heterozygous for the floxed allele only (*Cre^-^*) (n=13-16 animals/group). LV weight, left ventricular weight; LVEDD, LV end-diastolic lumen diameter; BW, body weight; LVdP/dt_max_, maximum rate of rise of LV pressure; LVdP/dt_min_, maximum rate of fall of LV pressure; tau, relaxation time constant; LVEDP, LV end-diastolic pressure. (**b**) Lack of significant differences in LV remodeling and global LV function in 16 weeks old control mice heterozygous for *Xpg* or *Ercc1* in all cell types compared with control mice wildtype in all cell types (n=3-4 animals/group). LV weight, left ventricular weight; LVEDD, LV end-diastolic lumen diameter. Data are presented as mean±SEM. No significant differences were observed by two-tailed, unpaired Student’s *t* test (a) and two-way ANOVA followed by SNK post-hoc testing (b).

**
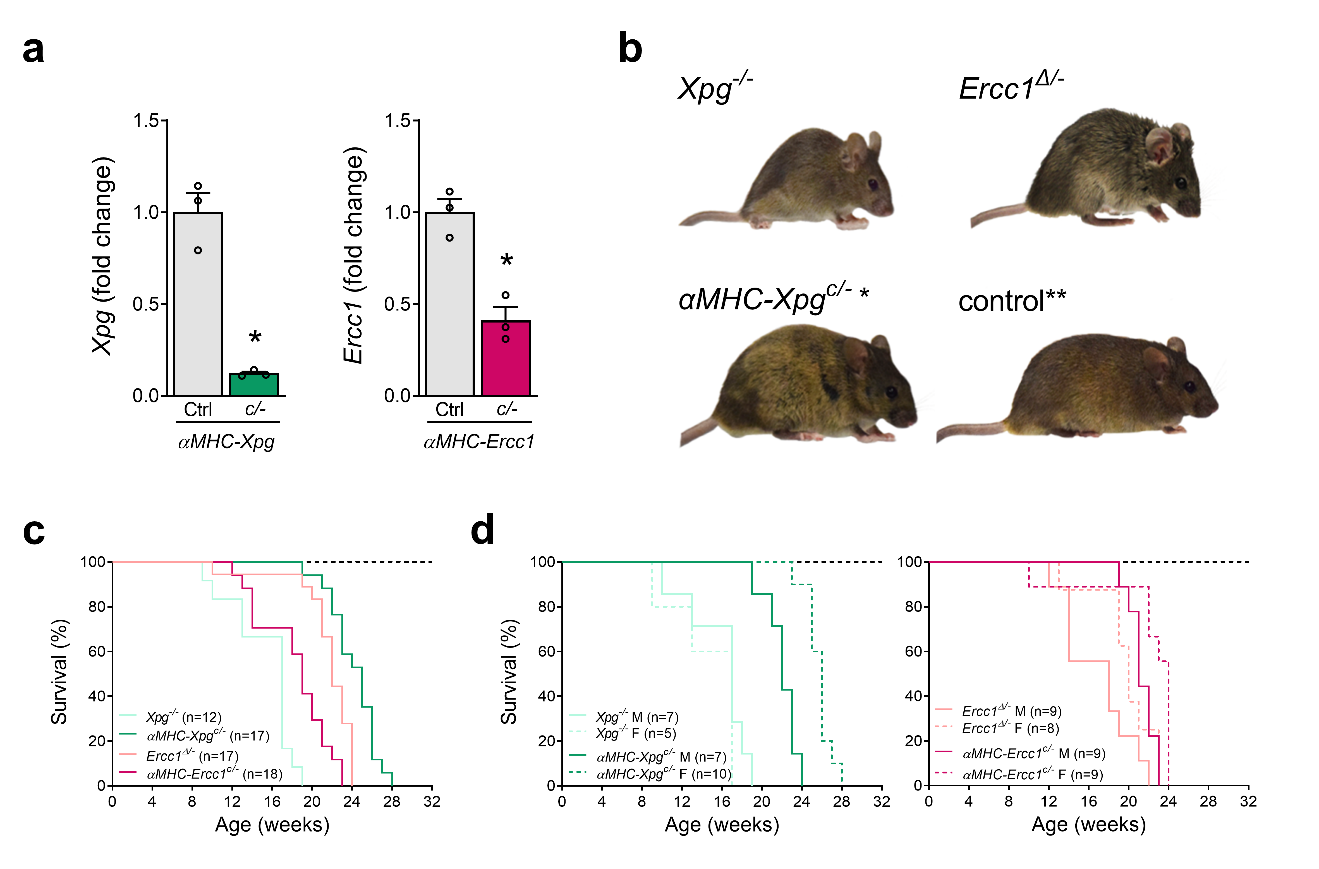
**

**Figure S4** Strongly reduced lifespan in systemic, as well as in cardiomyocyte-restricted *Xpg* and *Ercc1* mutants. Additionally, systemic *Xpg* and *Ercc1* mutants showed severe growth retardation. (*Study I*, lifespan study) (**a**) Quantitative real-time PCR analysis showed attenuated mRNA levels of *Xpg* and *Ercc1* in the *αMHC-Xpg^c/-^* and *αMHC-Ercc1^c/-^* heart, respectively, compared with corresponding control hearts (n=3 animals/group). All values are corrected for *Hprt* and *Gapdh* expression and normalized to corresponding control. Data are presented as mean±SEM. (**b**) Representative pictures of the different DNA repair deficient mutants at age 16 weeks; all males; all in the same C57BL6/FVB F1 hybrid background. **αMHC-Xpg^c/-^* and *αMHC-Ercc1^c/-^* mice; **all corresponding control littermates (**c**) Lifespan of *Xpg^-/-^*, *αMHC-Xpg^c/-^*, *Ercc1^Δ/-^* and *αMHC-Ercc1^c/-^* mice all in the same C57BL6/FVB F1 hybrid background. Black dotted line indicate the control littermates for each mutant, in which there was no loss of animals observed. The number of animals is indicated in the graph. (**d**) Both male and female mice showed shortened lifespan in the different DNA repair deficient mutants. Black dotted line in each graph indicate the control littermates for both sexes, in which there was no loss of animals observed. *P<0.05 vs. corresponding control by two-tailed, unpaired Student’s *t* test.

**
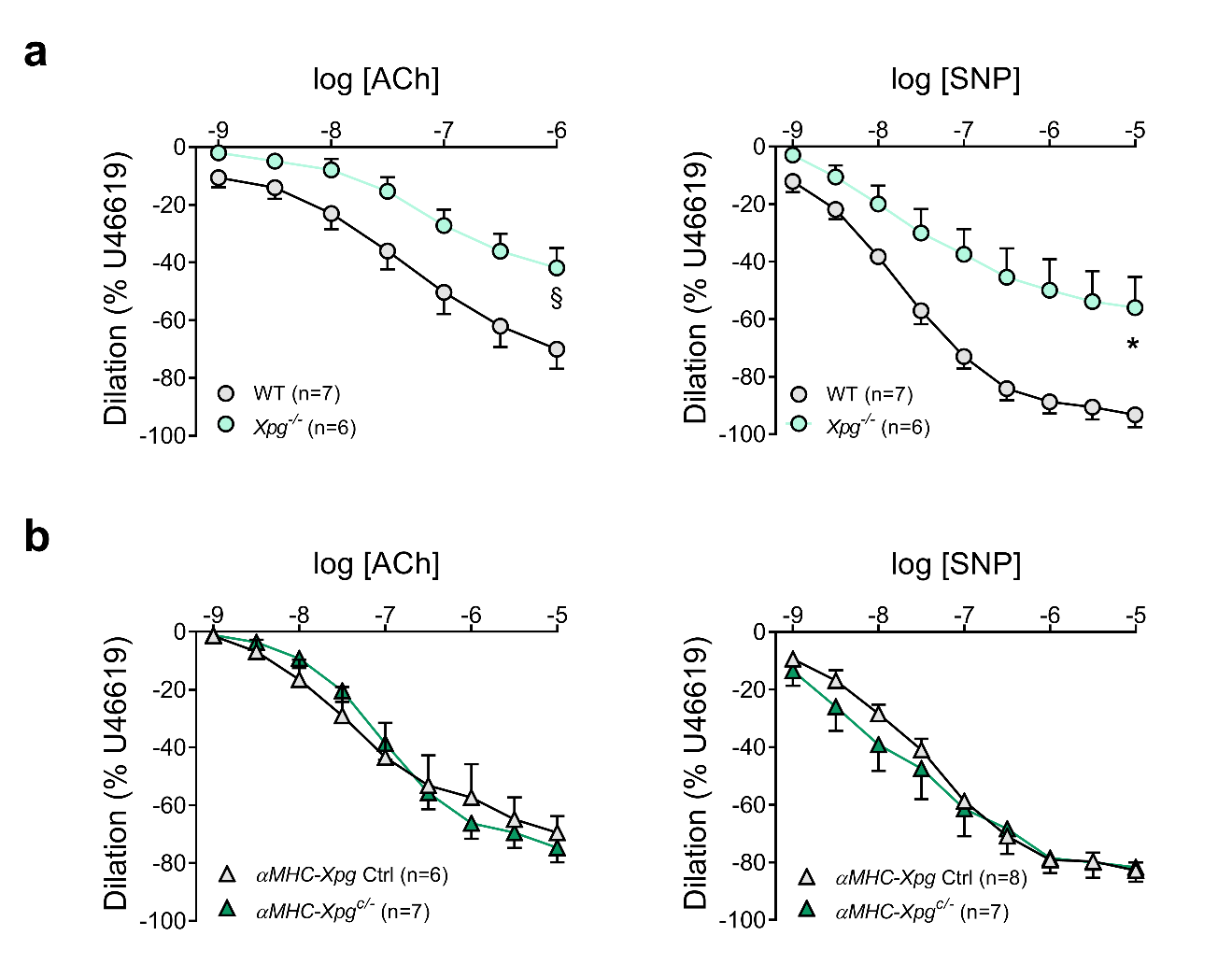
**

**Figure S5** *Ex vivo* vascular dysfunction in *Xpg^-/-^* mice at age 16 weeks. (*Study II*)

Vasodilatation to acetylcholine (ACh) and sodium nitroprusside (SNP) in isolated aortic segments of (**a**) 16-week-old *Xpg^-/-^*, (**b**) *αMHC-Xpg^c/-^* mice and corresponding control. Vasodilator responses were expressed as percentage of the preconstriction to U46619. The number of animals is indicated in the graph. Data are presented as mean±SEM. *P<0.05, § P=0.06 vs. corresponding control using general linear model for repeated measures (GLM-RM; sphericity assumed).

**
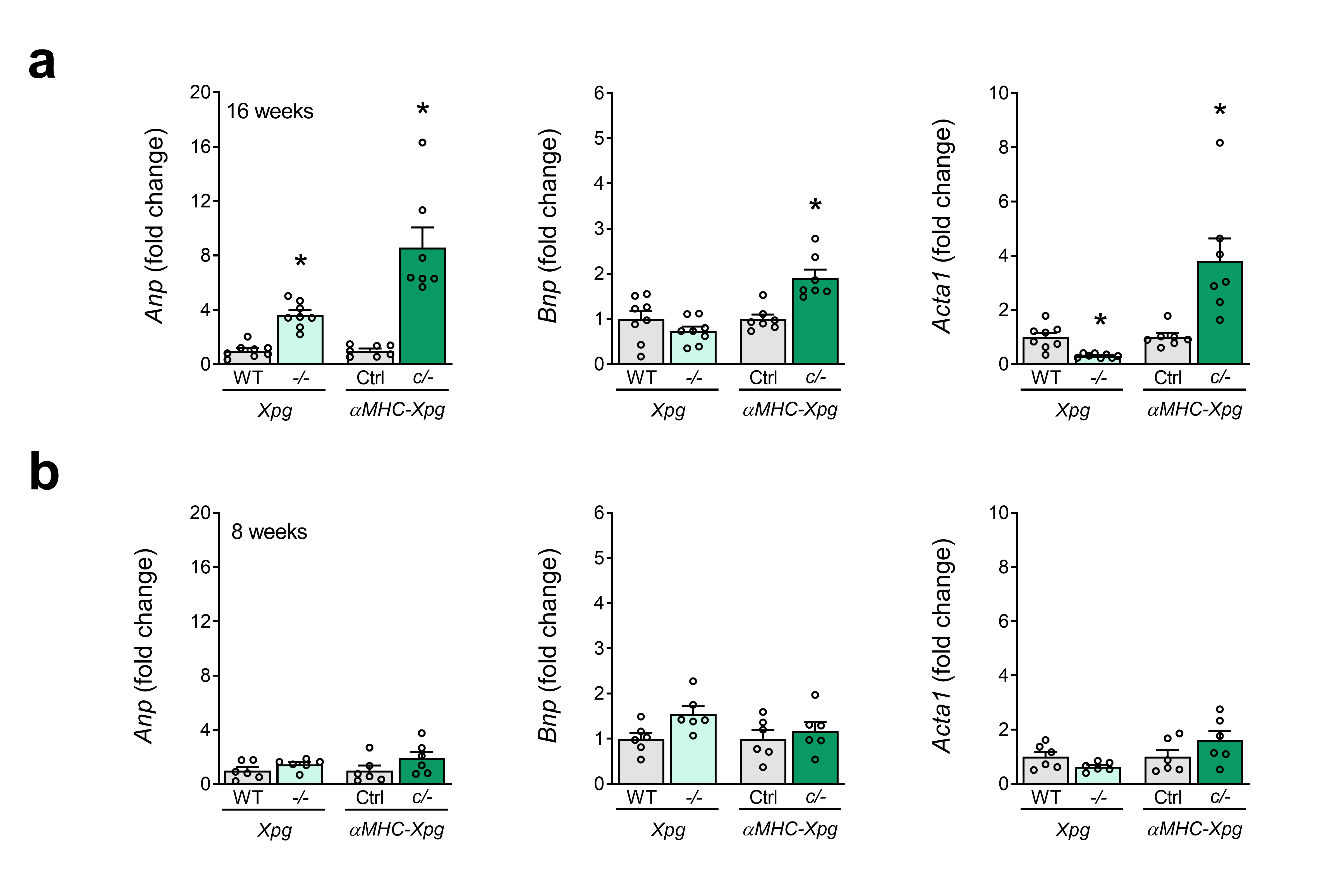
**

**Figure S6** Quantitative real-time PCR analysis showed increased levels of fetal genes in *Xpg* mutants at age 16 weeks. (*Study II*) (**a**) Relative expression levels of fetal genes atrial natriuretic peptide (*Anp*), brain natriuretic peptide (*Bnp*) and α-skeletal actin (*Acta1*) in 16-week old *Xpg^-/-^*, *αMHC-Xpg^c/-^* and corresponding control (n=7-8 animals/group). All values are corrected for *Hprt* expression and normalized to corresponding control. (**b**) Relative expression levels of *Anp*, *Bnp* and *Acta1* in 8-week-old *Xpg^-/-^*, *αMHC-Xpg^c/-^* and corresponding control (n=6 animals/group). All values are corrected for *Hprt* expression and normalized to corresponding control. Data are presented as mean±SEM. *P<0.05 vs. corresponding control by two-way ANOVA followed by SNK post-hoc testing.


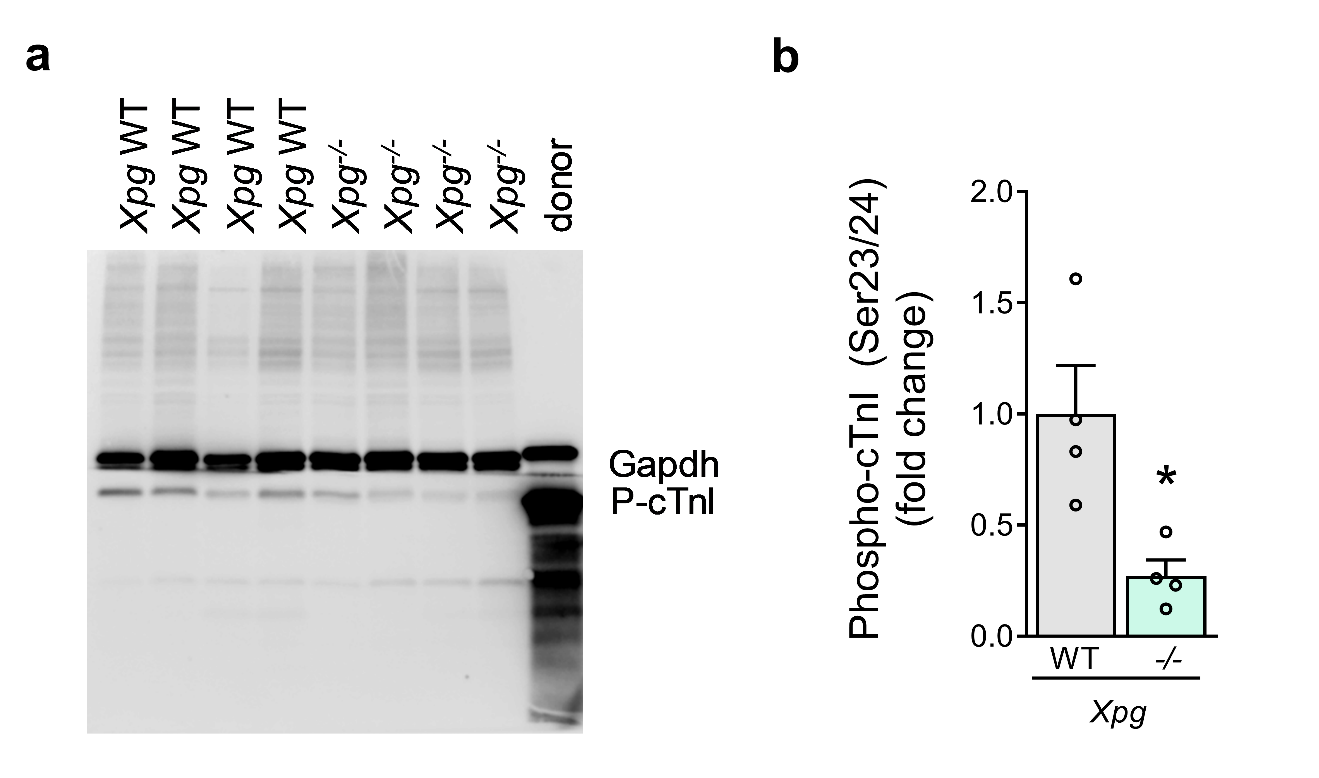


**Figure S7** Western blot analysis showed lower PKA-mediated phosphorylation in cardiac troponin I (cTnI) at Ser23/24 sites in *Xpg^-/--^* mice at age 16 weeks compared with corresponding control (*Study II*). (**a**) Full western blot of phosphorylated cTnI at PKA sites Ser23/24 (n=4 animals/group). Gapdh was used as loading control. Non-failing human donor sample (donor: highly phosphorylated) was included as internal marker. (**b**) Quantidication of phosphorylated cTnI at PKA sites Ser23/24. All values are normalized to control. Data are presented as mean±SEM. *P<0.05 vs. corresponding control by two-way ANOVA followed by SNK post-hoc testing.

**
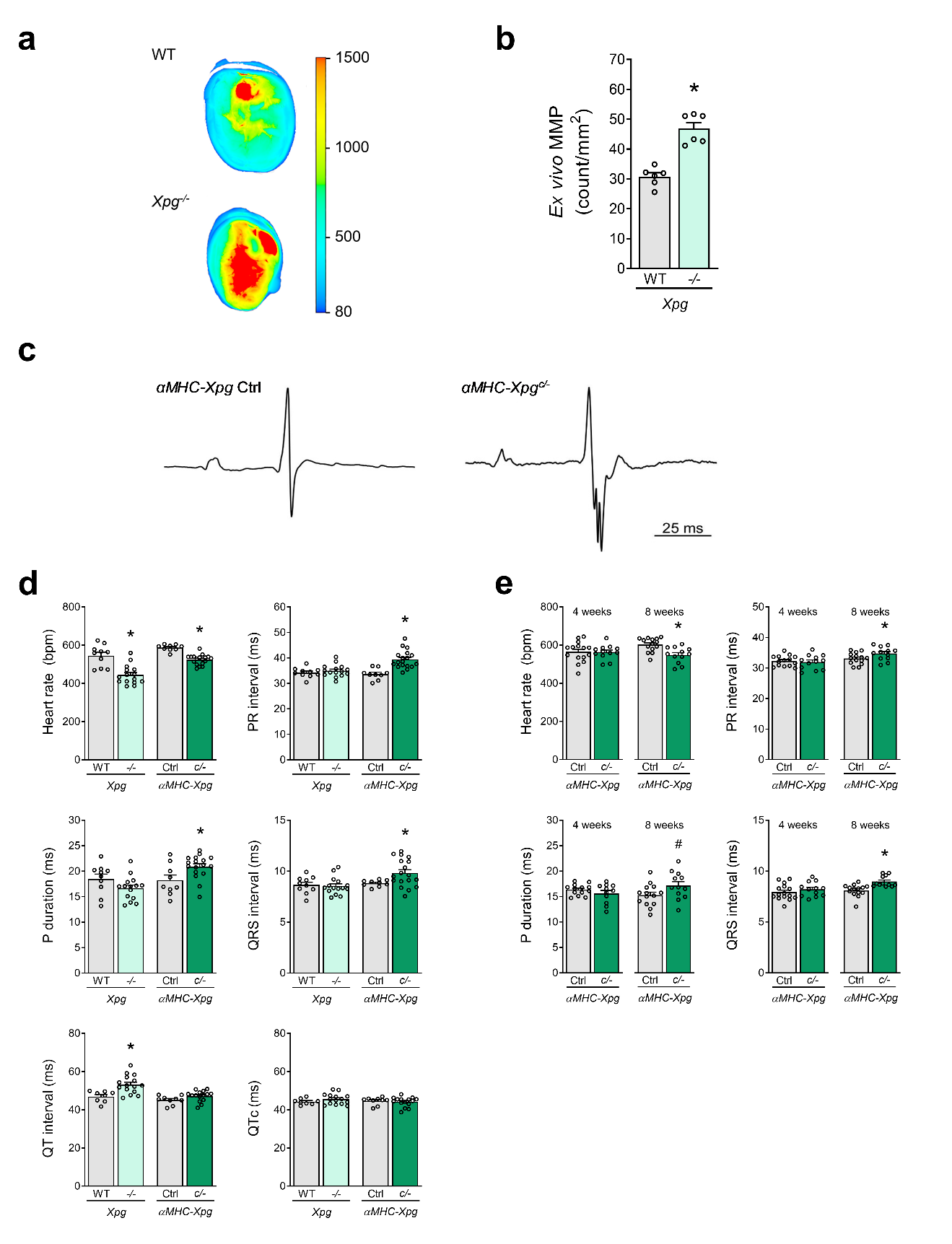
**

**Figure S8** Extracellular matrix turnover was enhanced in 16-week-old *Xpg^-/-^* mice. Fractionation of the QRS complex was observed in a subset of 16-week-old *Xpg^-/-^* and *αMHC-Xpg^c/-^* mice. However, only *αMHC-Xpg^c/-^* mice showed signs of cardiac conduction delay. (**a**) Representative images of the *ex vivo* visualization of MMP activity in *Xpg^-/-^* mice and corresponding control. (**b**) Quantification of the *ex vivo* visualization of MMP activity using Odyssey (*Study II*; n=6 animals/group). Data are presented as mean±SEM. (**c**) Typical surface ECG examples of 16-week-old *αMHC-Xpg^c/-^* mice and corresponding control. (**d**) Detailed analysis of surface ECG showed lowered heart rate in both *Xpg*-mutants at age 16 weeks and significantly prolonged PR interval, P duration and QRS interval in *αMHC-Xpg^c/-^* mice (*Study I*, lifespan study; n=9-18 animals/group). (**e**) Detailed analysis of surface ECG in 8-week-old *αMHC-Xpg^c/-^* mice revealed lowered heart, increased PR interval, P duration and QRS interval, while these parameters were unchanged in mice at 4 weeks of age (*Study I*, lifespan study; n=12-15 animals/group). Data are presented as mean±SEM. *P<0.05, # P=0.058 vs. corresponding control by Mann-Whitney Rank Sum test (b), two-way ANOVA followed by SNK post-hoc testing (d) and two-tailed, unpaired Student’s *t* test (e).

**
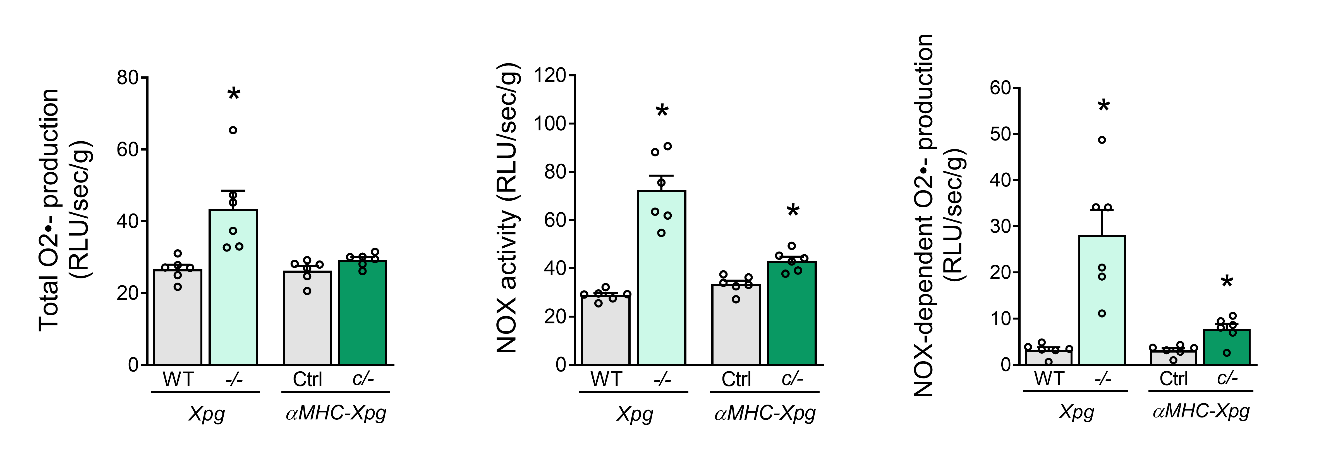
**

**Figure S9** Deficient DNA repair resulted in elevated oxidative stress in *Xpg* mutants at age 16 weeks. (*Study II*) Superoxide production is expressed as the relative light unit (RLU) per second per gram of protein (n=6 animals/group). Data are presented as mean±SEM. *P<0.05 vs. corresponding control by two-way ANOVA followed by SNK post-hoc testing.


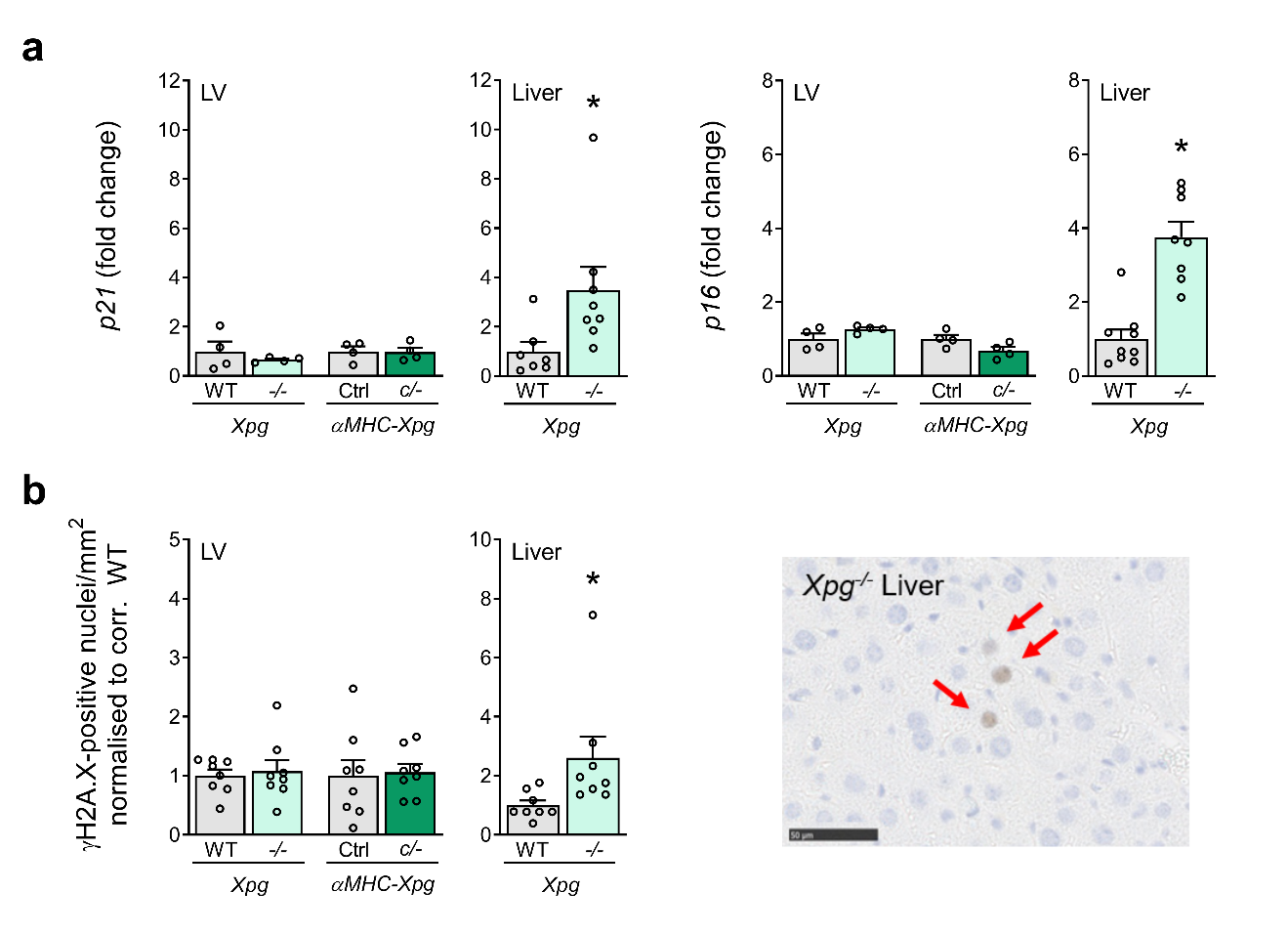


**Figure S10** Cardiac *p21* and *p16* expression levels are not elevated in *Xpg* mutants at age 16 weeks. In addition, DNA damage marker γH2A.X is also not altered. (*Study II*) (**a**) Quantitative real-time PCR analysis to determine relative expression levels of *p21* and *p16* in *Xpg^-/-^* and *αMHC-Xpg^c/-^* LV and *Xpg^-/-^* liver at age 16 weeks compared with corresponding control (LV, n=4 animals/group; liver, n=7-9 animals/group). All values are corrected for *Hprt*, *Gapdh* and *TubG2* expression and normalized to corresponding control. (**b**) Quantification of phosphorylation of the H2A.X histone (γH2A.X) in heart and liver sections (n=8 animals/group). Data are presented as mean±SEM. *P<0.05 vs. corresponding control by two-way ANOVA followed by SNK post-hoc testing.


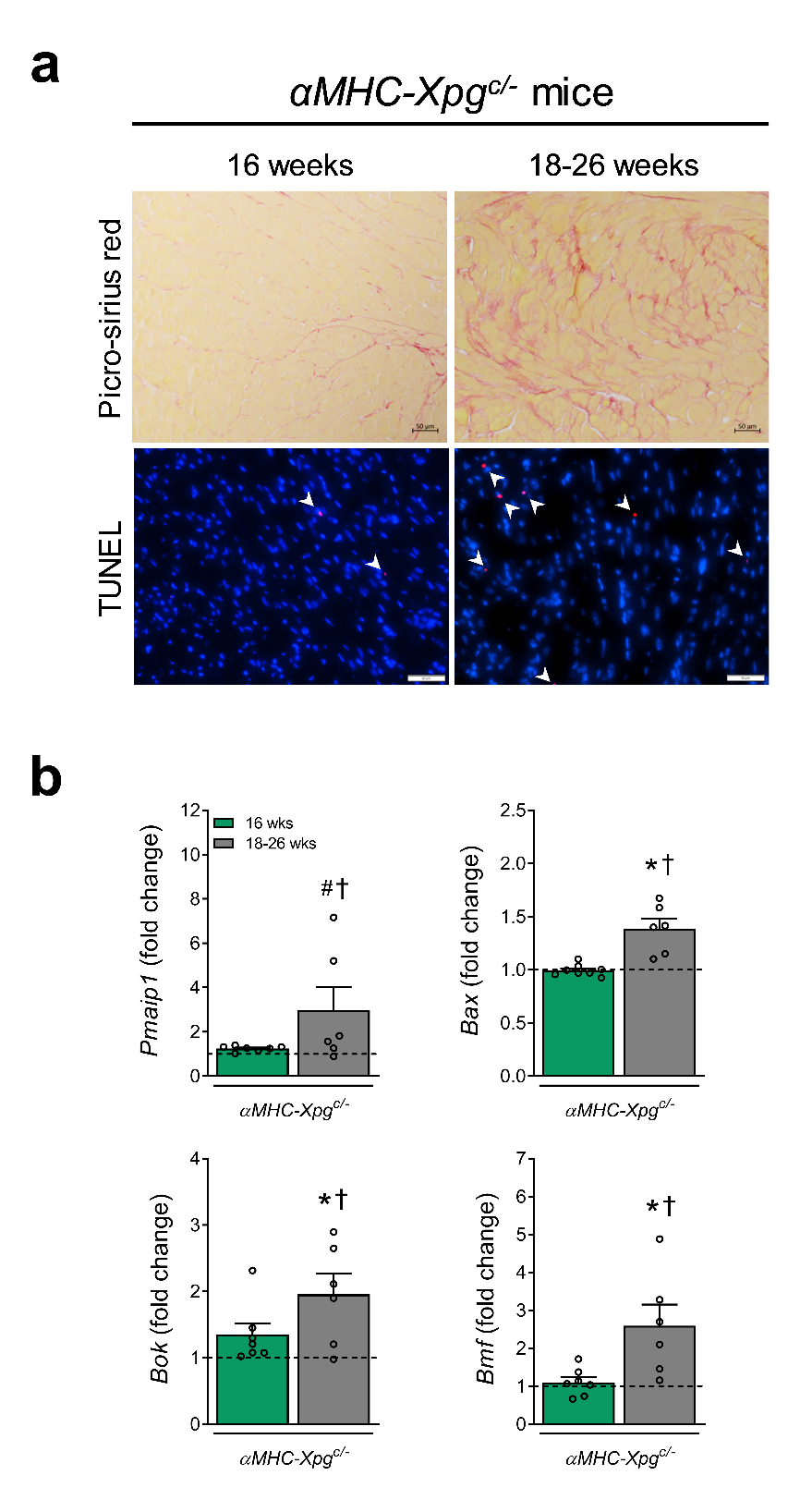


**Figure S11** Advanced age *αMHC-Xpg^c/-^* mice displayed marked increases in myocardial fibrosis and dramatic elevated levels of apoptotic cell death which was accompanied by increased expression levels of genes involved in the intrinsic apoptotic pathway. (**a**) Representative picro-sirius red- and TUNEL-stained LV sections of 16-week and 18-26-week-old *αMHC-Xpg^c/-^* mice. TUNEL-positive nuclei are indicated by arrows. For the sake of comparison we have repeated 16-week old *αMHC-Xpg^c/-^* mice in this figure. (**b**) Quantitative real-time PCR analysis showed increased relative expression levels of pro-apoptotic genes of the Bcl2 family (n=6-7 animals/group). *Pmaip1*, phorbol-12-myristate-13-acetate-induced protein 1; *Bax*, Bcl2 associated X protein; *Bok*, Bcl2-related ovarian killer protein; *Bmf*, Bcl2 modifying factor. All values are corrected for *Hprt* and *TubG2* expression and normalized to corresponding control (black dotted line). 16-week-old *αMHC-Xpg^c/-^* mice *Study II and* 18-26-week-old *αMHC-Xpg^c/-^* mice *Study I*, lifespan study. Data are presented as mean±SEM. *P<0.05, #P=0.052 vs. corresponding control; †P<0.05 vs. *αMHC-Xpg^c/-^* 16 week by one-way ANOVA followed by SNK post-hoc testing.

**
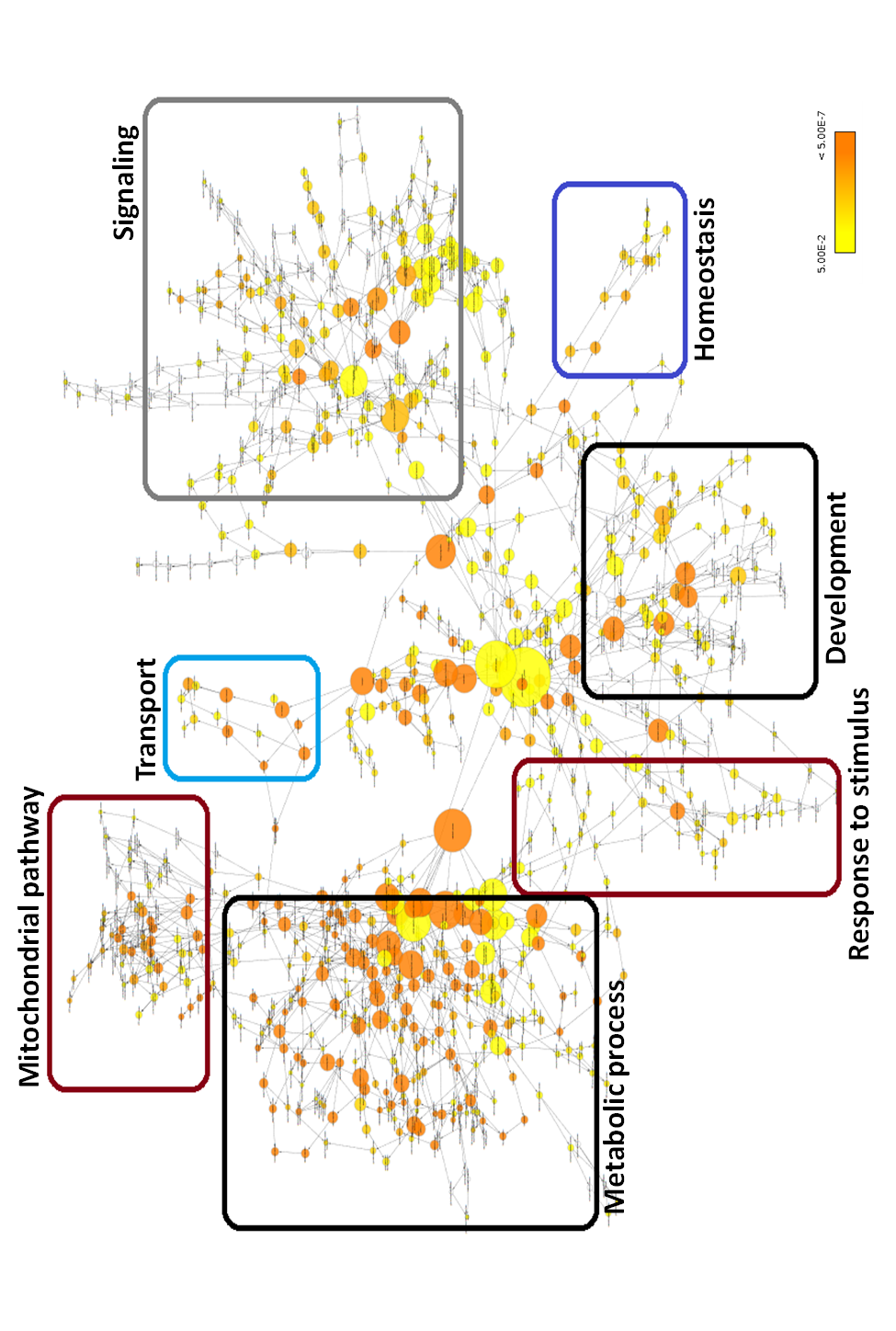
**

**Figure S12** Network visualisation of all DEGs (p-value <0.05) in *αMHC-Xpg^c/-^* mice. Node size is shown as proportional to the number of genes placed in the GO category. Colour denotes the p-value of each enriched GO terms (colour scale, right bottom, Benjamini & Hochberg FDR test), white nodes are not enriched.


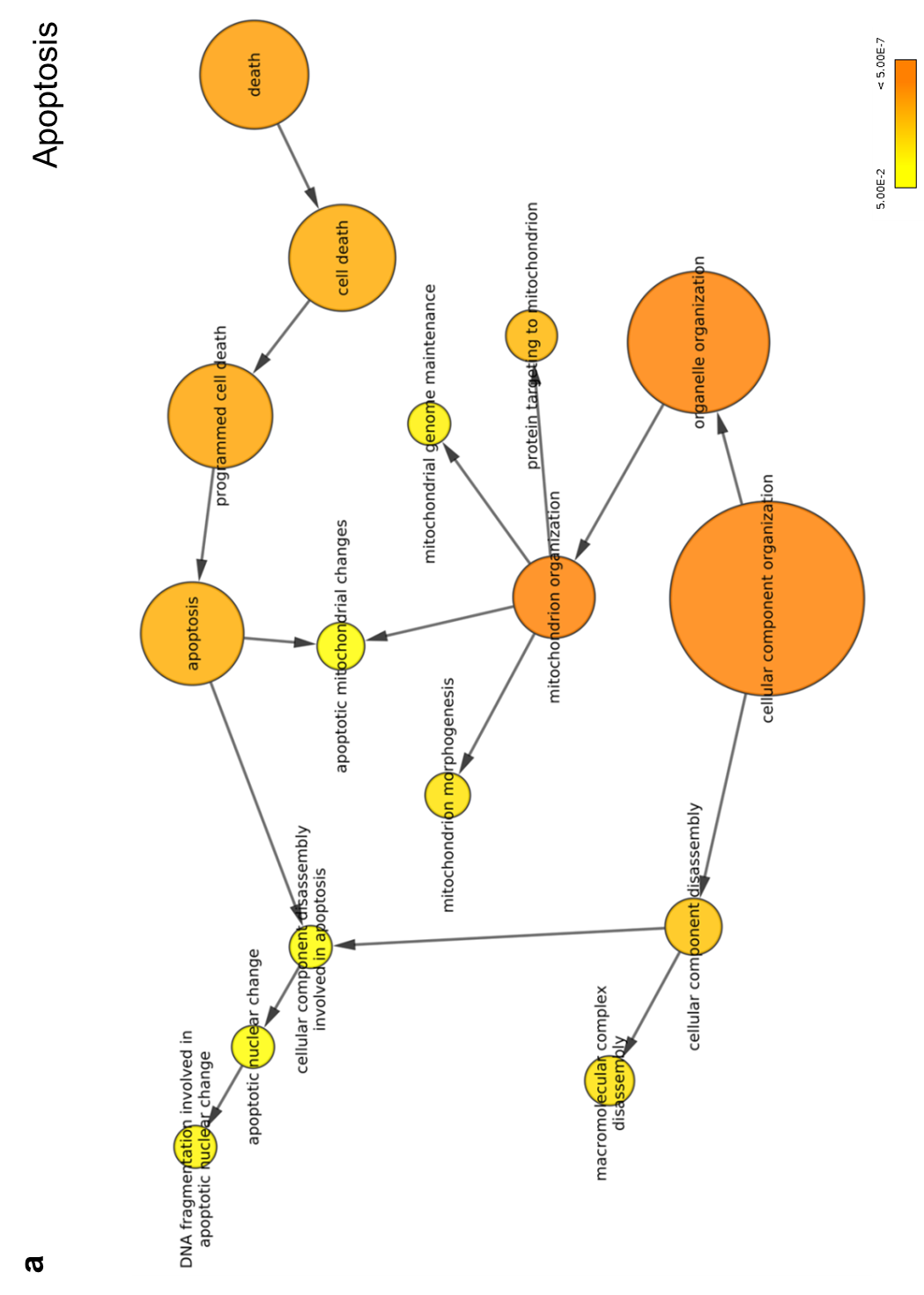


**
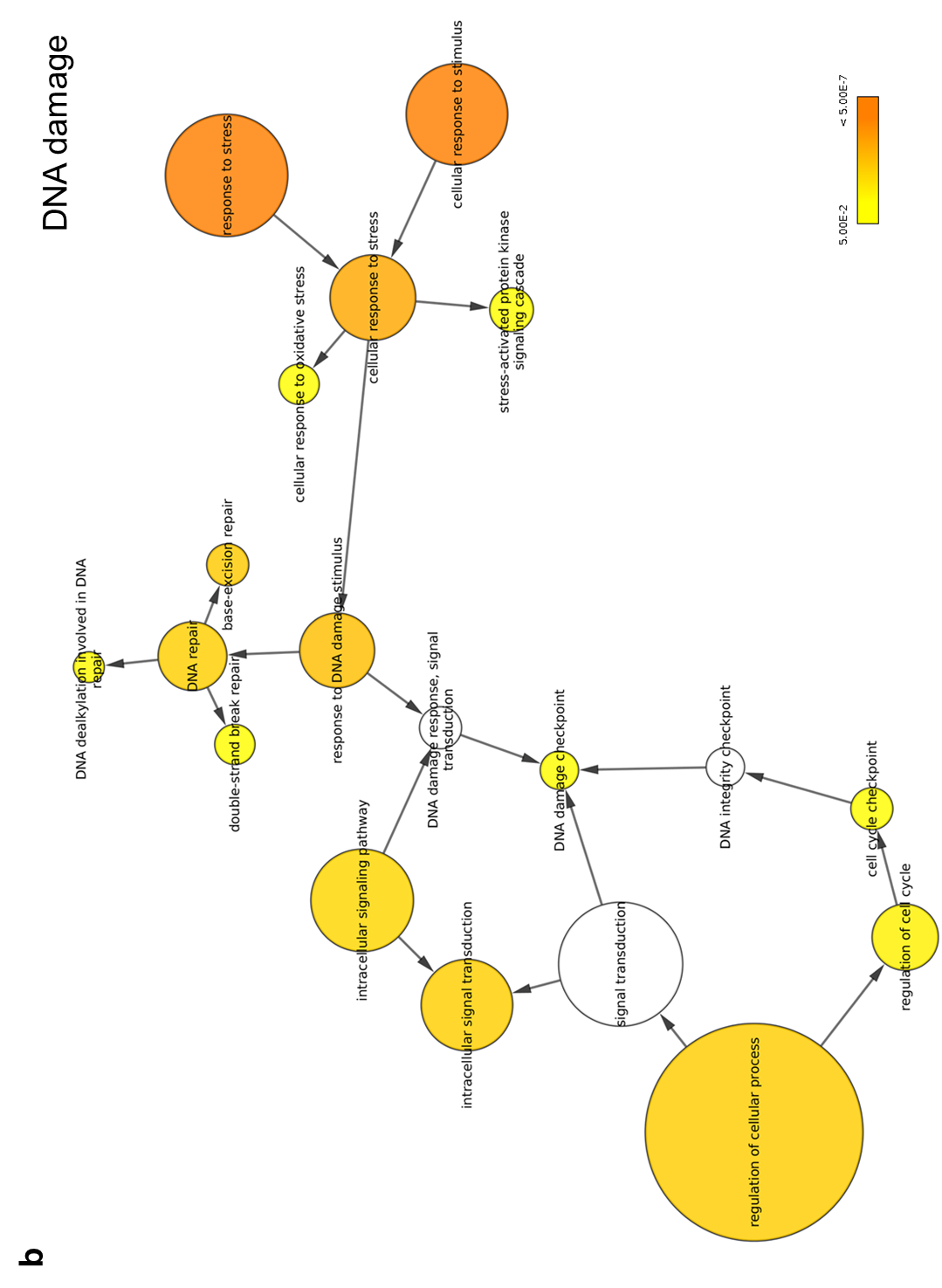
Figure S13** Enrichment of apoptosis (**a**) and DNA damage (**b**) subnetworks in *αMHC-Xpg^c/-^* mice.

In-depth visualisation of the apoptosis and DNA damage subnetwork in *αMHC-Xpg^c/-^* mice. Node size is shown as proportional to the number of genes placed in the GO category. Colour denotes the p-value of each enriched GO terms (colour scale, right bottom, Benjamini & Hochberg FDR test), white nodes are not enriched.

**
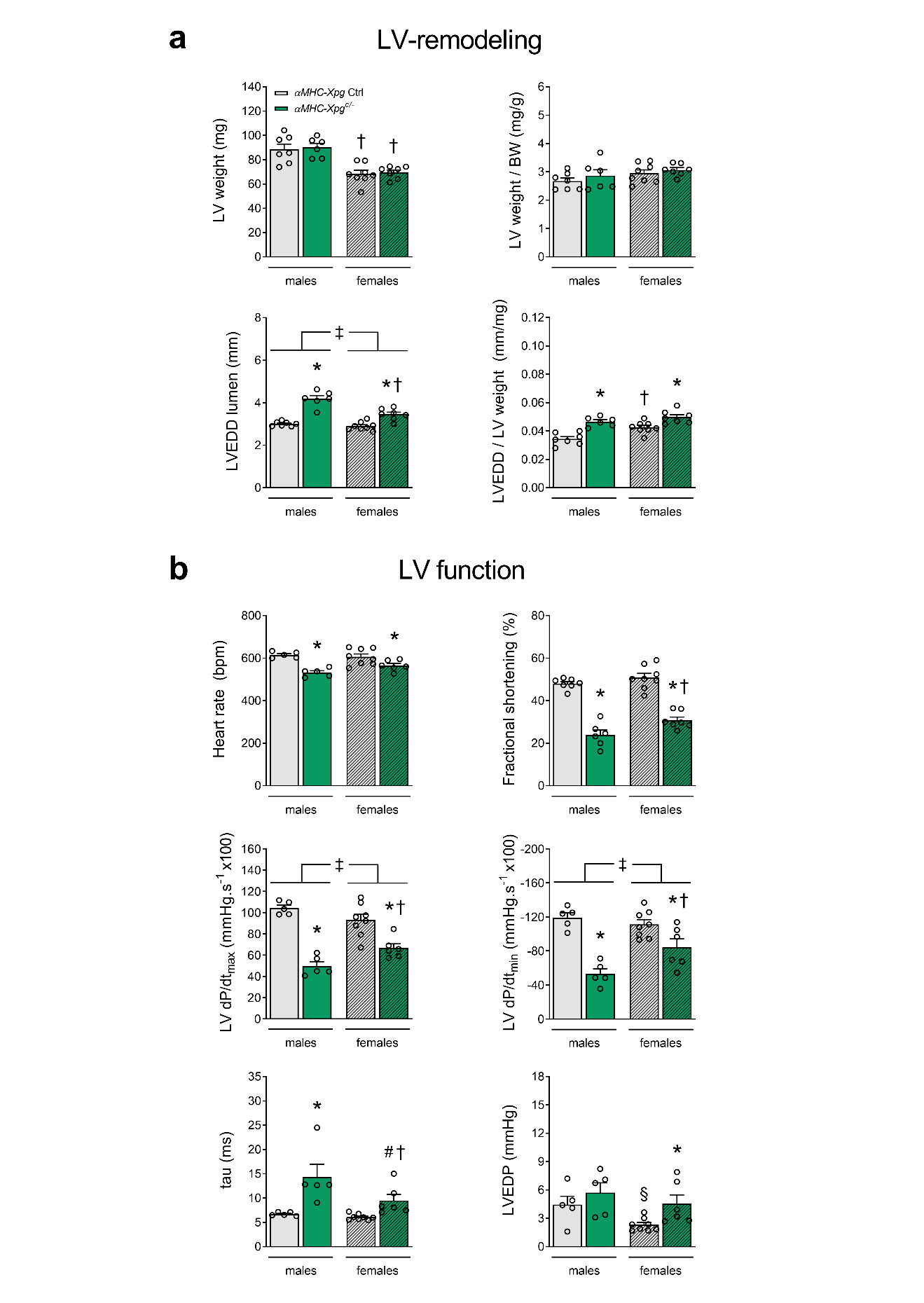
**

**Figure S14** The responses to DNA repair deficiency in LV-remodeling and LV dysfunction are hihly similar in *αMHC-Xpg^c/-^*  males and *αMHC-Xpg^c/-^*  females at 16 weeks of age (*Study II*). (**a**) Effect of *Xpg*--deficiency on LV mass and geometry and (**b**), hemodynamic parameters in 16-week-old *αMHC-Xpg^c/-^* mice and corresponding control (males: *αMHC-Xpg* Ctrl n=5-7, *αMHC-Xpg^c/-^* n=5-6 animals/group; females: *αMHC-Xpg* Ctrl n=8, *αMHC-Xpg^c/-^* n=6-7 animals/group). LV weight, left ventricular weight; BW, body weight; LVEDD, LV end-diastolic lumen diameter; LVdP/dt_max_, maximum rate of rise of LV pressure; LVdP/dt_min_, maximum rate of fall of LV pressure; tau, relaxation time constant; LVEDP, LV end-diastolic pressure. Data are presented as mean±SEM. *P<0.05, #P=0.054 vs. corresponding control; †P<0.05, §P=0.054 vs. corresponding male mice; ‡P<0.05 interaction genotype x sex by two-way ANOVA followed by SNK post-hoc testing.

**Table S1** Primer sequences used for quantitative RT-PCR.

| **Gene** | **Sense primer sequence** | **Antisense primer sequence** |
| --- | --- | --- |
| *Acta1* | TGACGTGTACATAGATTGACTCGTTT | TGGCTGGCTTTAATGCTTCA |
| *Anp* | TTCCTCGTCTTGGCCTTTTG | CCTCATCTTCTACCGGCATCTTC |
| *Bax* | TGAAGACAGGGGCCTTTTTG | AATTCGCCGGAGACACTCG |
| *Bmf* | AATCGAGTGTGGGCACCAAG | AGCTCCTCCACACACTGAGG |
| *Bnp* | GGGAGAACACGGCATCATTG | ACAGCACCTTCAGGAGATCCA |
| *Bok* | CATGGTTCATGCCCTGGTTG | CTTGAGGACATCCGTCCATCC |
| *Ercc1* | AGAACTTCGCCCTTCGTGTG | TGCTCATACGCCTTGTAGGTC |
| *Gapdh* | ACCACAGTCCATGCCATCAC | TCCACCACCCTGTTGCTGTA |
| *Hprt* | TCAGGAGAGAAAGATGTGATTGA | CAGCCAACACTGCTGAAACA |
| *p16* | CCCAACGCCCCGAACT | GCAGAAGAGCTGCTACGTGAA |
| *p21* | CAAGAGGCCCAGTACTTCCT | CAATCTGCGCTTGGAGTGAT |
| *Pmaip1* | GCAGAGCTACCAACCTGAGTTC | CTTTTGCGACTTCCCAGGCA |
| *TubG2* | CAGACCAACCACTGCTACAT | AGGGAATGAAGTTGGCCAGT |
| *Xpg* | GTATTTGATGGTGATGCTCC | TTCAGAGCCTGTCTCTTCAGAAAGG |

**Table S2** Differentially expressed TP53-target genes in advanced age (18-22 weeks) *αMHC-Xpg^c/-^* mice (n=3 animals/group).

| **ID** | **Genes in dataset** | **Expr Log Ratio** | **Prediction (based on measurement direction)** |
| --- | --- | --- | --- |
| ENSMUSG00000048521 | *Cxcr6* | 3.813 | Activated |
| ENSMUSG00000050335 | *Lgals3* | 3.173 | Inhibited |
| ENSMUSG00000031284 | *Pak3* | 3.107 | Activated |
| ENSMUSG00000021091 | *Serpina3n* | 2.984 | Activated |
| ENSMUSG00000027750 | *Postn* | 2.815 | Activated |
| ENSMUSG00000062380 | *Tubb3* | 2.691 | Affected |
| ENSMUSG00000079014 | *Serpina3i* | 2.677 | Activated |
| ENSMUSG00000031995 | *St14* | 2.514 | Activated |
| ENSMUSG00000039239 | *Tgfb2* | 2.401 | Activated |
| ENSMUSG00000020325 | *Fstl3* | 2.285 | Activated |
| ENSMUSG00000027656 | *Wisp2* | 2.243 | Affected |
| ENSMUSG00000018339 | *Gpx3* | 2.170 | Activated |
| ENSMUSG00000023092 | *Fhl1* | 2.087 | Activated |
| ENSMUSG00000030562 | *Nox4* | 1.997 | Affected |
| ENSMUSG00000019997 | *Ctgf* | 1.919 | Activated |
| ENSMUSG00000043613 | *Mmp3* | 1.911 | Inhibited |
| ENSMUSG00000052821 | *Cysltr1* | 1.894 | Activated |
| ENSMUSG00000000308 | *Ckmt1* | 1.873 | Activated |
| ENSMUSG00000039747 | *Orai2* | 1.814 | Affected |
| ENSMUSG00000023043 | *Krt18* | 1.813 | Inhibited |
| ENSMUSG00000022371 | *Col14a1* | 1.784 | Inhibited |
| ENSMUSG00000040569 | *Slc26a7* | 1.768 | Activated |
| ENSMUSG00000031548 | *Sfrp1* | 1.760 | Inhibited |
| ENSMUSG00000028864 | *Hgf* | 1.700 | Activated |
| ENSMUSG00000002897 | *Il17ra* | 1.689 | Inhibited |
| ENSMUSG00000068335 | *Dok1* | 1.688 | Activated |
| ENSMUSG00000026042 | *Col5a2* | 1.685 | Activated |
| ENSMUSG00000022037 | *Clu* | 1.668 | Activated |
| ENSMUSG00000024222 | *Fkbp5* | 1.656 | Activated |
| ENSMUSG00000020681 | *Ace* | 1.631 | Affected |
| ENSMUSG00000049281 | *Scn3b* | 1.631 | Activated |
| ENSMUSG00000021367 | *Edn1* | 1.616 | Activated |
| ENSMUSG00000029061 | *Mmp23* | 1.598 | Activated |
| ENSMUSG00000053279 | *Aldh1a1* | 1.578 | Inhibited |
| ENSMUSG00000028111 | *Ctsk* | 1.569 | Activated |
| ENSMUSG00000001131 | *Timp1* | 1.561 | Activated |
| ENSMUSG00000030717 | *Nupr1* | 1.557 | Inhibited |
| ENSMUSG00000002265 | *Peg3* | 1.556 | Activated |
| ENSMUSG00000040010 | *Slc7a5* | 1.544 | Affected |
| ENSMUSG00000064080 | *Fbln2* | 1.531 | Activated |
| ENSMUSG00000005087 | *Cd44* | 1.510 | Affected |
| ENSMUSG00000041736 | *Tspo* | 1.509 | Activated |
| ENSMUSG00000026580 | *Selp* | 1.498 | Inhibited |
| ENSMUSG00000036256 | *Igfbp7* | 1.490 | Affected |
| ENSMUSG00000029752 | *Asns* | 1.489 | Affected |
| ENSMUSG00000041324 | *Inhba* | 1.467 | Activated |
| ENSMUSG00000023224 | *Serping1* | 1.460 | Affected |
| ENSMUSG00000000031 | *H19* | 1.421 | Inhibited |
| ENSMUSG00000007613 | *Tgfbr1* | 1.392 | Affected |
| ENSMUSG00000024803 | *Ankrd1* | 1.390 | Activated |
| ENSMUSG00000047281 | *Sfn* | 1.388 | Activated |
| ENSMUSG00000007033 | *Hspa1l* | 1.386 | Inhibited |
| ENSMUSG00000022816 | *Fstl1* | 1.383 | Activated |
| ENSMUSG00000029161 | *Cgref1* | 1.380 | Activated |
| ENSMUSG00000045679 | *Pqlc3* | 1.368 | Affected |
| ENSMUSG00000021253 | *Tgfb3* | 1.359 | Inhibited |
| ENSMUSG00000033105 | *Lss* | 1.359 | Affected |
| ENSMUSG00000038205 | *Prkab2* | 1.350 | Activated |
| ENSMUSG00000003617 | *Cp* | 1.346 | Activated |
| ENSMUSG00000018042 | *Cyb5r3* | 1.342 | Activated |
| ENSMUSG00000026204 | *Ptprn* | 1.341 | Activated |
| ENSMUSG00000049382 | *Krt8* | 1.336 | Affected |
| ENSMUSG00000034926 | *Dhcr24* | 1.332 | Activated |
| ENSMUSG00000008393 | *Carhsp1* | 1.310 | Affected |
| ENSMUSG00000025473 | *Adam8* | 1.305 | Affected |
| ENSMUSG00000022474 | *Pmm1* | 1.289 | Affected |
| ENSMUSG00000027996 | *Sfrp2* | 1.286 | Inhibited |
| ENSMUSG00000040093 | *Bmf* | 1.267 | Inhibited |
| ENSMUSG00000038007 | *Acer2* | 1.231 | Activated |
| ENSMUSG00000044641 | *Pard6b* | 1.229 | Affected |
| ENSMUSG00000029860 | *Zyx* | 1.219 | Activated |
| ENSMUSG00000026249 | *Serpine2* | 1.212 | Activated |
| ENSMUSG00000016918 | *Sulf1* | 1.208 | Activated |
| ENSMUSG00000031740 | *Mmp2* | 1.207 | Affected |
| ENSMUSG00000059456 | *Ptk2b* | 1.204 | Activated |
| ENSMUSG00000029661 | *Col1a2* | 1.202 | Inhibited |
| ENSMUSG00000032092 | *Mpzl2* | 1.200 | Affected |
| ENSMUSG00000020901 | *Pik3r5* | 1.195 | Inhibited |
| ENSMUSG00000024659 | *Anxa1* | 1.189 | Activated |
| ENSMUSG00000037820 | *Tgm2* | 1.184 | Activated |
| ENSMUSG00000025912 | *Mybl1* | 1.165 | Activated |
| ENSMUSG00000035121 | *Neil2* | 1.163 | Affected |
| ENSMUSG00000029994 | *Anxa4* | 1.162 | Activated |
| ENSMUSG00000048376 | *F2r* | 1.155 | Activated |
| ENSMUSG00000001506 | *Col1a1* | 1.149 | Activated |
| ENSMUSG00000022665 | *Ccdc80* | 1.138 | Activated |
| ENSMUSG00000022510 | *Trp63* | 1.137 | Inhibited |
| ENSMUSG00000033581 | *Igf2bp2* | 1.131 | Affected |
| ENSMUSG00000037742 | *Eef1a1* | 1.128 | Activated |
| ENSMUSG00000021950 | *Anxa8* | 1.126 | Activated |
| ENSMUSG00000035783 | *Acta2* | 1.105 | Activated |
| ENSMUSG00000038400 | *Pmepa1* | 1.080 | Activated |
| ENSMUSG00000032715 | *Trib3* | 1.079 | Inhibited |
| ENSMUSG00000026278 | *Bok* | 1.076 | Activated |
| ENSMUSG00000021614 | *Vcan* | 1.046 | Activated |
| ENSMUSG00000035232 | *Pdk3* | 1.046 | Activated |
| ENSMUSG00000026479 | *Lamc2* | 1.039 | Activated |
| ENSMUSG00000030748 | *Il4ra* | 1.034 | Inhibited |
| ENSMUSG00000021196 | *Pfkp* | 1.017 | Inhibited |
| ENSMUSG00000031377 | *Bmx* | 1.016 | Activated |
| ENSMUSG00000031016 | *Wee1* | 1.016 | Inhibited |
| ENSMUSG00000021702 | *Thbs4* | 1.011 | Activated |
| ENSMUSG00000026043 | *Col3a1* | 1.010 | Activated |
| ENSMUSG00000024538 | *Ppic* | 0.997 | Activated |
| ENSMUSG00000047250 | *Ptgs1* | 0.997 | Inhibited |
| ENSMUSG00000020644 | *Id2* | 0.992 | Inhibited |
| ENSMUSG00000024486 | *Hbegf* | 0.991 | Activated |
| ENSMUSG00000022817 | *Itgb5* | 0.984 | Activated |
| ENSMUSG00000026193 | *Fn1* | 0.979 | Inhibited |
| ENSMUSG00000024521 | *Pmaip1* | 0.978 | Activated |
| ENSMUSG00000025880 | *Smad7* | 0.977 | Inhibited |
| ENSMUSG00000072214 | *Sept5* | 0.966 | Affected |
| ENSMUSG00000023067 | *Cdkn1a* | 0.966 | Activated |
| ENSMUSG00000061878 | *Sphk1* | 0.965 | Inhibited |
| ENSMUSG00000079037 | *Prnp* | 0.963 | Activated |
| ENSMUSG00000005533 | *Igf1r* | 0.961 | Activated |
| ENSMUSG00000016487 | *Ppfibp1* | 0.961 | Activated |
| ENSMUSG00000057329 | *Bcl2* | 0.952 | Inhibited |
| ENSMUSG00000025533 | *Asl* | 0.948 | Activated |
| ENSMUSG00000072825 | *Cep170b* | 0.945 | Affected |
| ENSMUSG00000074063 | *Osgin1* | 0.945 | Activated |
| ENSMUSG00000015143 | *Actn1* | 0.940 | Affected |
| ENSMUSG00000076441 | *Ass1* | 0.933 | Affected |
| ENSMUSG00000041801 | *Phlda3* | 0.932 | Activated |
| ENSMUSG00000020427 | *Igfbp3* | 0.916 | Activated |
| ENSMUSG00000030095 | *Tmem43* | 0.909 | Affected |
| ENSMUSG00000002257 | *Def6* | 0.906 | Affected |
| ENSMUSG00000024558 | *Mapk4* | 0.897 | Affected |
| ENSMUSG00000058624 | *Gda* | 0.896 | Activated |
| ENSMUSG00000031167 | *Rbm3* | 0.890 | Activated |
| ENSMUSG00000029381 | *Shroom3* | 0.888 | Activated |
| ENSMUSG00000022892 | *App* | 0.882 | Activated |
| ENSMUSG00000028645 | *Slc2a1* | 0.879 | Inhibited |
| ENSMUSG00000036777 | *Anln* | 0.877 | Activated |
| ENSMUSG00000021701 | *Plk2* | 0.877 | Activated |
| ENSMUSG00000038271 | *Iffo1* | 0.876 | Affected |
| ENSMUSG00000004446 | *Bid* | 0.876 | Activated |
| ENSMUSG00000017466 | *Timp2* | 0.875 | Inhibited |
| ENSMUSG00000003534 | *Ddr1* | 0.870 | Activated |
| ENSMUSG00000078566 | *Bnip3* | 0.869 | Inhibited |
| ENSMUSG00000034349 | *Smc4* | 0.860 | Inhibited |
| ENSMUSG00000032440 | *Tgfbr2* | 0.852 | Inhibited |
| ENSMUSG00000067818 | *Myl9* | 0.847 | Activated |
| ENSMUSG00000031502 | *Col4a1* | 0.839 | Activated |
| ENSMUSG00000025498 | *Irf7* | 0.839 | Activated |
| ENSMUSG00000029802 | *Abcg2* | 0.838 | Activated |
| ENSMUSG00000043733 | *Ptpn11* | 0.829 | Activated |
| ENSMUSG00000031431 | *Tsc22d3* | 0.829 | Activated |
| ENSMUSG00000020027 | *Socs2* | 0.826 | Inhibited |
| ENSMUSG00000024759 | *Atl3* | 0.823 | Activated |
| ENSMUSG00000032462 | *Pik3cb* | 0.818 | Inhibited |
| ENSMUSG00000021876 | *Rnase4* | 0.816 | Affected |
| ENSMUSG00000053398 | *Phgdh* | 0.813 | Inhibited |
| ENSMUSG00000045934 | *Mtmr11* | 0.811 | Affected |
| ENSMUSG00000030681 | *Mvp* | 0.809 | Inhibited |
| ENSMUSG00000029231 | *Pdgfra* | 0.807 | Activated |
| ENSMUSG00000029999 | *Tgfa* | 0.804 | Activated |
| ENSMUSG00000060147 | *Serpinb6a* | 0.795 | Activated |
| ENSMUSG00000097039 | *Pvt1* | 0.795 | Activated |
| ENSMUSG00000040613 | *Apobec1* | 0.794 | Activated |
| ENSMUSG00000007659 | *Bcl2l1* | 0.794 | Inhibited |
| ENSMUSG00000034593 | *Myo5a* | 0.792 | Activated |
| ENSMUSG00000031503 | *Col4a2* | 0.790 | Inhibited |
| ENSMUSG00000054808 | *Actn4* | 0.788 | Inhibited |
| ENSMUSG00000026872 | *Zeb2* | 0.787 | Inhibited |
| ENSMUSG00000038776 | *Ephx1* | 0.787 | Activated |
| ENSMUSG00000031327 | *Chic1* | 0.783 | Affected |
| ENSMUSG00000005413 | *Hmox1* | 0.781 | Activated |
| ENSMUSG00000027247 | *Arhgap1* | 0.769 | Affected |
| ENSMUSG00000030096 | *Slc6a6* | 0.767 | Inhibited |
| ENSMUSG00000044216 | *Kcnj4* | 0.765 | Activated |
| ENSMUSG00000031530 | *Dusp4* | 0.762 | Affected |
| ENSMUSG00000032374 | *Plod2* | 0.762 | Inhibited |
| ENSMUSG00000022098 | *Bmp1* | 0.761 | Activated |
| ENSMUSG00000030591 | *Psmd8* | 0.754 | Inhibited |
| ENSMUSG00000029484 | *Anxa3* | 0.743 | Inhibited |
| ENSMUSG00000027035 | *Cers6* | 0.743 | Activated |
| ENSMUSG00000019851 | *Perp* | 0.742 | Activated |
| ENSMUSG00000020900 | *Myh10* | 0.741 | Inhibited |
| ENSMUSG00000030852 | *Tacc2* | 0.735 | Activated |
| ENSMUSG00000028599 | *Tnfrsf1b* | 0.734 | Activated |
| ENSMUSG00000015950 | *Ncf1* | 0.734 | Inhibited |
| ENSMUSG00000034377 | *Tulp4* | 0.725 | Affected |
| ENSMUSG00000028780 | *Sema3c* | 0.722 | Affected |
| ENSMUSG00000004951 | *Hspb1* | 0.714 | Activated |
| ENSMUSG00000026773 | *Pfkfb3* | 0.712 | Inhibited |
| ENSMUSG00000015932 | *Dstn* | 0.712 | Inhibited |
| ENSMUSG00000024085 | *Man2a1* | 0.702 | Activated |
| ENSMUSG00000018819 | *Lsp1* | 0.697 | Activated |
| ENSMUSG00000032231 | *Anxa2* | 0.697 | Activated |
| ENSMUSG00000028466 | *Creb3* | 0.696 | Activated |
| ENSMUSG00000036867 | *Smad6* | 0.692 | Activated |
| ENSMUSG00000031400 | *G6pdx* | 0.692 | Affected |
| ENSMUSG00000000126 | *Wnt9a* | 0.689 | Affected |
| ENSMUSG00000063856 | *Gpx1* | 0.683 | Activated |
| ENSMUSG00000003541 | *Ier3* | 0.679 | Activated |
| ENSMUSG00000029767 | *Calu* | 0.677 | Activated |
| ENSMUSG00000048612 | *Myof* | 0.664 | Inhibited |
| ENSMUSG00000003131 | *Pafah1b2* | 0.662 | Activated |
| ENSMUSG00000021270 | *Hsp90aa1* | 0.659 | Inhibited |
| ENSMUSG00000032479 | *Map4* | 0.653 | Inhibited |
| ENSMUSG00000056131 | *Pgm3* | 0.649 | Inhibited |
| ENSMUSG00000037966 | *Ninj1* | 0.648 | Activated |
| ENSMUSG00000017774 | *Myo1c* | 0.645 | Activated |
| ENSMUSG00000036896 | *C1qc* | 0.638 | Activated |
| ENSMUSG00000033420 | *Antxr1* | 0.637 | Activated |
| ENSMUSG00000027536 | *Chmp4c* | 0.631 | Inhibited |
| ENSMUSG00000031628 | *Casp3* | 0.628 | Activated |
| ENSMUSG00000040562 | *Gstm2* | 0.627 | Activated |
| ENSMUSG00000023088 | *Abcc1* | 0.626 | Inhibited |
| ENSMUSG00000005871 | *Apc* | 0.616 | Inhibited |
| ENSMUSG00000032089 | *Il10ra* | 0.614 | Inhibited |
| ENSMUSG00000020380 | *Rad50* | 0.613 | Inhibited |
| ENSMUSG00000021282 | *Eif5* | 0.613 | Affected |
| ENSMUSG00000024778 | *Fas* | 0.611 | Activated |
| ENSMUSG00000020122 | *Egfr* | 0.610 | Activated |
| ENSMUSG00000026473 | *Glul* | 0.610 | Activated |
| ENSMUSG00000024587 | *Nars* | 0.607 | Affected |
| ENSMUSG00000002985 | *Apoe* | 0.607 | Inhibited |
| ENSMUSG00000025647 | *Shisa5* | 0.606 | Activated |
| ENSMUSG00000022610 | *Mapk12* | 0.602 | Activated |
| ENSMUSG00000030341 | *Tnfrsf1a* | 0.600 | Activated |
| ENSMUSG00000036894 | *Rap2b* | 0.599 | Affected |
| ENSMUSG00000000278 | *Scpep1* | 0.598 | Inhibited |
| ENSMUSG00000049134 | *Nrap* | 0.598 | Activated |
| ENSMUSG00000028944 | *Prkag2* | 0.595 | Affected |
| ENSMUSG00000039616 | *Mocos* | 0.589 | Affected |
| ENSMUSG00000058076 | *Sdhc* | -0.585 | Activated |
| ENSMUSG00000073700 | *Klhl21* | -0.585 | Affected |
| ENSMUSG00000026798 | *Coq4* | -0.587 | Activated |
| ENSMUSG00000028247 | *Coq3* | -0.589 | Activated |
| ENSMUSG00000042340 | *Ctf1* | -0.591 | Affected |
| ENSMUSG00000027452 | *Acss1* | -0.593 | Activated |
| ENSMUSG00000002660 | *Clpp* | -0.599 | Inhibited |
| ENSMUSG00000027618 | *Nfs1* | -0.599 | Activated |
| ENSMUSG00000036052 | *Dnajb5* | -0.600 | Activated |
| ENSMUSG00000002767 | *Mrpl2* | -0.600 | Activated |
| ENSMUSG00000000168 | *Dlat* | -0.603 | Activated |
| ENSMUSG00000026087 | *Mrpl30* | -0.606 | Activated |
| ENSMUSG00000020775 | *Mrpl38* | -0.611 | Activated |
| ENSMUSG00000028756 | *Pink1* | -0.625 | Activated |
| ENSMUSG00000027076 | *Timm10* | -0.627 | Activated |
| ENSMUSG00000028773 | *Fabp3* | -0.627 | Affected |
| ENSMUSG00000027472 | *Pdrg1* | -0.633 | Activated |
| ENSMUSG00000029177 | *Cenpa* | -0.635 | Activated |
| ENSMUSG00000026496 | *Parp1* | -0.637 | Affected |
| ENSMUSG00000026104 | *Stat1* | -0.637 | Affected |
| ENSMUSG00000031543 | *Ank1* | -0.638 | Inhibited |
| ENSMUSG00000024924 | *Vldlr* | -0.638 | Activated |
| ENSMUSG00000020415 | *Pttg1* | -0.639 | Inhibited |
| ENSMUSG00000003868 | *Ruvbl2* | -0.641 | Affected |
| ENSMUSG00000040018 | *Cox15* | -0.643 | Activated |
| ENSMUSG00000022235 | *Cmbl* | -0.645 | Inhibited |
| ENSMUSG00000025478 | *Dpysl4* | -0.649 | Inhibited |
| ENSMUSG00000002949 | *Timm44* | -0.654 | Activated |
| ENSMUSG00000035772 | *Mrps2* | -0.655 | Activated |
| ENSMUSG00000019927 | *Ube2d1* | -0.657 | Inhibited |
| ENSMUSG00000025781 | *Atp5c1* | -0.657 | Inhibited |
| ENSMUSG00000019179 | *Mdh2* | -0.659 | Affected |
| ENSMUSG00000050953 | *Gja1* | -0.674 | Inhibited |
| ENSMUSG00000024997 | *Prdx3* | -0.674 | Affected |
| ENSMUSG00000078317 | *F8a* | -0.674 | Affected |
| ENSMUSG00000042148 | *Cox10* | -0.674 | Activated |
| ENSMUSG00000022013 | *Dnajc15* | -0.674 | Activated |
| ENSMUSG00000003923 | *Tfam* | -0.678 | Affected |
| ENSMUSG00000045038 | *Prkce* | -0.679 | Inhibited |
| ENSMUSG00000020572 | *Nampt* | -0.682 | Inhibited |
| ENSMUSG00000023809 | *Rps6ka2* | -0.682 | Inhibited |
| ENSMUSG00000018566 | *Slc2a4* | -0.690 | Activated |
| ENSMUSG00000017754 | *Pltp* | -0.691 | Inhibited |
| ENSMUSG00000000088 | *Cox5a* | -0.696 | Activated |
| ENSMUSG00000062591 | *Tubb4a* | -0.700 | Affected |
| ENSMUSG00000022889 | *Mrpl39* | -0.703 | Activated |
| ENSMUSG00000025428 | *Atp5a1* | -0.704 | Activated |
| ENSMUSG00000078937 | *Cpt1b* | -0.707 | Activated |
| ENSMUSG00000067321 | *Gm7931* | -0.708 | Inhibited |
| ENSMUSG00000005354 | *Txn2* | -0.710 | Activated |
| ENSMUSG00000028527 | *Ak4* | -0.712 | Activated |
| ENSMUSG00000025486 | *Sirt3* | -0.713 | Activated |
| ENSMUSG00000032816 | *Igdcc4* | -0.714 | Affected |
| ENSMUSG00000038084 | *Opa1* | -0.716 | Activated |
| ENSMUSG00000039640 | *Mrpl12* | -0.718 | Activated |
| ENSMUSG00000026048 | *Ercc5* | -0.718 | Inhibited |
| ENSMUSG00000044894 | *Uqcrq* | -0.719 | Activated |
| ENSMUSG00000026610 | *Esrrg* | -0.720 | Activated |
| ENSMUSG00000020741 | *Cluh* | -0.725 | Affected |
| ENSMUSG00000004698 | *Hdac9* | -0.730 | Inhibited |
| ENSMUSG00000042709 | *Atpaf2* | -0.731 | Activated |
| ENSMUSG00000015016 | *Acsf3* | -0.733 | Activated |
| ENSMUSG00000021313 | *Ryr2* | -0.738 | Activated |
| ENSMUSG00000013936 | *Myl2* | -0.741 | Activated |
| ENSMUSG00000064326 | *Siva1* | -0.741 | Inhibited |
| ENSMUSG00000020544 | *Cox11* | -0.743 | Activated |
| ENSMUSG00000029486 | *Mrpl1* | -0.743 | Activated |
| ENSMUSG00000022742 | *Cpox* | -0.744 | Inhibited |
| ENSMUSG00000015112 | *Slc25a13* | -0.748 | Affected |
| ENSMUSG00000029319 | *Coq2* | -0.749 | Activated |
| ENSMUSG00000029632 | *Ndufa4* | -0.758 | Activated |
| ENSMUSG00000018893 | *Mb* | -0.762 | Affected |
| ENSMUSG00000030785 | *Cox6a2* | -0.762 | Activated |
| ENSMUSG00000000563 | *Atp5f1* | -0.765 | Activated |
| ENSMUSG00000031633 | *Slc25a4* | -0.766 | Activated |
| ENSMUSG00000020456 | *Ogdh* | -0.773 | Activated |
| ENSMUSG00000010914 | *Pdhx* | -0.779 | Activated |
| ENSMUSG00000069844 | *Sco1* | -0.782 | Activated |
| ENSMUSG00000037583 | *Nr0b2* | -0.784 | Inhibited |
| ENSMUSG00000026817 | *Ak1* | -0.784 | Inhibited |
| ENSMUSG00000061518 | *Cox5b* | -0.787 | Inhibited |
| ENSMUSG00000039883 | *Lrrc17* | -0.788 | Inhibited |
| ENSMUSG00000042010 | *Acacb* | -0.792 | Activated |
| ENSMUSG00000008136 | *Fhl2* | -0.792 | Inhibited |
| ENSMUSG00000024962 | *Vegfb* | -0.792 | Activated |
| ENSMUSG00000020329 | *Polrmt* | -0.793 | Activated |
| ENSMUSG00000054021 | *Sirt5* | -0.796 | Activated |
| ENSMUSG00000000489 | *Pdgfb* | -0.797 | Activated |
| ENSMUSG00000020475 | *Pgam2* | -0.797 | Activated |
| ENSMUSG00000033278 | *Ptprm* | -0.798 | Inhibited |
| ENSMUSG00000064354 | *mt-Co2* | -0.801 | Inhibited |
| ENSMUSG00000026475 | *Rgs16* | -0.801 | Inhibited |
| ENSMUSG00000019761 | *Krt10* | -0.807 | Affected |
| ENSMUSG00000022425 | *Enpp2* | -0.815 | Inhibited |
| ENSMUSG00000027305 | *Ndufaf1* | -0.818 | Activated |
| ENSMUSG00000026582 | *Sele* | -0.822 | Activated |
| ENSMUSG00000005980 | *Dnase1* | -0.823 | Activated |
| ENSMUSG00000005510 | *Ndufs3* | -0.825 | Activated |
| ENSMUSG00000038967 | *Pdk2* | -0.826 | Activated |
| ENSMUSG00000026389 | *Steap3* | -0.828 | Inhibited |
| ENSMUSG00000025271 | *Pfkfb1* | -0.829 | Activated |
| ENSMUSG00000009863 | *Sdhb* | -0.835 | Activated |
| ENSMUSG00000020593 | *Lpin1* | -0.838 | Inhibited |
| ENSMUSG00000050856 | *Atp5k* | -0.839 | Inhibited |
| ENSMUSG00000020664 | *Dld* | -0.839 | Affected |
| ENSMUSG00000018770 | *Atp5g3* | -0.840 | Activated |
| ENSMUSG00000033065 | *Pfkm* | -0.842 | Activated |
| ENSMUSG00000021748 | *Pdhb* | -0.843 | Activated |
| ENSMUSG00000038583 | *Pln* | -0.844 | Activated |
| ENSMUSG00000036983 | *Tfb1m* | -0.845 | Activated |
| ENSMUSG00000016252 | *Atp5e* | -0.848 | Activated |
| ENSMUSG00000059741 | *Myl3* | -0.849 | Activated |
| ENSMUSG00000025968 | *Ndufs1* | -0.850 | Activated |
| ENSMUSG00000026526 | *Fh1* | -0.853 | Activated |
| ENSMUSG00000052738 | *Suclg1* | -0.861 | Inhibited |
| ENSMUSG00000028832 | *Stmn1* | -0.861 | Activated |
| ENSMUSG00000020340 | *Cyfip2* | -0.862 | Inhibited |
| ENSMUSG00000025537 | *Phkg1* | -0.865 | Activated |
| ENSMUSG00000034880 | *Mrpl34* | -0.865 | Activated |
| ENSMUSG00000004069 | *Dnaja3* | -0.869 | Activated |
| ENSMUSG00000030612 | *Mrpl46* | -0.871 | Affected |
| ENSMUSG00000021619 | *Atg10* | -0.871 | Activated |
| ENSMUSG00000026895 | *Ndufa8* | -0.874 | Activated |
| ENSMUSG00000035069 | *Oma1* | -0.884 | Inhibited |
| ENSMUSG00000041733 | *Coq5* | -0.888 | Activated |
| ENSMUSG00000027774 | *Gfm1* | -0.891 | Activated |
| ENSMUSG00000054612 | *Mgmt* | -0.891 | Inhibited |
| ENSMUSG00000038462 | *Uqcrfs1* | -0.892 | Activated |
| ENSMUSG00000025980 | *Hspd1* | -0.900 | Activated |
| ENSMUSG00000022797 | *Tfrc* | -0.902 | Activated |
| ENSMUSG00000018574 | *Acadvl* | -0.905 | Inhibited |
| ENSMUSG00000059363 | *Fxn* | -0.907 | Affected |
| ENSMUSG00000005683 | *Cs* | -0.907 | Activated |
| ENSMUSG00000057388 | *Mrpl18* | -0.909 | Activated |
| ENSMUSG00000021577 | *Sdha* | -0.919 | Activated |
| ENSMUSG00000057897 | *Camk2b* | -0.924 | Inhibited |
| ENSMUSG00000032314 | *Etfa* | -0.927 | Inhibited |
| ENSMUSG00000023473 | *Celsr3* | -0.928 | Activated |
| ENSMUSG00000032047 | *Acat1* | -0.930 | Inhibited |
| ENSMUSG00000060093 | *Hist1h4a* | -0.935 | Inhibited |
| ENSMUSG00000078713 | *Tomm5* | -0.937 | Activated |
| ENSMUSG00000021622 | *Ckmt2* | -0.941 | Activated |
| ENSMUSG00000006057 | *Atp5g1* | -0.944 | Activated |
| ENSMUSG00000004789 | *Dlst* | -0.948 | Activated |
| ENSMUSG00000083820 | *Gm6415* | -0.951 | Activated |
| ENSMUSG00000021281 | *Tnfaip2* | -0.953 | Inhibited |
| ENSMUSG00000047875 | *Gpr157* | -0.962 | Inhibited |
| ENSMUSG00000023826 | *Park2* | -0.968 | Inhibited |
| ENSMUSG00000000340 | *Dbt* | -0.971 | Activated |
| ENSMUSG00000002870 | *Mcm2* | -0.971 | Activated |
| ENSMUSG00000062210 | *Tnfaip8* | -0.975 | Inhibited |
| ENSMUSG00000026784 | *Pdss1* | -0.979 | Activated |
| ENSMUSG00000014551 | *Mrps25* | -0.994 | Activated |
| ENSMUSG00000006715 | *Gmnn* | -0.994 | Affected |
| ENSMUSG00000040152 | *Thbs1* | -1.002 | Inhibited |
| ENSMUSG00000025651 | *Uqcrc1* | -1.006 | Activated |
| ENSMUSG00000049265 | *Kcnk3* | -1.007 | Activated |
| ENSMUSG00000000317 | *Bcl6b* | -1.011 | Activated |
| ENSMUSG00000017386 | *Traf4* | -1.014 | Inhibited |
| ENSMUSG00000020331 | *Hcn2* | -1.020 | Activated |
| ENSMUSG00000023951 | *Vegfa* | -1.021 | Activated |
| ENSMUSG00000030541 | *Idh2* | -1.022 | Affected |
| ENSMUSG00000021273 | *Fdft1* | -1.027 | Affected |
| ENSMUSG00000038418 | *Egr1* | -1.034 | Inhibited |
| ENSMUSG00000029513 | *Prkab1* | -1.034 | Inhibited |
| ENSMUSG00000020205 | *Phlda1* | -1.038 | Inhibited |
| ENSMUSG00000018796 | *Acsl1* | -1.040 | Activated |
| ENSMUSG00000036381 | *P2ry14* | -1.040 | Inhibited |
| ENSMUSG00000026880 | *Stom* | -1.046 | Inhibited |
| ENSMUSG00000030246 | *Ldhb* | -1.047 | Activated |
| ENSMUSG00000022110 | *Sucla2* | -1.047 | Activated |
| ENSMUSG00000076435 | *Acsf2* | -1.053 | Activated |
| ENSMUSG00000000628 | *Hk2* | -1.055 | Activated |
| ENSMUSG00000033610 | *Pank1* | -1.060 | Inhibited |
| ENSMUSG00000061838 | *Suclg2* | -1.062 | Activated |
| ENSMUSG00000033871 | *Ppargc1b* | -1.074 | Activated |
| ENSMUSG00000020333 | *Acsl6* | -1.078 | Activated |
| ENSMUSG00000029545 | *Acads* | -1.082 | Activated |
| ENSMUSG00000026489 | *Coq8a* | -1.091 | Inhibited |
| ENSMUSG00000045875 | *Adra1a* | -1.098 | Activated |
| ENSMUSG00000031137 | *Fgf13* | -1.102 | Activated |
| ENSMUSG00000006818 | *Sod2* | -1.104 | Inhibited |
| ENSMUSG00000026429 | *Ube2t* | -1.119 | Affected |
| ENSMUSG00000035314 | *Gdpd5* | -1.121 | Inhibited |
| ENSMUSG00000026770 | *Il2ra* | -1.133 | Activated |
| ENSMUSG00000053719 | *Klk1b26* | -1.139 | Activated |
| ENSMUSG00000059447 | *Hadhb* | -1.143 | Inhibited |
| ENSMUSG00000036390 | *Gadd45a* | -1.150 | Inhibited |
| ENSMUSG00000059824 | *Dbp* | -1.167 | Inhibited |
| ENSMUSG00000062908 | *Acadm* | -1.183 | Activated |
| ENSMUSG00000025488 | *Cox8b* | -1.215 | Activated |
| ENSMUSG00000028607 | *Cpt2* | -1.219 | Activated |
| ENSMUSG00000001604 | *Tcea3* | -1.233 | Inhibited |
| ENSMUSG00000025745 | *Hadha* | -1.238 | Inhibited |
| ENSMUSG00000028737 | *Aldh4a1* | -1.251 | Inhibited |
| ENSMUSG00000041650 | *Pcca* | -1.262 | Inhibited |
| ENSMUSG00000020623 | *Map2k6* | -1.264 | Activated |
| ENSMUSG00000060600 | *Eno3* | -1.266 | Activated |
| ENSMUSG00000027203 | *Dut* | -1.270 | Activated |
| ENSMUSG00000026605 | *Cenpf* | -1.315 | Activated |
| ENSMUSG00000038240 | *Pdss2* | -1.358 | Activated |
| ENSMUSG00000030399 | *Ckm* | -1.408 | Inhibited |
| ENSMUSG00000027984 | *Hadh* | -1.412 | Inhibited |
| ENSMUSG00000029591 | *Ung* | -1.429 | Activated |
| ENSMUSG00000028017 | *Egf* | -1.471 | Inhibited |
| ENSMUSG00000074218 | *Cox7a1* | -1.521 | Activated |
| ENSMUSG00000005628 | *Tmod4* | -1.586 | Activated |
| ENSMUSG00000053898 | *Ech1* | -1.596 | Inhibited |
| ENSMUSG00000030972 | *Acsm5* | -1.610 | Activated |
| ENSMUSG00000037490 | *Slc2a12* | -1.658 | Activated |
| ENSMUSG00000036880 | *Acaa2* | -1.761 | Inhibited |
| ENSMUSG00000030357 | *Fkbp4* | -1.967 | Activated |

**Table S3** Significant genetic variants for LVEDD with accompanying information for fractional shortening from the population-based Rotterdam Study. LVEDD, LV end-diastolic lumen diameter; FS, fractional shortening.

| **MarkerName** | **Chromosome** | **Position** | **Allele1** | **Allele2** | **Freq1** | **Effect LVEDD** | **StdErr**  **LVEDD** | **P-value LVEDD** | **EffectFS** | **StdErr**  **FS** | **P-value**  **FS** | **Gene** |
| --- | --- | --- | --- | --- | --- | --- | --- | --- | --- | --- | --- | --- |
| 19:46002456 | 19 | 46002456 | t | g | 0.072 | 0.372 | 0.169 | 0.028 | 0.168 | 0.194 | 0.386 | *ERCC1* |
| **19:45967369** | **19** | **45967369** | **c** | **g** | **0.241** | **0.295** | **0.102** | **0.004** | **-0.099** | **0.117** | **0.395** | ***ERCC1*** |
| 19:46003066 | 19 | 46003066 | t | g | 0.928 | -0.365 | 0.170 | 0.031 | -0.163 | 0.195 | 0.403 | *ERCC1* |
| 19:45997347 | 19 | 45997347 | t | c | 0.928 | -0.367 | 0.169 | 0.030 | -0.160 | 0.194 | 0.408 | *ERCC1* |
| 19:45968607 | 19 | 45968607 | t | c | 0.928 | -0.386 | 0.166 | 0.020 | -0.131 | 0.190 | 0.490 | *ERCC1* |
| 19:45967290 | 19 | 45967290 | a | g | 0.554 | -0.176 | 0.087 | 0.044 | -0.061 | 0.100 | 0.541 | *ERCC1* |
| 19:45972861 | 19 | 45972861 | a | g | 0.553 | -0.178 | 0.087 | 0.041 | -0.058 | 0.100 | 0.560 | *ERCC1* |
| 19:45966351 | 19 | 45966351 | a | c | 0.554 | -0.182 | 0.087 | 0.037 | -0.057 | 0.100 | 0.566 | *ERCC1* |
| 19:45974593 | 19 | 45974593 | t | c | 0.553 | -0.185 | 0.087 | 0.033 | -0.057 | 0.100 | 0.570 | *ERCC1* |
| 19:45974668 | 19 | 45974668 | t | c | 0.447 | 0.185 | 0.087 | 0.033 | 0.056 | 0.099 | 0.571 | *ERCC1* |
| 19:45974809 | 19 | 45974809 | a | g | 0.553 | -0.185 | 0.087 | 0.033 | -0.056 | 0.099 | 0.571 | *ERCC1* |
| 19:45972881 | 19 | 45972881 | a | c | 0.553 | -0.184 | 0.087 | 0.035 | -0.055 | 0.100 | 0.583 | *ERCC1* |
| 19:45968798 | 19 | 45968798 | t | c | 0.553 | -0.185 | 0.087 | 0.034 | -0.055 | 0.100 | 0.584 | *ERCC1* |
| 19:45969490 | 19 | 45969490 | c | g | 0.447 | 0.185 | 0.087 | 0.034 | 0.055 | 0.100 | 0.585 | *ERCC1* |
| 19:45972039 | 19 | 45972039 | a | c | 0.553 | -0.185 | 0.087 | 0.034 | -0.054 | 0.100 | 0.586 | *ERCC1* |
| 19:45981102 | 19 | 45981102 | c | g | 0.927 | -0.402 | 0.167 | 0.016 | -0.102 | 0.192 | 0.593 | *ERCC1* |
| 19:45981105 | 19 | 45981105 | t | c | 0.073 | 0.402 | 0.167 | 0.016 | 0.102 | 0.192 | 0.593 | *ERCC1* |
| 19:45982373 | 19 | 45982373 | t | g | 0.927 | -0.393 | 0.167 | 0.018 | -0.099 | 0.191 | 0.605 | *ERCC1* |
| 19:45985351 | 19 | 45985351 | t | c | 0.927 | -0.394 | 0.167 | 0.018 | -0.099 | 0.191 | 0.605 | *ERCC1* |
| 19:45984603 | 19 | 45984603 | c | g | 0.927 | -0.404 | 0.167 | 0.015 | -0.098 | 0.191 | 0.610 | *ERCC1* |
| 19:45971095 | 19 | 45971095 | a | g | 0.118 | 0.331 | 0.133 | 0.012 | 0.070 | 0.152 | 0.647 | *ERCC1* |
| 19:45973056 | 19 | 45973056 | t | c | 0.882 | -0.332 | 0.132 | 0.012 | -0.069 | 0.152 | 0.647 | *ERCC1* |
| 19:45972408 | 19 | 45972408 | c | g | 0.883 | -0.332 | 0.132 | 0.012 | -0.069 | 0.152 | 0.648 | *ERCC1* |
| 19:45973166 | 19 | 45973166 | c | g | 0.118 | 0.331 | 0.132 | 0.013 | 0.068 | 0.152 | 0.652 | *ERCC1* |
| 19:45966174 | 19 | 45966174 | a | c | 0.080 | 0.415 | 0.159 | 0.009 | 0.065 | 0.182 | 0.721 | *ERCC1* |
| **19:45976718** | **19** | **45976718** | **t** | **c** | **0.846** | **-0.234** | **0.119** | **0.050** | **0.034** | **0.137** | **0.806** | ***ERCC1*** |
| 13:103568587 | 13 | 103568587 | a | g | 0.028 | -0.635 | 0.260 | 0.015 | -0.754 | 0.301 | 0.012 | *XPG* |
| 13:103561656 | 13 | 103561656 | t | g | 0.029 | -0.608 | 0.258 | 0.019 | -0.736 | 0.298 | 0.014 | *XPG* |
| 13:103559368 | 13 | 103559368 | a | g | 0.972 | 0.598 | 0.260 | 0.021 | 0.720 | 0.300 | 0.016 | *XPG* |
| 13:103564469 | 13 | 103564469 | a | g | 0.972 | 0.639 | 0.261 | 0.014 | 0.721 | 0.301 | 0.017 | *XPG* |
| 13:103563092 | 13 | 103563092 | t | c | 0.028 | -0.645 | 0.260 | 0.013 | -0.702 | 0.301 | 0.020 | *XPG* |
| **13:103467184** | **13** | **103467184** | **a** | **g** | **0.990** | **-1.132** | **0.545** | **0.038** | **0.811** | **0.589** | **0.169** | ***XPG*** |
| 13:103559910 | 13 | 103559910 | c | g | 0.118 | 0.357 | 0.132 | 0.007 | 0.153 | 0.152 | 0.311 | *XPG* |
| **13:103457497** | **13** | **103457497** | **c** | **g** | **0.028** | **0.628** | **0.275** | **0.022** | **-0.254** | **0.314** | **0.418** | ***XPG*** |
| **13:103527113** | **13** | **103527113** | **a** | **g** | **0.842** | **-0.266** | **0.124** | **0.032** | **0.114** | **0.142** | **0.422** | ***XPG*** |
| 13:103561362 | 13 | 103561362 | a | g | 0.666 | -0.201 | 0.090 | 0.025 | -0.078 | 0.103 | 0.446 | *XPG* |
| 13:103476337 | 13 | 103476337 | a | g | 0.911 | 0.306 | 0.150 | 0.041 | 0.101 | 0.172 | 0.557 | *XPG* |
| **13:103548761** | **13** | **103548761** | **a** | **g** | **0.981** | **-0.949** | **0.336** | **0.005** | **0.208** | **0.384** | **0.587** | ***XPG*** |
| 13:103504270 | 13 | 103504270 | a | g | 0.020 | -0.682 | 0.320 | 0.033 | -0.189 | 0.371 | 0.611 | *XPG* |
| 13:103568385 | 13 | 103568385 | a | g | 0.933 | -0.481 | 0.169 | 0.005 | -0.067 | 0.195 | 0.732 | *XPG* |

**Table S4** Significant genetic variants for fractional shortening with accompanying information for LVEDD from the population-based Rotterdam Study. FS, fractional shortening; LVEDD, LV end-diastolic lumen diameter.

| **Marker Name** | **Chromosome** | **Position** | **Allele1** | **Allele2** | **Freq1** | **Effect FS** | **StdErr FS** | **p-value FS** | **Effect LVEDD** | **StdErr LVEDD** | **p-value LVEDD** | **Gene** |
| --- | --- | --- | --- | --- | --- | --- | --- | --- | --- | --- | --- | --- |
| **19:45877788** | **19** | **45877788** | **a** | **g** | **0.2444** | **0.276** | **0.116** | **0.017** | **-0.129** | **0.101** | **0.199** | ***ERCC1*** |
| **19:45879195** | **19** | **45879195** | **t** | **g** | **0.941** | **-0.431** | **0.220** | **0.050** | **0.119** | **0.191** | **0.531** | ***ERCC1*** |
| **19:45884546** | **19** | **45884546** | **t** | **c** | **0.2454** | **0.278** | **0.116** | **0.017** | **-0.130** | **0.101** | **0.198** | ***ERCC1*** |
| **19:45888808** | **19** | **45888808** | **t** | **c** | **0.0261** | **-0.864** | **0.324** | **0.008** | **0.220** | **0.281** | **0.434** | ***ERCC1*** |
| **19:45896834** | **19** | **45896834** | **c** | **g** | **0.0753** | **0.428** | **0.196** | **0.029** | **-0.062** | **0.171** | **0.719** | ***ERCC1*** |
| **19:45914174** | **19** | **45914174** | **t** | **c** | **0.0193** | **-0.994** | **0.383** | **0.010** | **0.454** | **0.334** | **0.173** | ***ERCC1*** |
| 19:45962799 | 19 | 45962799 | a | g | 0.7622 | -0.233 | 0.116 | 0.045 | -0.036 | 0.101 | 0.720 | *ERCC1* |
| 19:45963055 | 19 | 45963055 | t | g | 0.7622 | -0.233 | 0.116 | 0.045 | -0.037 | 0.101 | 0.718 | *ERCC1* |
| 19:46020421 | 19 | 46020421 | a | t | 0.4048 | -0.211 | 0.100 | 0.034 | -0.020 | 0.087 | 0.814 | *ERCC1* |
| 19:46027752 | 19 | 46027752 | t | c | 0.7416 | 0.221 | 0.111 | 0.046 | 0.007 | 0.097 | 0.940 | *ERCC1* |
| 19:46028354 | 19 | 46028354 | a | c | 0.2582 | -0.226 | 0.111 | 0.041 | -0.006 | 0.097 | 0.953 | *ERCC1* |
| 19:46029981 | 19 | 46029981 | t | c | 0.2584 | -0.220 | 0.111 | 0.047 | -0.008 | 0.097 | 0.938 | *ERCC1* |
| 19:46030154 | 19 | 46030154 | a | g | 0.7416 | 0.219 | 0.111 | 0.048 | 0.008 | 0.097 | 0.936 | *ERCC1* |
| 13:103559368 | 13 | 103559368 | a | g | 0.972 | 0.720 | 0.300 | 0.016 | 0.598 | 0.260 | 0.021 | *XPG* |
| 13:103561656 | 13 | 103561656 | t | g | 0.0282 | -0.736 | 0.298 | 0.014 | -0.608 | 0.258 | 0.019 | *XPG* |
| 13:103563092 | 13 | 103563092 | t | c | 0.0279 | -0.702 | 0.301 | 0.020 | -0.645 | 0.260 | 0.013 | *XPG* |
| 13:103564469 | 13 | 103564469 | a | g | 0.9722 | 0.721 | 0.301 | 0.017 | 0.639 | 0.261 | 0.014 | *XPG* |
| 13:103568587 | 13 | 103568587 | a | g | 0.0277 | -0.754 | 0.301 | 0.012 | -0.635 | 0.260 | 0.015 | *XPG* |
