# Supplementary material for: DNA repair in cardiomyocytes is critical for maintaining cardiac function in mice
Source: Aging Cell. 2023 Feb 8;22(3):e13768. doi: 10.1111/acel.13768 (PMC10014058; doi:10.1111/acel.13768)
Supplement: Supplementary file 2 — Appendix S2. [file ACEL-22-e13768-s001.docx]

***Aging Cell***

**Expanded Methods**

**DNA repair in cardiomyocytes is critical for maintaining cardiac function in mice**

**Running title:** DNA repair and cardiac function

Martine de Boer^1^, Maaike te Lintel Hekkert^1^, Jiang Chang^2^, Bibi S. van Thiel^2,3,4^, Leonie Martens^5^, Maxime M. Bos^6^, Marion G.J. de Kleijnen^1^, Yanto Ridwan^2,7^, Yanti Octavia^1^, Elza D. van Deel^8^, Lau A. Blonden^1^, Renata M.C. Brandt^2^, Sander Barnhoorn^2^, Paula K. Bautista-Niño^4,9^, Ilona Krabbendam-Peters^1^, Rianne Wolswinkel^10^, Banafsheh Arshi^6^, Mohsen Ghanbari^6^, Christian Kupatt^11,12,13^, Leon J. de Windt^14^, A.H. Jan Danser^4^, Ingrid van der Pluijm^2,3^, Carol Ann Remme^10^, Monika Stoll^5,15^, Joris Pothof^2^, Anton J.M. Roks^4^, Maryam Kavousi^6^, Jeroen Essers^2,3,7^, Jolanda van der Velden^8,16^, Jan H.J. Hoeijmakers^2,17,18^, Dirk J. Duncker^1*^

*****Corresponding author: Dirk J. Duncker MD, PhD; Div. Experimental Cardiology, Dept. Cardiology, Ee-2351; Erasmus University Medical Center; PO Box 2040, 3000 CA Rotterdam, The Netherlands

Tel: +31 10 7038066; Fax: +31 10 7044769; E-mail: d.duncker@erasmusmc.nl

**Electrocardiography.** Electrical properties of the heart were studied by *in vivo* surface electrocardiograms (ECGs) (Remme et al., 2006). Mice (males and females) were sedated with 4% isoflurane and anesthesia was maintained with 1.0-1.5% isoflurane using a nose cone. ECGs were recorded by subcutaneous placement of 23-gauge needles in each limb using the Powerlab acquisition system (ADInstruments). Heart rate, PR interval, P duration, QRS interval and QT interval were determined from lead I using Chart5 Pro analysis software (ADInstruments).

**Echocardiography.** Mice (males and females) were sedated with 4% isoflurane, intubated and connected to a pressure-controlled ventilator (de Waard et al., 2007). Anesthesia was maintained with 2.5% isoflurane and body temperature was kept at 37°C. Cardiac geometry and global function were evaluated by performing 2-D guided short axis M-mode transthoracic echocardiography using the Vevo770 High-Resolution Imaging System (FUJIFILM VisualSonics, Inc.) equipped with a 35-MHz probe. Left ventricular (LV) external and lumen diameters were traced, and heart rate, LV mass and fractional shortening were subsequently calculated using the VisualSonics Cardiac Measurements Package. Data represent averages of at least three cardiac cycles per animal.

**Hemodynamic measurements.** Mice (males and females) were anesthetized and ventilated as described above. Aortic pressure was measured using a 1.4F high-fidelity micro-tipped pressure catheter (Millar, Inc.) inserted into the right carotid artery. Subsequently, the pressure catheter was advanced into the LV to measure LV pressure. Hemodynamic data were recorded and digitized using an on-line data acquisition program (ATCODAS, DATAQ Instruments, Inc.) and stored on a computer for off-line analysis with a program written in MATLAB (MathWorks) (van Deel et al., 2011). Series of at least 6 consecutive beats were selected to calculate heart rate, the maximum rate of rise (LVdP/dt_max_) and fall (LVdP/dt_min_) of LV pressure, the time constant of LV pressure decay (tau) and LV end-diastolic pressure (LVEDP). After completing hemodynamic measurements, mice were euthanized by quick excision of the heart. Hearts were rinsed in ice-cold saline and prepared for further analysis.

**Isometric force measurements in single permeabilized cardiomyocytes.** Samples of liquid nitrogen frozen LV tissues (males and females) were thawed in cold relaxing solution (pH 7.0; 1.0 mM free Mg^2+^, 100 mM KCl, 2.0 mM EGTA, 4.0 mM Mg-ATP, 10 mM imidazole) and cardiomyocytes were mechanically isolated by tissue disruption, as previously described (de Waard et al., 2007; van Deel et al., 2011). To remove all membranes, cardiomyocytes were chemically permeabilized by incubation for 5 minutes in relaxing solution containing 0.5% Triton X-100. The isolated cells were subsequently washed twice in relaxing solution to remove the Triton. Isometric force measurements on single cardiomyocytes were performed at different [Ca^2+^], at 15°C and sarcomere length of 2.2 µm. For this purpose, single cardiomyocytes were attached to a force transducer and a piezoelectric motor using silicone adhesive. Absolute force values were normalized to cardiomyocyte cross-sectional area and expressed as developed tension (kN/m^2^) (de Waard et al., 2007; van Deel et al., 2011).

**Contractile protein composition.** LV samples (males and females) were diluted in sample buffer (pH 6.8; 8.0 M urea, 2.0 M thiourea, 3% SDS (w/v), 75 mM DTT, 0.03% bromophenol blue, 0.05 M Tris-Cl) and fast (α) and slow (β) myosin heavy chain (MHC) isoforms were determined by one-dimensional SDS-gel electrophoresis. MHC isoforms were separated on a 6% acrylamide resolving gel (37.5:1 cross-linked with DATD (Bio-Rad Laboratories, Inc.) and a 3% acrylamide stacking gel (5.6:1 cross-linked with DATD (Bio-Rad Laboratories, Inc.) using a SE600 Hoefer gel system (Pharmacia) at 32 mA constant current (Najafi et al., 2016). Human atrial homogenates, containing both α- and β-MHC, were used as standard. Subsequently, the gels were stained using SYPRO^®^ Ruby and MHC isoform composition was quantified. β-MHC content is expressed as % of total MHC.

**Troponin I phosphorylation.** Cardiac troponin I (cTnI) phosphorylation status in frozen LV tissue samples (males and females) was determined with Phos-tag^TM^ acrylamide (FMS Laboratory) gels, as previously described (Hamdani et al., 2010; Najafi et al., 2016). This methods enables to separate phosphorylated cTnI forms, of similar molecular weight, using alkoxide-bridged dinuclear metal (Mn^2+^) complex as phosphate-binding tag (Phos-tag) molecule, which captures phosphomonoester dianions bound to Ser, Thr and Tyr residues. Thereby, non-phosphorylated, mono-phosphorylated and bis-phosphorylated cTnI forms were separated in 1D-PAGE and subsequently transferred to Western blots. Phosphorylated cTnI species were visualized by using a specific antibody against troponin I, which recognizes both the non-phosphorylated and the phosphorylated forms. Non-failing donor and end-stage failing human samples were included as internal markers. Previous work showed that the three cTnI bands are present in these two samples in which non-failing donor samples exhibit an highly phosphorylated status and end-stage failing samples a low phosphorylated status (Hamdani et al., 2010). Cardiac TnI forms are expressed as % of total phosphorylated cTnI. In addition, phosphorylation of cTnI at PKA sites Ser23/24 was determined by western blot by using Phospho-Troponin I (cardiac; S23/24) primary antibody (1:1000, #4004, Cell signaling Technology). Gapdh (1:5000, #2118, Cell signaling Technology) was used as loading control. Non-failing human donor sample (donor: highly phosphorylated) was used as internal marker. Values are normalized to control.

**Detection of superoxide.** Superoxide (O_2_^-^) generation was measured in homogenized LV tissue (males and females) by lucigenin-enhanced chemiluminescence using a luminometer (Luminoskan, Ascent, Thermo Fisher Scientific) (Kim et al., 2005). LV tissue was homogenized in Krebs-Hepes buffer pH 7.4 (KHB, containing 99.0 mM NaCl, 4.7 mM KCl, 1.2 mM MgSO_4_, 1.0 mM KH_2_PO_4_, 1.9 mM CaCl_2_, 20 mM Hepes, 25 mM NaHCO_3_, 11.1 mM Glucose), supplemented with protease inhibitor cocktail (Complete) using a PRO2000 homogenizer set at maximum speed with 3 bursts of 10 seconds. Protein concentration was determined and 40 µg protein was used per condition (lucigenin; lucigenin+substrate; lucigenin+substrate+inhibitor). After sample equilibration at 37°C for 10 minutes, dark-adapted lucigenin (5 µM, Sigma-Aldrich) was added to the homogenates. Subsequently, light emission was recorded (sum of 500 measurements for 1000 ms each) to determine total O_2_^-^ production. Furthermore, NADPH oxidase (NOX) activity was determined by measuring homogenates with lucigenin and NADPH substrate (100 µM, Sigma-Aldrich). To determine the contribution of NOX to O_2_^-^ production, we measured the difference in O_2_^-^ generation by adding NOX inhibitor VAS2870 (100 µM, Sigma-Aldrich) to homogenates at equilibration, 20 minutes. Temperature was maintained at 37°C, and each experiment was performed in duplicate. All data have been expressed as relative light units per second per gram protein (RLU/sec/g).

**RNA preparation and quantitative real-time PCR.** Total RNA was extracted from frozen LV and liver tissues (males and females) using the RNeasy (Fibrous Tissue) Mini Kit (QIAGEN) according to manufacturer’s protocol. RNA quality and quantity was established using a 2100 Bioanalyser. cDNA was synthesized using iScript^TM^ cDNA Synthesis Kit (Bio-Rad Laboratories, Inc.). Quantitative real-time PCR was performed with specific primers and SensiMix^TM^ SYBR^®^ & Fluorescein Kit (Bio-Rad Laboratories, Inc.). Target gene mRNA levels were expressed relative to housekeeping genes hypoxanthine-guanine phosphoribosyl transferase (*Hprt*), glyceraldehyde-3-phosphate dehydrogenase (*Gapdh*) and tubulin gamma 2 (*tubG2*). Primer sequences are shown in Table S1.

**RNA sequencing.** Total RNA was extracted from frozen LV tissues (males only) using the RNeasy (Fibrous Tissue) Mini Kit (QIAGEN) including DNase digestion according to manufacturer’s protocol. Quality of total RNA was established using a 2100 Bioanalyser, all samples yield a RNA integrity number greater than 8.1. Poly-A enriched directional RNA-Seq libraries were prepared (NEBNext technology) and subsequent single read sequencing was performed using the NextSeq500 System (Illumina; 75 cycles, v 2.5 chemistry). On average each sample contained 31.6±3.4M (mean;sd) single reads.

**Gene expression analysis.** After quality control with FastQC (Andrews, 2010), raw sequencing reads were trimmed using cutadapt (Martin, 2011) and aligned to the mouse reference genome GRCm38 with STAR-2.7.1a (Dobin & Gingeras, 2015). This resulted in 24.7±3.1M (mean;sd) uniquely mapped reads with a minimum length of 73. Remaining reads were counted through the R package GenomicAlignments (Lawrence et al., 2013), followed by differential analysis using DEseq2 (Love, Huber, & Anders, 2014). Genes were considered significantly differentially expressed at absolute fold change >1.5 and FDR adjusted p-value <0.05.

Gene Ontology (GO) and functional clustering analyses were carried out by using multiple databases and software: Ingenuity Pathway Analysis (Kramer, Green, Pollard, & Tugendreich, 2014) and Kyoto Encyclopedia of Genes and Genomes (KEGG) (Kanehisa, Sato, Furumichi, Morishima, & Tanabe, 2019). Significant networks were visualized by BiNGO package (Maere, Heymans, & Kuiper, 2005) in Cystoscope.

**Histology and immunohistochemistry.** Paraffin-embedded LV and liver tissues (males and females) were cut in 4 µm sections, deparaffinized and stained for histological analyses. LV sections were stained with haematoxylin-eosin for a global overview.

Cardiomyocyte size was quantified by performing gomori staining using standard protocol. Cross-sectional areas of 100-350 cardiomyocytes with clearly visible nuclei were measured in 6-10 fields per slide using a microscopy image analysis system (Clemex Vision PE version 7.0, Clemex Technologies Inc.).

To determine myocardial collagen content, picro-sirius red staining was performed using standard protocol. Fibrosis was measured in whole LV sections using a quantitative image analysis system (BioPix iQ software version 3.3.5, BioPix AB) and expressed as percentage of the total myocardial area.

Apoptosis was studied using the terminal deoxynucleotidyl transferase-mediated dUTP nick-end labeling (TUNEL) In Situ Cell Death Detection Kit (Roche Diagnostics). Total and TUNEL positive nuclei were counted in 12 fields (Clemex Vision PE version 7.0, Clemex Technologies Inc.) and apoptosis was expressed as a percentage of total nuclei.

DNA damage was assessed by performing standard immunohistochemistry on LV and liver sections. Sections were de-paraffinized via two changes of xylene and further rehydrated through graded alcohols to distilled water. After blocking endogenous peroxidase activity with 3% hydrogen peroxide in PBS, antigen retrieval was achieved by heating the slides in 10 mmol/l citrate buffer (pH 6) using a rice cooker for 15 minutes. Rabbit monoclonal anti-Phospho-Histone H2A.X primary antibody (1:50; #9718, Cell signaling Technology) was applied overnight at 4°C. HRP-labelled polyclonal goat anti-rabbit Immunoglobulins antibody was used for application of the secondary antibody (1:50; #P044801, Agilent). Signals were developed with Diaminobenzidine (DAB) followed by light nuclear counter staining with haematoxylin. Each test batch was run with a known negative control. Phosphorylation of the H2A.X histone (γH2A.X) was quantified as γH2A.X positive nuclei/mm^2^ (Clemex Vision PE version 7.0, Clemex Technologies Inc.).

***In vivo* µCT-FMT imaging to detect apoptosis and MMP activity.** Twenty-four hours before imaging, mice (males and females) were intravenously injected with the near infrared fluorescent (NIRF) probe MMPSense^TM^ 680 (2 nmol/25 g; PerkinElmer, Inc.). MMPSense^TM^ 680 is a protease activatable fluorescence imaging agent that is activated by key matrix metalloproteinases (MMPs) including MMP-2, -3, -9 and -13. The probe is optically silent in a non-active state and becomes highly fluorescent following protease-mediated activation (Kaijzel et al., 2010). Additionally, mice were intravenously injected with the NIRF probe Annexin-Vivo^TM^ 750, two hours before imaging, (100 µL/25 g; PerkinElmer, Inc.). Annexin-Vivo^TM^ 750 is a targeted fluorescence imaging agent which has been developed to enable *in vivo* visualization and quantification of the membrane-bound phospholipid phosphatidylserine, exposed in the outer leaflet of the cell membrane lipid bilayer during the early stages of apoptosis (van Heerde et al., 2000). Subsequently, mice were anesthetized with 1.5-2.5% isoflurane (O_2_ 1 L/min) and depilated to minimize the interference of fur on the fluorescent signal.

Firstly, mice were imaged with contrast-enhanced Quantum FX Micro-Computed Tomography (µCT; PerkinElmer, Inc.), which was used for anatomical reference (Ale et al., 2012; Grace Liang, Davis Vo, & Nguyen, 2017). For this purpose, mice received a tail vein injection with the iodinated contrast agent eXIA160 (100 µL/25 g; Binitio Biomedical, Inc.). To prevent movement of the animals during imaging, mice were positioned and restrained in the accompanying multimodal animal imaging cassette. Mice were scanned using intrinsic cardiac respiratory gating to reduce artefacts caused by breathing or cardiac motion. After finishing µCT imaging, mice remained under anesthesia and the cassette was transferred to the FMT 2500 fluorescent molecular tomography (FMT) in vivo imaging system (PerkinElmer, Inc.). MMPSense^TM^ 680 imaging was performed using excitation wavelength of 680 nm and emission range of 690-740 nm. Annexin-Vivo^TM^ 750 imaging was performed using excitation wavelength of 750 nm and emission range of 770-800 nm. The multimodal animal imaging cassette facilitates the co-registration of µCT and FMT data by using fiducials. *In vivo* cardiac apoptosis and MMP activity (expressed in pmol/g heart weight) were quantified by merging the FMT data and the µCT data by using the TrueQuant^TM^ software (PerkinElmer, Inc.).

***Ex vivo* imaging to detect MMP activity.** Immediately after µCT-FMT imaging, mice (males and females) were euthanized using an overdose of isoflurane. The hearts were excised, weighed and immersion-fixed in formalin. *Ex vivo* tissue imaging was performed to detect the fluorescent signal of the MMPSense^TM^ 680 probe in the heart using the Odyssey^®^ CLx imaging system (LI-COR^®^ Biosciences). *Ex vivo* cardiac MMP activity was quantified in one plane of the heart and expressed as counts/mm^2^.

***Ex vivo* vascular function.** Following sacrifice, thoracic aortas (males and females) were isolated and carefully cleaned from fat and connective tissue in cold, oxygenated Krebs-Henseleit buffer (pH 7.4; 118.0 mM NaCl, 4.7 mM KCl, 2.5 mM CaCl_2_, 1.2 mM MgSO_4_, 1.2 mM KH_2_PO_4_, 25 mM NaHCO_3_ and 8.3 mM glucose). Vessel segments of ~2 mm length were mounted in 6-mL organ baths (Danish Myo Technology A/S) containing Krebs-Henseleit buffer at 37ºC and oxygenated with 95% O_2_ and 5% CO_2_. Tension was normalized to 90% of the estimated diameter at 100 mmHg effective transmural pressure (Durik et al., 2012). Maximum contractile responses were determined using 100 mmol/L KCl. After washout of KCl, preconstriction was elicited with 30 nmol/L thromboxane-A_2_ analogue U46619. Following preconstriction, relaxation concentration-response curves (CRCs) were constructed to acetylcholine (ACh) and sodium nitroprusside (SNP; 100 µmol/L). Vasodilator responses to ACh and SNP were expressed as percentage of the preconstriction to U46619.

**Human genetic studies.**

**Echocardiography.** For each participant, resting transthoracic M-mode 2-dimensional echocardiography was obtained, using a standardized protocol by two experienced echocardiographers. Echocardiographic examinations were performed by using a commercially available ultrasonography system (AU3 Partner, Esaote Biomedica), with a 3.5/2.5 MHz transducer until October 2003 and from then on a commercially available Acuson Cypress (Siemens), with a 3V2c transducer was used (Kardys et al., 2009). The protocol included 2-dimensional scanning in the parasternal long axis view, parasternal short axis view the apical and subcostal views, M-mode scanning in the parasternal long axis view, and pulsed wave Doppler scanning in the apical four chamber view. All echocardiograms were recorded and assessed offline by the echocardiographers, at the reading center (Rueda-Ochoa et al., 2019). Left ventricular end-diastolic diameter (LVEDD) and left ventricular systolic diameter (LVESD) were assessed and fractional shortening at the endocardium was calculated as (LVEDD-LVESD)/LVEDD*100% (Schiller et al., 1989).

**References**

Ale, A., Ermolayev, V., Herzog, E., Cohrs, C., de Angelis, M. H., & Ntziachristos, V. (2012). FMT-XCT: in vivo animal studies with hybrid fluorescence molecular tomography-X-ray computed tomography. *Nat Methods, 9*(6), 615-620. doi:10.1038/nmeth.2014

Andrews, S. (2010). FastQC: a quality control tool for high throughput sequence data. Retrieved from http://www.bioinformatics.babraham.ac.uk/projects/fastqc

de Waard, M. C., van der Velden, J., Bito, V., Ozdemir, S., Biesmans, L., Boontje, N. M., . . . Duncker, D. J. (2007). Early exercise training normalizes myofilament function and attenuates left ventricular pump dysfunction in mice with a large myocardial infarction. *Circ Res, 100*(7), 1079-1088. doi:10.1161/01.RES.0000262655.16373.37

Dobin, A., & Gingeras, T. R. (2015). Mapping RNA-seq Reads with STAR. *Curr Protoc Bioinformatics, 51*, 11.14.11-11.14.19. doi:10.1002/0471250953.bi1114s51

Durik, M., Kavousi, M., van der Pluijm, I., Isaacs, A., Cheng, C., Verdonk, K., . . . Roks, A. J. (2012). Nucleotide excision DNA repair is associated with age-related vascular dysfunction. *Circulation, 126*(4), 468-478. doi:10.1161/CIRCULATIONAHA.112.104380

Grace Liang, Davis Vo, & Nguyen, P. K. (2017). Fundamentals of Cardiovascular Molecular Imaging: a Review of Concepts and Strategies. *Curr Cardiovasc Imaging Rep*. doi:10.1007/s12410-017-9403-7

Hamdani, N., Borbely, A., Veenstra, S. P., Kooij, V., Vrydag, W., Zaremba, R., . . . van der Velden, J. (2010). More severe cellular phenotype in human idiopathic dilated cardiomyopathy compared to ischemic heart disease. *J Muscle Res Cell Motil, 31*(4), 289-301. doi:10.1007/s10974-010-9231-8

Kaijzel, E. L., van Heijningen, P. M., Wielopolski, P. A., Vermeij, M., Koning, G. A., van Cappellen, W. A., . . . Essers, J. (2010). Multimodality imaging reveals a gradual increase in matrix metalloproteinase activity at aneurysmal lesions in live fibulin-4 mice. *Circ Cardiovasc Imaging, 3*(5), 567-577. doi:10.1161/CIRCIMAGING.109.933093

Kanehisa, M., Sato, Y., Furumichi, M., Morishima, K., & Tanabe, M. (2019). New approach for understanding genome variations in KEGG. *Nucleic Acids Res, 47*(D1), D590-D595. doi:10.1093/nar/gky962

Kardys, I., Deckers, J. W., Stricker, B. H., Vletter, W. B., Hofman, A., & Witteman, J. C. (2009). Echocardiographic parameters and all-cause mortality: the Rotterdam Study. *Int J Cardiol, 133*(2), 198-204. doi:10.1016/j.ijcard.2007.12.031

Kim, Y. M., Guzik, T. J., Zhang, Y. H., Zhang, M. H., Kattach, H., Ratnatunga, C., . . . Casadei, B. (2005). A myocardial Nox2 containing NAD(P)H oxidase contributes to oxidative stress in human atrial fibrillation. *Circ Res, 97*(7), 629-636. doi:10.1161/01.RES.0000183735.09871.61

Kramer, A., Green, J., Pollard, J., Jr., & Tugendreich, S. (2014). Causal analysis approaches in Ingenuity Pathway Analysis. *Bioinformatics, 30*(4), 523-530. doi:10.1093/bioinformatics/btt703

Lawrence, M., Huber, W., Pages, H., Aboyoun, P., Carlson, M., Gentleman, R., . . . Carey, V. J. (2013). Software for computing and annotating genomic ranges. *PLoS Comput Biol, 9*(8), e1003118. doi:10.1371/journal.pcbi.1003118

Love, M. I., Huber, W., & Anders, S. (2014). Moderated estimation of fold change and dispersion for RNA-seq data with DESeq2. *Genome Biol, 15*(12), 550. doi:10.1186/s13059-014-0550-8

Maere, S., Heymans, K., & Kuiper, M. (2005). BiNGO: a Cytoscape plugin to assess overrepresentation of gene ontology categories in biological networks. *Bioinformatics, 21*(16), 3448-3449. doi:10.1093/bioinformatics/bti551

Martin, M. (2011). Cutadapt removes adapter sequences from high-throughput sequencing reads. *EMBnet.journal 17.1*, 10-12. doi:https://doi.org/10.14806/ej.17.1.200

Najafi, A., Sequeira, V., Helmes, M., Bollen, I. A., Goebel, M., Regan, J. A., . . . Van Der Velden, J. (2016). Selective phosphorylation of PKA targets after beta-adrenergic receptor stimulation impairs myofilament function in Mybpc3-targeted HCM mouse model. *Cardiovasc Res, 110*(2), 200-214. doi:10.1093/cvr/cvw026

Remme, C. A., Verkerk, A. O., Nuyens, D., van Ginneken, A. C., van Brunschot, S., Belterman, C. N., . . . Bezzina, C. R. (2006). Overlap syndrome of cardiac sodium channel disease in mice carrying the equivalent mutation of human SCN5A-1795insD. *Circulation, 114*(24), 2584-2594. doi:10.1161/CIRCULATIONAHA.106.653949

Rueda-Ochoa, O. L., Smiderle-Gelain, M. A., Rizopoulos, D., Dhana, K., van den Berge, J. K., Echeverria, L. E., . . . Kavousi, M. (2019). Risk factors for longitudinal changes in left ventricular diastolic function among women and men. *Heart, 105*(18), 1414-1422. doi:10.1136/heartjnl-2018-314487

Schiller, N. B., Shah, P. M., Crawford, M., DeMaria, A., Devereux, R., Feigenbaum, H., . . . et al. (1989). Recommendations for quantitation of the left ventricle by two-dimensional echocardiography. American Society of Echocardiography Committee on Standards, Subcommittee on Quantitation of Two-Dimensional Echocardiograms. *J Am Soc Echocardiogr, 2*(5), 358-367. doi:10.1016/s0894-7317(89)80014-8

van Deel, E. D., de Boer, M., Kuster, D. W., Boontje, N. M., Holemans, P., Sipido, K. R., . . . Duncker, D. J. (2011). Exercise training does not improve cardiac function in compensated or decompensated left ventricular hypertrophy induced by aortic stenosis. *J Mol Cell Cardiol, 50*(6), 1017-1025. doi:10.1016/j.yjmcc.2011.01.016

van Heerde, W. L., Robert-Offerman, S., Dumont, E., Hofstra, L., Doevendans, P. A., Smits, J. F., . . . Reutelingsperger, C. P. (2000). Markers of apoptosis in cardiovascular tissues: focus on Annexin V. *Cardiovasc Res, 45*(3), 549-559. doi:10.1016/s0008-6363(99)00396-x
